# Identifying Frailty in Older Adults Receiving Home Care Assessment Using Machine Learning: Longitudinal Observational Study on the Role of Classifier, Feature Selection, and Sample Size

**DOI:** 10.2196/44185

**Published:** 2024-01-31

**Authors:** Cheng Pan, Hao Luo, Gary Cheung, Huiquan Zhou, Reynold Cheng, Sarah Cullum, Chuan Wu

**Affiliations:** 1 Department of Computer Science The University of Hong Kong Hong Kong China (Hong Kong); 2 Department of Social Work and Social Administration The University of Hong Kong Hong Kong China (Hong Kong); 3 Department of Psychological Medicine School of Medicine The University of Auckland Auckland New Zealand

**Keywords:** machine learning, logistic regression, frailty, older adults, home care, sample size, features, data set, model, home care, mortality prediction, assessment

## Abstract

**Background:**

Machine learning techniques are starting to be used in various health care data sets to identify frail persons who may benefit from interventions. However, evidence about the performance of machine learning techniques compared to conventional regression is mixed. It is also unclear what methodological and database factors are associated with performance.

**Objective:**

This study aimed to compare the mortality prediction accuracy of various machine learning classifiers for identifying frail older adults in different scenarios.

**Methods:**

We used deidentified data collected from older adults (65 years of age and older) assessed with interRAI-Home Care instrument in New Zealand between January 1, 2012, and December 31, 2016. A total of 138 interRAI assessment items were used to predict 6-month and 12-month mortality, using 3 machine learning classifiers (random forest [RF], extreme gradient boosting [XGBoost], and multilayer perceptron [MLP]) and regularized logistic regression. We conducted a simulation study comparing the performance of machine learning models with logistic regression and interRAI Home Care Frailty Scale and examined the effects of sample sizes, the number of features, and train-test split ratios.

**Results:**

A total of 95,042 older adults (median age 82.66 years, IQR 77.92-88.76; n=37,462, 39.42% male) receiving home care were analyzed. The average area under the curve (AUC) and sensitivities of 6-month mortality prediction showed that machine learning classifiers did not outperform regularized logistic regressions. In terms of AUC, regularized logistic regression had better performance than XGBoost, MLP, and RF when the number of features was ≤80 and the sample size ≤16,000; MLP outperformed regularized logistic regression in terms of sensitivities when the number of features was ≥40 and the sample size ≥4000. Conversely, RF and XGBoost demonstrated higher specificities than regularized logistic regression in all scenarios.

**Conclusions:**

The study revealed that machine learning models exhibited significant variation in prediction performance when evaluated using different metrics. Regularized logistic regression was an effective model for identifying frail older adults receiving home care, as indicated by the AUC, particularly when the number of features and sample sizes were not excessively large. Conversely, MLP displayed superior sensitivity, while RF exhibited superior specificity when the number of features and sample sizes were large.

## Introduction

Frailty is a syndrome characterized by an increased vulnerability to adverse health outcomes, including falling, hospitalization, physical decline, and mortality [1]. Frailty should be detected as early as possible since it is potentially preventable and treatable [2]. In community settings, timely identification of frailty allows the implementation of early interventions that could reduce care costs and improve the “ability of older persons to age in place” [3]. In clinical and long-term care settings, identifying frail older adults could facilitate more individualized and tailored health care planning [4,5]. Therefore, efficient and accurate clinical tools are pivotal to the early identification of frailty among at-risk older adults.

Numerous methods have been applied to measure frailty. A recent systematic review identified 21 conceptual definitions and 59 operational definitions of frailty from 68 studies [6]. This review concluded that definitions of frailty can be classified into 3 categories focusing on different dimensions. The first is represented by the Cardiovascular Health Study (CHS) Index based on Fried’s “frailty phenotype” model, which focuses on the physical dimensions of frailty [7-10]. The second category is represented by the Frailty Index, originally proposed by Rockwood and Mitnitski [11,12], which considers frailty as a syndrome capturing the accumulative gradient of deficits. This category of definitions covers other dimensions of frailty, including cognitive, psychological, nutritional, and social factors [11,13]. The third category considers the social dimension of frailty, which has a significant relationship with undesirable adverse health outcomes [14-16]. Despite differences in theoretical frameworks adopted by different frailty measures, existing frailty indices are typically constructed by summing up the number of deficits or scores of assessment items using equal weighting. Arguably, different deficits from various domains may impact overall frailty status differently, and these differences should be considered when measuring frailty. In addition to accounting for the multifactorial nature of frailty, a successful definition of frailty [12] must demonstrate satisfactory criterion validity. Since frailty is noncontroversially linked with vulnerability, a valid measure of frailty must accurately predict adverse outcomes, such as death, institutionalization, hospitalization, physical decline, and falls. Mortality is the most objective measure that is less susceptible to measurement error and, thus, is the most widely used outcome for assessing the predictive validity of frailty measures [9,17-20].

Routinely collected data from health information systems have become increasingly available in recent years, and clinical big data analytics featured by machine learning techniques are ever-evolving [21-23]. In contrast to conventional regression approaches, classifiers used in machine learning, such as random forest (RF), support vector machines, and neural networks, have the advantages of learning and generating predictions by examining large-scale databases of complex clinical information [18,20,24-26]. Therefore, it is reasonable to hypothesize that applying machine learning techniques to large-scale data collected from health information systems can improve the accuracy of mortality prediction for identifying frail older persons who may benefit from early interventions. However, the literature remains unclear whether machine learning techniques can outperform conventional regression models in identifying frail older adults [18,19,27].

In this study, we used routinely collected health information of people receiving home care in New Zealand from interRAI-Home Care (interRAI-HC) assessment to examine the performance of various machine learning classifiers in mortality prediction for identifying frailty. In this study, we conducted a simulation study to address the following research questions: (1) does the performance of machine learning models exceed that of the interRAI-HC Frailty Scale, which was developed using conventional regression models [28], in identifying frailty? (2) what are the performances of different machine learning models? and (3) what are the effects of sample size, number of features, and the ratio of training to test data on predictive accuracy?

## Methods

### Data Source and Participants

In this retrospective observational study, we used deidentified health information routinely collected from older adults assessed using the interRAI-HC assessment (version 9.1). The interRAI-HC assessment was developed by a network of health researchers in over 35 countries [29]. interRAI assessments are mandatory in aged residential care and home and community services for older people living in the community in New Zealand. Our participants were from all 20 District Health Boards in New Zealand and included all community-dwelling older adults who were receiving public-funded home care or assessed for long-term aged residential care. Trained interRAI assessors collect comprehensive health information on older adults, including their demographic, clinical, psychosocial, and functional details. The interRAI-HC assessment embeds over 100 potential deficits of older adults that can be used to identify frailty. Table S1 in Multimedia Appendix 1 summarizes the variables used for identifying frail older adults. Ethnicity was not included to increase generalizability beyond New Zealand.

We included adults 65 years of age or older for whom at least 1 interRAI-HC assessment had been completed between January 1, 2012, and December 31, 2016. Only the most recent interRAI-HC assessment (defined as the index assessment) of each individual within this period was used in the analysis and the date of the most recent assessment was defined as the index date. The individuals were followed from the index date until the date of death or December 31, 2019, whichever came first.

### Ethical Considerations

The University of Auckland Human Participant Ethics Committee provided ethics approval for this study (023801).

### Measures

#### Outcomes

Outcomes of interest were 6-month and 12-month mortality. Mortality data were retrieved from the Ministry of Health Mortality Dataset that contains information of all registered deaths in New Zealand. These two-time points were chosen because (1) older adults receiving home care are associated with a higher risk of mortality and shorter survival compared with their counterparts who are not receiving home care and (2) these are outcomes commonly used in previous studies examining the association between frailty and mortality [30-33] and few previous studies using interRAI data [34-36].

#### Features Used in Machine Learning Models

Features of interest included 138 interRAI-HC assessment items covering 11 broad domains, demographics, cognition, communication and vision, mood and behavior, psychosocial well-being, functional status, continence, disease diagnoses, health conditions, oral and nutrition status, and skin conditions. Table S1 in Multimedia Appendix 1 presents the details of features used to identify frail older individuals.

Assessment items that had a missing percentage of over 10% were excluded from this study. Multiple interRAI-HC assessment variables with a response indicating that the activity did not occur during the assessment were considered missing, and the missing data imputation was implemented for these responses.

#### Established Frailty Scales (Benchmark)

The interRAI-HC Frailty Scale was used as the benchmark for evaluating the predictive performance of machine learning algorithms. The interRAI-HC Frailty Scale was developed and validated using assessments collected from multiple and diverse countries worldwide [28]. Table S2 in Multimedia Appendix 1 summarizes the variables used in constructing the interRAI-HC Frailty Scale.

### Machine Learning and Logistic Regression Models

We applied 3 state-of-the-art machine learning models and regularized logistic regression to predict 6-month and 12-month mortality using the features available from interRAI-HC. The RF is a machine learning algorithm that uses decision trees [37]. The RF provides highly accurate predictions with a very large number of input variables [38]. The eXtreme Gradient Boosting (XGBoost) is an optimized algorithm designed to implement parallel tree boosting that can predict results extremely efficiently and accurately based on its scalability and efficiency in all scenarios [39]. Multilayer perceptron (MLP) is one of the most popular paradigms of artificial neural networks. MLP decreases the output error by adjusting the weights of predictive variables through an iterative learning process [40].

Regularized logistic regression is a variant of logistic regression using regularization to prevent overfitting and improve the performance of logistic regression. Two popular types of regularized logistic regressions are Least Absolute Shrinkage and Selection Operator (LASSO) regularization with the L1 penalty [41] and Ridge regularization with the L2 penalty [42].

In this study, we implemented hyperparameter tuning to regularize logistic regression (hereafter referred to as logistic regression), RF, MLP, and XGBoost by performing a randomized grid search using all home care (HC) assessment items. The best hyperparameters for each classifier were determined by 10-fold cross-validation (Table S5 in Multimedia Appendix 1). We used iterative imputation [43] to handle the missing values and the default threshold of 0.5 was used in training [27]. We conducted a sensitivity analysis to compare the performance of the models with and without imputation in selected conditions, that is, only the minimum and maximum sample sizes and the number of features were selected for comparison due to the expensive computation power required.

**Figure 1:**
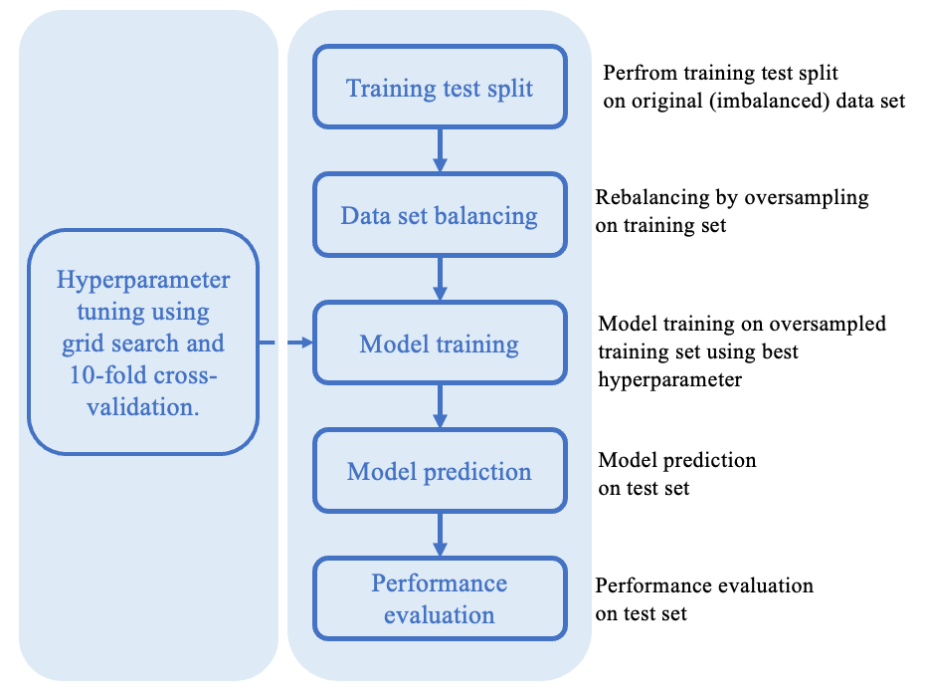
Illustration of the process of data preprocessing, training, prediction, and evaluation.

The preliminary results suggested that our data are imbalanced, as the majority of individuals survived within 6 or 12 months. We therefore rebalanced the training data (but not the test data) using random oversampling [44], while keeping the test data unchanged. Our primary findings are presented with the results obtained after rebalancing the data. The results using the original imbalanced data set can be found in Multimedia Appendix 1. Specifically, to initiate the hyperparameter tuning process, we performed hyperparameter tuning using grid search. For each combination of hyperparameters, within each iteration of the 10-fold cross-validation loop, we applied oversampling to the training set, and the model was trained on the oversampled training set using the current combination of hyperparameters. The model’s performance was evaluated on the validation set. After all combinations of hyperparameters have been evaluated, we selected the combination that gave the best average performance. The process of data preprocessing, training, prediction, and evaluation is illustrated in Figure 1.

### Simulation Design

We conducted a Monte Carlo simulation to compare the performance of different machine learning methods and logistic regression under different experimental conditions, characterized by different sample sizes, the number of features, and training test split ratios. There were 72 experimental conditions for each model (4 sample sizes, 6 feature numbers, and 3 training test split ratios). Each of these conditions was repeated 1000 times to assess their variability. We used sample sizes equaling 1000, 4000, 16,000, and 95,042; the number of features equaling 10, 20, 30, 40, 80, and 138; and training test split ratios equaling 7:3, 8:2, and 9:1 in our simulation. We selected these sample sizes and feature numbers because they are commonly encountered in existing studies on frailty measurement [17,19,45-48] and are values that are testable using the current database. The training split ratios are widely used in studies using machine learning [18,27,36,49,50]. We chose a limited number under each domain to keep the simulations to a manageable scale.

### Evaluation of Model Performance

We randomly split the data into a training sample and a test sample with different training test ratios. We evaluated model performances using the test sample. The discrimination ability of each classifier was measured by the area under the curve (AUC) [51], sensitivity, (also referred to as the true positive rate), and specificity (also known as the true negative rate) as the primary criteria because these are criteria widely accepted by the clinicians. Since frailty is reversible and may be attenuated by noninvasive interventions such as exercise, reduction of polypharmacy, and adequate nutrition [52], high sensitivity is viewed as more important than high specificity in this context if a trade-off needs to be made. *F*_1_-score [53], accuracy and precision (also called positive predictive value) [47,54,55] were also constructed and assessed to allow comparisons with studies that reported only these outcomes. Note, that as each experimental condition was repeated 1000 times to address the potential impact of randomization, we computed the mean and SDs of all performance indices across 1000 replications. The 95% CI for the performance metrics was computed from 1000 runs for each scenario.

## Results

We included 95,042 older adults after excluding 4676 individuals who were younger than 65 years of age and 51 individuals with incorrect records (eg, the date of death was earlier than the assessment date, invalid date of birth, or an incorrect assessment date). Table 1 summarizes the characteristics of study subjects, stratified by whether the person died within 6 months. About half of the subjects were aged between 80 and 89 years (80-84 years: n=21,947, 23.09%; 85-89 years: n=23,906, 25.15%). Women accounted for 57,580 (60.58%) of the sample, and 83,590 (87.95%) were European. A total of 12,401 (13.05%) subjects died within 6 months following the index assessment. Table S19 in Multimedia Appendix 1 documents the characteristics of the study subjects, stratified by whether the person died within 1 year.

Table S4 in Multimedia Appendix 1 presents the results of the sensitivity analysis comparing the performance of the models with and without imputation. The findings suggest that the data imputation was necessary as the imputed data set outperformed the unimputed data set in most of the conditions tested.

**Figure 2:**
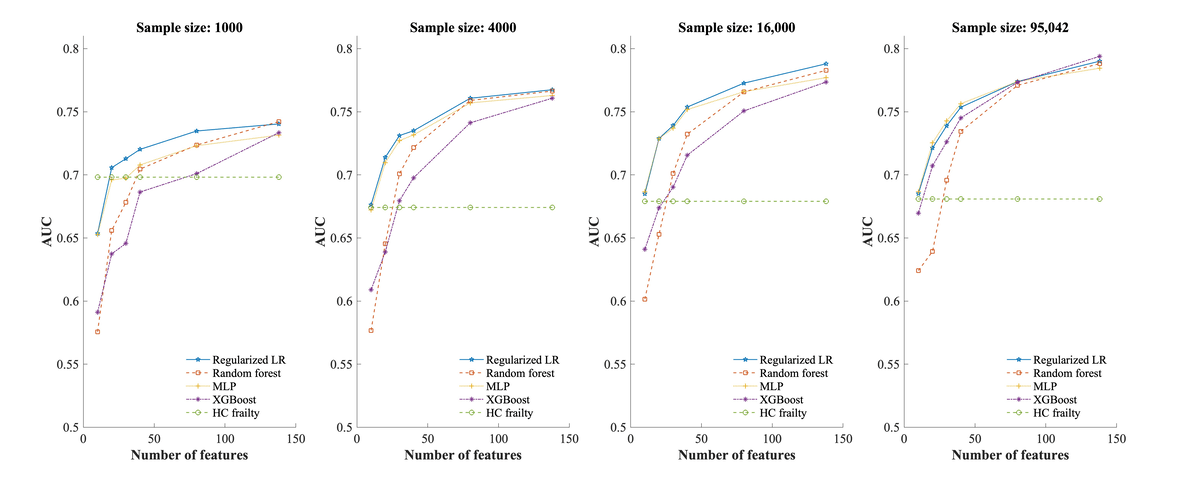
Average AUCs of classifiers and frailty scale for 6-month mortality prediction on balanced data set. AUC: area under the curve; HC: home care; LR: logistic regression; MLP: multilayer perceptron; XGBoost: extreme gradient boosting.

After comparing the performance of penalty terms none, L1, and L2, the LASSO regression regularization (L1) and Ridge regularization (L2) were used in 6-month and 12-month mortality prediction, respectively. We compared the average AUC of each classifier as the number of features increased for 6-month mortality prediction (Figure 2). Overall, the performance of all methods improved considerably as the number of features increased. Specifically, in most scenarios, when the number of features increased to 30, four classifiers demonstrated significantly higher AUC than the interRAI-HC Frailty Scale. LASSO regression generally demonstrated higher or comparable AUC than RF, MLP, and XGBoost. However, in the specific scenario where the sample size was 95,042 and the number of features was 40 or less, MLP showed a slightly better average AUC than LASSO regression. In addition, when the sample size was 95,042, and the number of features increased to 138, XGBoost achieved the highest average AUC of 0.79 (95% CI 0.79-0.80).

**Figure 3:**
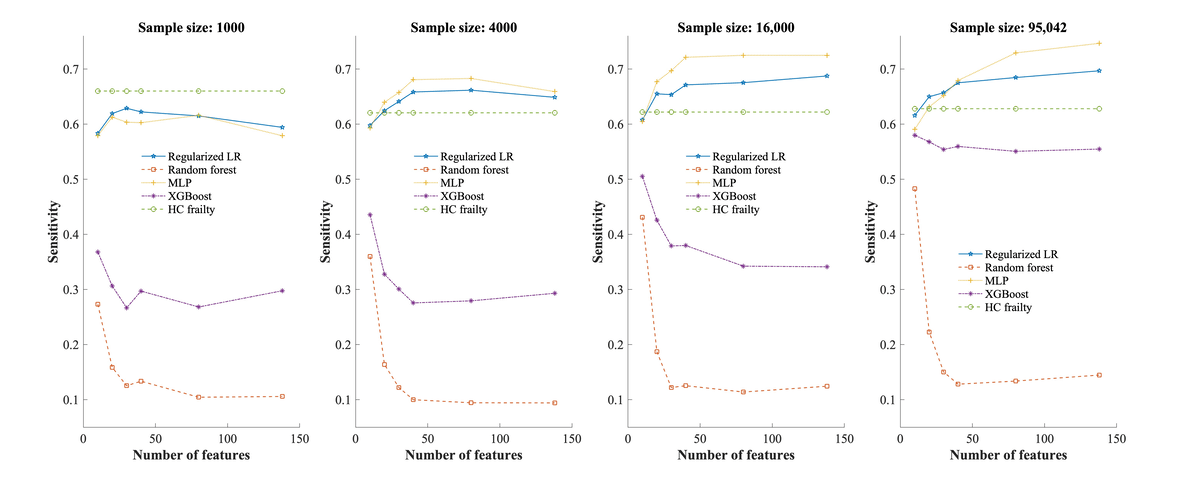
Average sensitivities of classifiers and frailty scale for 6-month mortality prediction on balanced data set. HC: home care; LR: logistic regression; MLP: multilayer perceptron; XGBoost: extreme gradient boosting.

**Figure 4:**
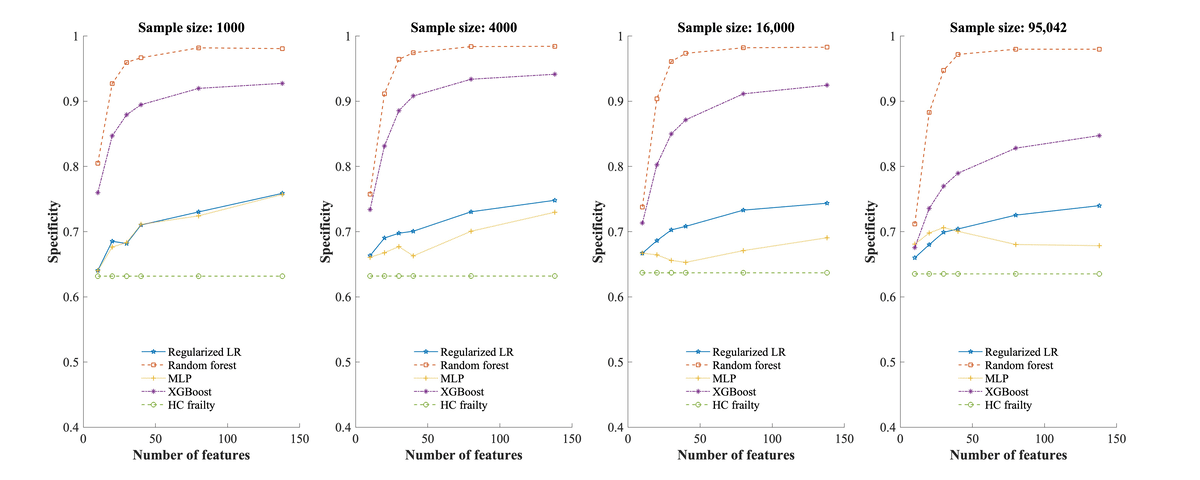
Average specificities of classifiers and frailty scale for 6-month mortality prediction on balanced data set. HC: home care; LR: logistic regression; MLP: multilayer perceptron; XGBoost: extreme gradient boosting.

Figure 3 shows the average sensitivities across all experimental conditions. The 3 machine learning classifiers and LASSO regression had lower sensitivities than the interRAI-HC frailty scale when the sample size was 1000. As the sample size increased to 4000 and the number of features increased to 20, MLP and LASSO regression outperformed the benchmark scale with the highest average sensitivity of 0.77 (95% CI 0.72-0.79) observed in MLP when the sample size was 95,042, and the number of features was 138. Meanwhile, all classifiers demonstrated higher average specificities than the interRAI-HC Frailty Scale in all scenarios (Figure 4). The RF and XGBoost demonstrated higher specificities than LASSO regression, with RF achieving the highest average specificities of 0.98 (95% CI 0.98-0.98) when the sample size was 95,042 and the number of features was 138.

**Figure 5:**
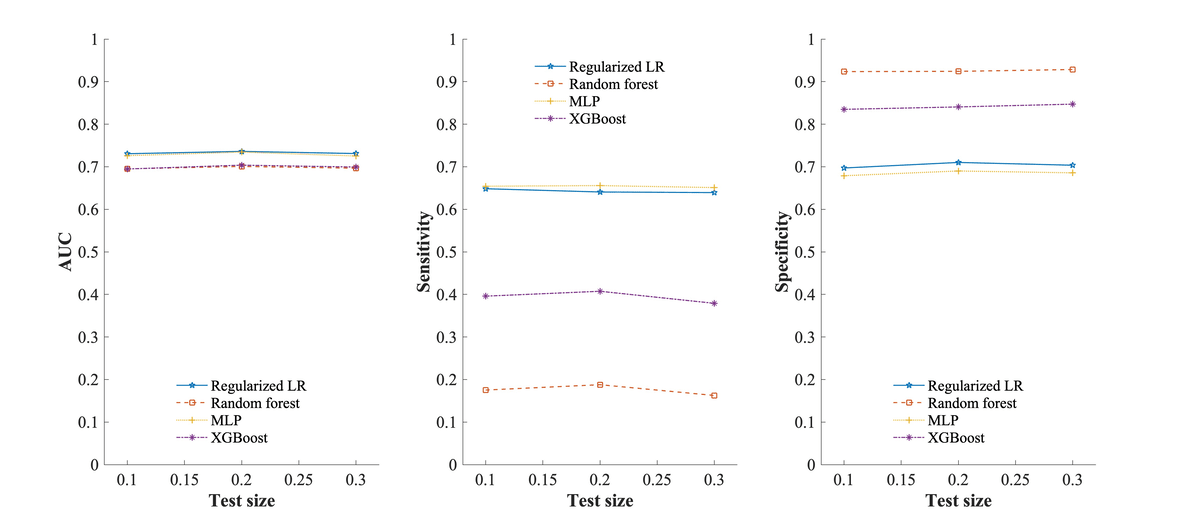
Average AUCs, sensitivities, and specificities of frailty scales for 6-month mortality prediction by test sizes on balanced data set. AUC: area under the curve; LR: logistic regression; MLP: multilayer perceptron; XGBoost: extreme gradient boosting.

Based on the simulation results, it was observed that the test size ratios did not have a significant impact on the average AUC, sensitivities, and specificities, as shown in Figure 5. The 12-month and 6-month mortality predictions were comparable (Figures S1-S4 in Multimedia Appendix 1). However, the overall performance of logistic regression on the 12-month mortality prediction was worse than the 6-month prediction. Compared to the 6-month mortality prediction, machine learning classifiers performed slightly better average sensitivities and worse average AUCs and specificities on 12-month mortality prediction. Tables S5-S18 and S20-S33 in Multimedia Appendix 1 summarize AUC, sensitivity, specificity, *F*_1_-score, accuracy, and precision.

Our simulation was also conducted on the imbalanced data set, and we observed a similar result in terms of average AUCs. Regularized logistic regression had a higher AUC than XGBoost, MLP, and RF, especially when the number of features was less than or equal to 80 and the sample size was less than or equal to 16,000. However, as the number of features and sample sizes increased, XGBoost slightly outperformed regularized logistic regression. In terms of sensitivities, regularized logistic regression significantly outperformed machine learning classifiers in all scenarios, while machine learning classifiers had higher specificities than regularized logistic regression in all scenarios. Additionally, the findings for 12-month and 6-month mortality prediction were similar. However, machine learning classifiers performed slightly better in average sensitivities, but worse in average AUCs and specificities for 12-month mortality prediction compared to 6-month mortality prediction. Multimedia Appendix 1 has been included to summarize the results of the imbalanced data set (Tables S34-S62 and Figures S9-S12 in Multimedia Appendix 1).

**Table 1:** Sample characteristics of 6-month mortality.

Characteristics		HC^a^ (N=95,042)	6-month deceased (n=12,401)	6-month survived (n=82,641)
**Age (years)**				
	65-69, n (%)	5906 (6.21)	693 (5.59)	5213 (6.31)
	70-74, n (%)	9623 (10.12)	1065 (8.59)	8558 (10.36)
	75-79, n (%)	15,284 (16.08)	1770 (14.27)	13,514 (16.35)
	80-84, n (%)	21,947 (23.09)	2662 (21.47)	19,285 (23.34)
	85-89, n (%)	23,906 (25.15)	3312 (26.71)	20,594 (24.92)
	90-94, n (%)	14,370 (15.12)	2160 (17.42)	12,210 (14.77)
	95-99, n (%)	3594 (3.78)	654 (5.27)	2940 (3.56)
	≥100, n (%)	412 (0.43)	85 (0.69)	327 (0.40)
	Mean (SD)	82.66 (7.61)	83.59 (7.71)	82.52 (7.59)
**Gender, n (%)**				
	Female	57,580 (60.58)	6362 (51.30)	51,218 (61.98)
	Male	37,462 (39.42)	6039 (48.70)	31,423 (38.02)
**Ethnicity, n (%)**				
	European	83,590 (87.95)	11,128 (89.73)	72,462 (87.68)
	Maori	5321 (5.60)	730 (5.89)	4591 (5.56)
	Pacific Island	2948 (3.10)	267 (2.15)	2681 (3.24)
	Asian	2304 (2.42)	197 (1.59)	2107 (2.55)
	Middle eastern or Latin American or African	352 (0.37)	25 (0.20)	327 (0.40)
	Other ethnicity	527 (0.55)	54 (0.44)	473 (0.57)
**Marital status, n (%)**				
	Married or civil union or de facto	82,401 (86.70)	10,936 (88.19)	71,465 (86.48)
	Never married	4486 (4.72)	539 (4.35)	3947 (4.78)
	Widowed	2116 (2.23)	240 (1.94)	1876 (2.27)
	Separated or divorced	5999 (6.31)	683 (5.51)	5316 (6.43)
	Others	40 (0.04)	3 (0.02)	37 (0.04)

^a^HC: home care.

## Discussion

### Principal Findings

In this retrospective study of older adults with the mandated standardized interRAI-HC assessment in New Zealand, we performed a series of simulations to evaluate the role of machine learning classifiers, features, and sample sizes on mortality prediction in identifying frail older individuals. We found that in most scenarios, particularly when dealing with large sample sizes and large numbers of features, 4 classifiers demonstrated significantly higher AUCs and sensitivities compared to the interRAI-HC Frailty Scale. All classifiers showed higher average specificities than the interRAI-HC Frailty Scale across all scenarios. Our simulation results showed that the predictive performance differed significantly by using different numbers of randomly selected features, varied sample sizes, and performance measures. Compared to machine learning classifiers, that is, RF, MLP, and XGBoost, logistic regressions provided higher average AUCs on 6-month mortality prediction when the number of features and sample sizes were not excessive. Even with a high number of features and very large samples, only slight improvements in average AUCs were observed in MLP and XGBoost. However, when the number of features and sample sizes were large, MLP demonstrated superior sensitivity, whereas RF exhibited superior specificity.

### Interpretation in the Light of the Published Literature

In recent years, machine learning techniques have started to be used in various large-scale health care data sets to develop predictive algorithms for various adverse health outcomes, including hospitalization, mortality, and frailty in different populations [18,20,24,56]. For example, a recent study showed that by using only 10 or 11 features and 592 study subjects, the machine learning classifier support vector machines identified frail older adults with over 75% accuracy [45]. Another study also showed that by using 16 features, the machine learning classifier gradient boosting achieved 90% AUC on 30-day mortality prediction in patients with heart failure [19]. However, due to limitations in sample size and the number of available features, no study has systematically examined the role of methodological and database factors in the performance of various machine learning techniques. To our knowledge, our study is the first to use high-quality health care data of older adults receiving home care to investigate the performance of machine learning classifiers in identifying frail persons compared to an existing clinical scale and conventional logistic regressions. It is also the first to elucidate to what extent the performance is associated with the choice of classifier, sample size, and the number of features.

Contrary to our hypothesis, the application of machine learning classifiers did not improve the performance of mortality prediction for identifying frail older adults, as evaluated by AUC. This finding indicates that regularized logistic regression can perform sufficiently well and save computational resources when a well-structured, high-quality data source is used. One possible explanation for this result could be the nature of the features, as most of the items used to identify frail older adults are binary. Another reason may be the high reliability of interRAI-HC data [21,57]. In a previous study that also used machine learning to predict frailty status, logistic regression demonstrated comparable or higher performance in various scenarios [27]. This previous study suggested that the tree-based classifiers performed better if the data set was of low quality and contained bad features, and that MLP could generally show a greater performance if the data set is large enough and has complex structure with many layers. In our study, the reason why MLP did not show superior performance on average AUCs could be due to only 1 hidden layer being used.

On the other hand, when the number of features and sample sizes were large, machine learning models demonstrated better performance than logistic regression on both sensitivity and specificity. Specifically, MLP exhibited superior sensitivity, which means that it was more effective at accurately identifying frail older adults receiving home care and were at high risk of adverse health outcomes. In contrast, RF demonstrated superior specificity, which means that it was better at correctly identifying those who were not at high risk of adverse health outcomes. In the context of frailty, where interventions such as exercise, reduction of polypharmacy, and adequate nutrition can attenuate and even reverse the condition [52], high sensitivity is considered more important than high specificity if a trade-off between the 2 measures is required.

Our study revealed that the RF and XGBoost classifiers had significantly lower sensitivities and higher specificities than logistic regression, while MLP had higher sensitivities and lower specificities. This finding is consistent with previous studies on identifying frailty. For example, a study using various machine learning methods to develop predictive models for frailty conditions in older individuals based on an administrative health database [18] observed lower sensitivities and higher specificities for RF when predicting urgent hospitalization, and higher sensitivities and lower specificities for MLP when predicting various health outcomes, including mortality, fracture, and preventable hospitalization. Another similar study that developed a validated case definition of frailty using machine learning classifiers [27] found significantly lower sensitivities and higher specificities for XGBoost and RF compared to logistic regression on balanced data using the default threshold. These findings collectively suggest that identifying frailty using machine learning techniques remains challenging and future research is warranted to investigate the performance of machine learning models in other populations and care settings.

### Implications for Research, Policy, and Practice

We did not identify any machine learning classifier that performed consistently better than the others. The best classifier differed across experimental conditions. Our results demonstrate that the advantages of using machine learning techniques to identify frail older adults become more apparent as the sample size and number of features increase. The logistic regression demonstrated higher or comparable AUC compared to machine learning classifiers in most scenarios. This differs from previous studies that show that machine learning classifiers outperformed logistic regression or its variants in predicting adverse health outcomes [18,20,24-26]. With a sample size of 95,042 and 138 features, Ridge logistic regression achieved an average AUC of 0.77 for 12-month mortality prediction. A logistic regression-based model developed by a previous study using interRAI-HC assessments of older persons in the New Zealand cohort targeting older individuals with complex comorbidities achieved an average AUC slightly higher (<0.01) than our result for 12-month mortality prediction [36]. The previous study used a slightly larger sample size of 104,436 and used a feature selection process to include only the features contributing over 1% to the performance. This may imply that a larger sample size and a feature selection process could further improve the predictive performance of logistic regression.

### Strengths and Limitations

Our study used data collected from the interRAI instruments, standardized assessment instruments that have been developed by a collaborative network of health care professionals [21]. The interRAI instruments have been adopted in several jurisdictions to improve the quality of care for long-term care recipients, including Canada, Finland, Belgium, Italy, and Hong Kong. Therefore, the findings from this study may inform the identification of frail older adults for early interventions in similar care settings using interRAI assessments.

Our study has limitations. First, a successful measure of frailty should demonstrate satisfactory criterion validity against various adverse outcomes such as mortality, disability, hospitalization, and nursing home placement. Our study considered only mortality; therefore, it did not examine the accuracy of machine learning algorithms in predicting other adverse outcomes. Furthermore, we considered only 6- and 12-month mortality, resulting in an imbalanced data set that may yield higher specificity when using machine learning algorithms. It is also unclear whether the results can be extrapolated to other time intervals, such as 2 and 3 years. Further studies are needed to evaluate the prediction power of frailty against other critical outcomes. Second, the samples used in this study were limited to older adults receiving home care in New Zealand and most participants were Europeans. Future studies are warranted to assess the generalizability of this study’s findings. Third, we applied only 3 machine learning classifiers, chosen because they demonstrated better performance in several previous studies. The performance of other machine learning algorithms compared to regularized logistic regression was not investigated. Therefore, our conclusions are limited to the 3 algorithms examined. Fourth, calibration was not performed when training a machine learning classifier due to its additional computational costs, which may have affected the evaluation of model performance. The purpose of this study is to examine the impact of sample size and feature selection on the overall performance of each classifier in identifying frailty in older adults, rather than focusing on probability estimation or the quality of explanations provided by each model. It is worth noting that a recently published study [58] found that uncalibrated RF and XGBoost models performed similarly or even better than calibrated models in terms of accuracy and AUC. Therefore, the impact of calibration on our findings may not be severe. Finally, comparing the main features that affect the performance of different algorithms may improve the understanding of the construct of frailty. However, since the features in our simulation design were randomly selected across 1000 replications, the most important features identified from each run-in condition were not directly comparable. Therefore, we did not carry out further investigation on feature importance under different conditions.

### Conclusions

Machine learning classifiers demonstrate considerable variability in prediction performance when assessed using different metrics. Regularized logistic regression is a reliable model for identifying frail older adults receiving home care, as indicated by the AUC, especially when the number of features and sample sizes are not excessively large. Conversely, MLP shows superior sensitivity, while RF demonstrates superior specificity when the number of features and sample sizes is large.

## Appendix

Multimedia Appendix 1 Supplementary experiments: features and results.

## Abbreviations

AUC: area under the curve
CHS: Cardiovascular Health Study
HC: home care
interRAI-HC: interRAI-Home Care
LASSO: Least Absolute Shrinkage and Selection Operator
MLP: multilayer perceptron
RF: random forest
XGBoost: extreme gradient boosting
