# Supplementary material for: Identifying Frailty in Older Adults Receiving Home Care Assessment Using Machine Learning: Longitudinal Observational Study on the Role of Classifier, Feature Selection, and Sample Size
Source: JMIR AI. 2024 Jan 31;3:e44185. doi: 10.2196/44185 (PMC11041467; doi:10.2196/44185)
Supplement: Multimedia Appendix 1 [file ai_v3i1e44185_app1.docx]

Table of Contents

[Features utilized for frailty scale development (Table A1) 4](#_Toc150860415)

[Features utilized for interRAI Home Care Frailty Scale (Table A2) 9](#_Toc150860416)

[Best hyperparameters for each machine learning classification model on balanced data set (Table A3) 10](#_Toc150860417)

[6-month mortality prediction on balanced data set 11](#_Toc150860418)

[Sensitivity Analysis on Imputed and Unimputed data sets (Table A4) 11](#_Toc150860419)

[Performance evaluation of frailty scale (Table A5 & A6) 12](#_Toc150860420)

[Average AUCs of classifiers - test size 0.1(Table A7) 13](#_Toc150860421)

[Average AUCs of classifiers - test size 0.2 (Table A8) 14](#_Toc150860422)

[Average AUCs of classifiers - test size 0.3 (Table A9) 15](#_Toc150860423)

[Average sensitivities and specificities of classifiers - test size 0.1 (Table A10) 16](#_Toc150860424)

[Average sensitivities and specificities of classifiers - test size 0.2 (Table A11) 17](#_Toc150860425)

[Average sensitivities and specificities of classifiers - test size 0.3 (Table A12) 18](#_Toc150860426)

[Average F1-scores of classifiers - test size 0.1 (Table A13) 19](#_Toc150860427)

[Average F1-scores of classifiers - test size 0.2 (Table A14) 20](#_Toc150860428)

[Average F1-scores of classifiers - test size 0.3 (Table A15) 21](#_Toc150860429)

[Average accuracies and precisions of classifiers - test size 0.1 (Table A16) 22](#_Toc150860430)

[Average accuracies and precisions of classifiers - test size 0.2 (Table A17) 23](#_Toc150860431)

[Average accuracies and precisions of classifiers - test size 0.3 (Table A18) 24](#_Toc150860432)

[Sample characteristics of 12-month mortality (Table A19) 25](#_Toc150860433)

[12-month mortality prediction on balanced data set 26](#_Toc150860434)

[Average AUCs of classifiers and frailty scale (Figure A1) 26](#_Toc150860435)

[Average sensitivities of classifiers and frailty scale (Figure A2) 27](#_Toc150860436)

[Average specificities of classifiers and frailty scale (Figure A3) 28](#_Toc150860437)

[Average AUCs, sensitivities, and specificities of classifiers by test sizes (Figure A4) 29](#_Toc150860438)

[Performance evaluation of frailty scale (Table A20 & A21) 30](#_Toc150860439)

[Average AUCs of classifiers – test size 0.1 (Table A22) 31](#_Toc150860440)

[Average AUCs of classifiers – test size 0.2 (Table A23) 32](#_Toc150860441)

[Average AUCs of classifiers – test size 0.3 (Table A24) 33](#_Toc150860442)

[Average sensitivities and specificities of classifiers – test size 0.1 (Table A25) 34](#_Toc150860443)

[Average sensitivities and specificities of classifiers – test size 0.2 (Table A26) 35](#_Toc150860444)

[Average sensitivities and specificities of classifiers – test size 0.3 (Table A27) 36](#_Toc150860445)

[Average F1-scores of classifiers – test size 0.1 (Table A28) 37](#_Toc150860446)

[Average F1-scores of classifiers – test size 0.2 (Table A29) 38](#_Toc150860447)

[Average F1-scores of classifiers – test size 0.3 (Table A30) 39](#_Toc150860448)

[Average accuracies and precisions of classifiers – test size 0.1 (Table A31) 40](#_Toc150860449)

[Average accuracies and precisions of classifiers – test size 0.2 (Table A32) 41](#_Toc150860450)

[Average accuracies and precisions of classifiers – test size 0.3 (Table A33) 42](#_Toc150860451)

[Best hyperparameters for each machine learning classification model on imbalanced data set (Table A34) 43](#_Toc150860452)

[6-month mortality prediction on imbalanced data set 44](#_Toc150860453)

[Average AUCs of classifiers and frailty scale (Figure A5) 44](#_Toc150860454)

[Average sensitivities of classifiers and frailty scale (Figure A6) 45](#_Toc150860455)

[Average specificities of classifiers and frailty scale (Figure A7) 46](#_Toc150860456)

[Average AUCs, sensitivities, and specificities of classifiers by test sizes (Figure A8) 47](#_Toc150860457)

[Performance evaluation of frailty scale (Table A35 & A36) 49](#_Toc150860458)

[Average AUCs of classifiers – test size 0.1 (Table A37) 50](#_Toc150860459)

[Average AUCs of classifiers – test size 0.2 (Table A38) 51](#_Toc150860460)

[Average AUCs of classifiers – test size 0.3 (Table A39) 52](#_Toc150860461)

[Average sensitivities of classifiers – test size 0.1 (Table A40) 53](#_Toc150860462)

[Average sensitivities of classifiers – test size 0.2 (Table A41) 54](#_Toc150860463)

[Average sensitivities of classifiers – test size 0.3 (Table A42) 55](#_Toc150860464)

[Average F1-scores of classifiers – test size 0.1 (Table A43) 56](#_Toc150860465)

[Average F1-scores of classifiers – test size 0.2 (Table A44) 57](#_Toc150860466)

[Average F1-scores of classifiers – test size 0.3 (Table A45) 58](#_Toc150860467)

[Average accuracies and precisions of classifiers – test size 0.1 (Table A46) 59](#_Toc150860468)

[Average accuracies and precisions of classifiers – test size 0.2 (Table A47) 60](#_Toc150860469)

[Average accuracies and precisions of classifiers – test size 0.3 (Table A48) 61](#_Toc150860470)

[Average AUCs of classifiers and frailty scale (Figure A9) 62](#_Toc150860471)

[Average sensitivities of classifiers and frailty scale (Figure A10) 63](#_Toc150860472)

[Average specificities of classifiers and frailty scale (Figure A11) 64](#_Toc150860473)

[Average AUCs, sensitivities, and specificities of classifiers by test sizes (Figure A12) 65](#_Toc150860474)

[12-month mortality prediction on imbalanced data set 66](#_Toc150860475)

[Performance evaluation of frailty scale (Table A49 & A50) 66](#_Toc150860476)

[Average AUCs of classifiers – test size 0.1 (Table A51) 67](#_Toc150860477)

[Average AUCs of classifiers – test size 0.2 (Table A52) 68](#_Toc150860478)

[Average AUCs of classifiers – test size 0.3 (Table A53) 69](#_Toc150860479)

[Average sensitivities and specificities of classifiers – test size 0.1 (Table A54) 70](#_Toc150860480)

[Average sensitivities and specificities of classifiers – test size 0.2 (Table A55) 71](#_Toc150860481)

[Average sensitivities and specificities of classifiers – test size 0.3 (Table A56) 72](#_Toc150860482)

[Average F1-scores of classifiers – test size 0.1 (Table A57) 73](#_Toc150860483)

[Average F1-scores of classifiers – test size 0.2 (Table A58) 74](#_Toc150860484)

[Average F1-scores of classifiers – test size 0.3 (Table A59) 75](#_Toc150860485)

[Average accuracies and precisions of classifiers – test size 0.1 (Table A60) 76](#_Toc150860486)

[Average accuracies and precisions of classifiers – test size 0.2 (Table A61) 77](#_Toc150860487)

[Average accuracies and precisions of classifiers – test size 0.3 (Table A62) 78](#_Toc150860488)

# Features utilized for frailty scale development (Table A1)

|  | | **interRAI-HC Questions** | | **Variable Type** | | | **Missing Percentage (%)** | |
| --- | --- | --- | --- | --- | --- | --- | --- | --- |
| Demographic variables: | | | |  | | |  | |
|  | | Age | | Continuous | | | 0.0000 | |
|  | | Gender | | Binrary (Female:1; Male:0) | | | 0.0000 | |
| Clinical Variables: | | | |  | | |  | |
| Cognitive skills for daily decision making | | | |  | | |  | |
|  | | Making decisions regarding tasks of daily life | | Categorical, Ordinal | | | 0.0011 | |
| Memory/Sensitivity ability | | | |  | | |  | |
|  | | Short-term memory | | Binrary, Nominal | | | 0.0631 | |
|  | | Procedural memory | | Binrary, Nominal | | | 0.0610 | |
|  | | Situational memory | | Binrary, Nominal | | | 0.0600 | |
| Periodic disordered thinking of awareness | | | |  | | |  | |
|  | | Easily distracted | | Categorical, Ordinal | | | 0.0621 | |
|  | | Disorganized speech | | Categorical, Ordinal | | | 0.0621 | |
|  | | Mental function varies over day | | Categorical, Ordinal | | | 0.0621 | |
| Acute change in mental stats from person's usual functioning | | | |  | | |  | |
|  | | Acute change in mental status | | Binrary, Nominal | | | 0.0589 | |
| Change in decision making as compared to 90 days ago (or since last assessment) | | | | | | | | |
|  | | Change in decision making | | Categorical, Ordinal | | | 0.0579 | |
| Communication and Vision | | | |  | | |  | |
|  | | Making self understood | | Categorical, Ordinal | | | 0.0547 | |
|  | | Ability to understand others | | Categorical, Ordinal | | | 0.0516 | |
|  | | Hearing | | Categorical, Ordinal | | | 0.0516 | |
|  | | Vision in adequate light | | Categorical, Ordinal | | | 0.0526 | |
| Continence | | | |  | | |  | |
|  | | Bladder continence | | Categorical, Ordinal | | | 0.0021 | |
|  | | Urinary collection device | | Categorical, Ordinal | | | 0.0021 | |
|  | | Bowel continence | | Categorical, Ordinal | | | 0.0021 | |
| Disease diagnosis | | | |  | | |  | |
|  | | Hip fracture | | Categorical, Ordinal | | | 0.0021 | |
|  | | Other fracture | | Categorical, Ordinal | | | 0.0021 | |
|  | | Alzheimer's disease | | Categorical, Ordinal | | | 0.0032 | |
|  | | Dementia other than Alzheimer's disease | | Categorical, Ordinal | | | 0.0021 | |
|  | | Hemiplegia | | Categorical, Ordinal | | | 0.0021 | |
|  | | Multiple sclerosis | | Categorical, Ordinal | | | 0.0021 | |
|  | | Paraplegia | | Categorical, Ordinal | | | 0.0021 | |
|  | | Parkinson's disease | | Categorical, Ordinal | | | 0.0021 | |
|  | | Quadriplegia | | Categorical, Ordinal | | | 0.0021 | |
|  |  | |  | | | *(Continued on next page)* | |  |
|  | | Stroke / CVA | | Categorical, Ordinal | | | 0.0021 | |
|  | | Coronary heart disease | | Categorical, Ordinal | | | 0.0011 | |
|  | | Congestive heart failure | | Categorical, Ordinal | | | 0.0021 | |
|  | | Chronic obstructive pulmonary | | Categorical, Ordinal | | | 0.0021 | |
|  | | Anxiety | | Categorical, Ordinal | | | 0.0032 | |
|  | | Depression | | Categorical, Ordinal | | | 0.0021 | |
|  | | Schizophrenia | | Categorical, Ordinal | | | 0.0021 | |
|  | | Pneumonia | | Categorical, Ordinal | | | 0.0021 | |
|  | | Urinary tract infection | | Categorical, Ordinal | | | 0.0021 | |
|  | | Cancer | | Categorical, Ordinal | | | 0.0021 | |
|  | | Diabetes mellitus | | Categorical, Ordinal | | | 0.0021 | |
|  | | Bipolar disorder | | Categorical, Ordinal | | | 0.0021 | |
| Falls | |  | |  | | |  | |
|  | | Falls | | Categorical, Ordinal | | | 0.0032 | |
|  | | Recent Falls^[[1]](#footnote-2)^ | | Binrary, Nominal | | | 92.5075 | |
| Problem Frequency | | | |  | | |  | |
|  | | Difficulty standing | | Categorical, Ordinal | | | 0.0032 | |
|  | | Difficulty turning around | | Categorical, Ordinal | | | 0.0032 | |
|  | | Dizziness | | Categorical, Ordinal | | | 0.0032 | |
|  | | Unsteady gait | | Categorical, Ordinal | | | 0.0032 | |
|  | | Chest pain | | Categorical, Ordinal | | | 0.0032 | |
|  | | Difficulty clearing airway | | Categorical, Ordinal | | | 0.0032 | |
|  | | Abnormal thought process (0-4) | | Categorical, Ordinal | | | 0.0032 | |
|  | | Delusions (0-4) | | Categorical, Ordinal | | | 0.0032 | |
|  | | Hallucinations (0-4) | | Categorical, Ordinal | | | 0.0042 | |
|  | | Aphasia | | Categorical, Ordinal | | | 0.0042 | |
|  | | Constipation | | Categorical, Ordinal | | | 0.0042 | |
|  | | Diarrhea | | Categorical, Ordinal | | | 0.0042 | |
|  | | Acid reflux | | Categorical, Ordinal | | | 0.0042 | |
|  | | Poor hygiene (0-4) | | Categorical, Ordinal | | | 0.0042 | |
|  | | Vomiting | | Categorical, Ordinal | | | 0.0042 | |
|  | | Difficulty falling asleep | | Categorical, Ordinal | | | 0.0042 | |
|  | | Too much sleep (0-4) | | Categorical, Ordinal | | | 0.0042 | |
|  | | Fever | | Categorical, Ordinal | | | 0.0042 | |
|  | | GI/GU bleeding | | Categorical, Ordinal | | | 0.0042 | |
|  | | Peripheral edema | | Categorical, Ordinal | | | 0.0042 | |
|  | | Aspiration | | Categorical, Ordinal | | | 0.0042 | |
| Dyspnea | | | |  | | |  | |
|  | | Dyspnea | | Categorical, Ordinal | | | 0.0042 | |
| Fatigue | |  | |  | | |  | |
|  | | Inability to complete normal daily activities | | | Categorical, Ordinal | | 0.0042 | |
|  |  | |  | | | *(Continued on next page)* | |  |
| Pain symptoms | | | |  | | |  | |
|  | | Pain frequency | | Categorical, Ordinal | | | 0.0042 | |
|  | | Pain intensity | | Categorical, Ordinal | | | 0.0042 | |
|  | | Pain consistency | | Categorical, Ordinal | | | 0.0042 | |
|  | | Breackthrough pain | | Binrary, Nominal | | | 0.0042 | |
|  | | Pain control | | Categorical, Ordinal | | | 0.0042 | |
| Instability of conditions | | | |  | | |  | |
|  | | Conditions / diseases make cognitive, ADL, mood, or behaviour patterns unstable | | Binrary, Nominal | | | 0.0042 | |
|  | | Experiencing an acute episode, or a flare-up of a recurrent or chronic problem | | Binrary, Nominal | | | 0.0042 | |
|  | | End-stage disease, 6 or fewer months to live | | Binrary, Nominal | | | 0.0042 | |
| self-reported health | | | |  | | |  | |
|  | | Self-rated health | | Categorical, Ordinal | | | 0.0042 | |
| Tobacco and Alcohol | | | |  | | |  | |
|  | | Smokes tobacco daily | | Categorical, Ordinal | | | 0.0042 | |
|  | | Alcohol- Highest number of drinks in any “single sitting” in LAST 14 DAYS | | Categorical, Ordinal | | | 0.0042 | |
| Nutritional Issues | | | |  | | |  | |
|  | | Weight loss | | Binrary, Nominal | | | 0.0042 | |
|  | | Fluid intake | | Binrary, Nominal | | | 0.0042 | |
|  | | Dehydrated | | Binrary, Nominal | | | 0.0042 | |
|  | | One or fewer meals a day | | Binrary, Nominal | | | 0.0042 | |
|  | | Decrease in food or fluid | | Binrary, Nominal | | | 0.0042 | |
|  | | Fluid output exceeds input | | Binrary, Nominal | | | 0.0042 | |
|  | | Mode of nutritional intake | | Categorical, Ordinal | | | 0.0042 | |
| Dental or oral | | | |  | | |  | |
|  | | Dentures | | Binrary, Nominal | | | 0.0042 | |
|  | | Broken teeth | | Binrary, Nominal | | | 0.0042 | |
|  | | Difficulty chewing | | Binrary, Nominal | | | 0.0053 | |
|  | | Dry mouth | | Binrary, Nominal | | | 0.0053 | |
| Skin conditions | | | |  | | |  | |
|  | | Most severe pressure ulcer | | Categorical, Ordinal | | | 0.0053 | |
|  | | Prior pressure ulcer | | Binrary, Nominal | | | 0.0053 | |
|  | | Other skin ulcer | | Binrary, Nominal | | | 0.0053 | |
|  | | Major skin problems | | Binrary, Nominal | | | 0.0053 | |
|  | | Skin tears | | Binrary, Nominal | | | 0.0053 | |
|  | | Other skin condition | | Binrary, Nominal | | | 0.0053 | |
|  | | Foot problems | | Categorical, Ordinal | | | 0.0063 | |
|  |  | |  | | | *(Continued on next page)* | |  |
| IADL capacity | | | |  | | |  | |
|  | | Meal preparation | | Categorical, Ordinal | | | 0.0011 | |
|  | | Housework | | Categorical, Ordinal | | | 0.0011 | |
|  | | Finance | | Categorical, Ordinal | | | 0.0011 | |
|  | | Managing medications | | Categorical, Ordinal | | | 0.0011 | |
|  | | Phone use | | Categorical, Ordinal | | | 0.0011 | |
|  | | Stairs | | Categorical, Ordinal | | | 0.0011 | |
|  | | Shopping | | Categorical, Ordinal | | | 0.0011 | |
|  | | Transportation | | Categorical, Ordinal | | | 0.0011 | |
| ADL self performance | | | |  | | |  | |
|  | | Bathing | | Categorical, Ordinal | | | 0.0011 | |
|  | | Personal Hygiene | | Categorical, Ordinal | | | 0.0011 | |
|  | | Dressing upper body | | Categorical, Ordinal | | | 0.0011 | |
|  | | Dressing lower body | | Categorical, Ordinal | | | 0.0011 | |
|  | | Walking | | Categorical, Ordinal | | | 0.0011 | |
|  | | Locomotion | | Categorical, Ordinal | | | 0.0011 | |
|  | | Transfer toilet | | Categorical, Ordinal | | | 0.0011 | |
|  | | Toilet use | | Categorical, Ordinal | | | 0.0011 | |
|  | | Bed mobility | | Categorical, Ordinal | | | 0.0011 | |
|  | | Eating | | Categorical, Ordinal | | | 0.0011 | |
|  | | Change in ADL Status (0-2,8) | | Categorical, Ordinal | | | 0.0011 | |
| Psychosocial Variables: | | | |  | | |  | |
| Indicators of possible depressed, anxious, or sad mood | | | |  | | |  | |
|  | | Indicators of depression - Made negative statements | | Categorical, Ordinal | | | 0.0558 | |
|  | | Indicators of depression - Persistant anger with self or others | | Categorical, Ordinal | | | 0.0558 | |
|  | | Indicators of depression - Expressions, including nonverbal, of what appear to be unrealistic fears | | Categorical, Ordinal | | | 0.0558 | |
|  | | Indicators of depression - Repetitive health complaints | | Categorical, Ordinal | | | 0.0568 | |
|  | | Indicators of depression - Repetitive anxious complaints | | Categorical, Ordinal | | | 0.0568 | |
|  | | Indicators of depression - Sad, pained, or worried facial expressions | | Categorical, Ordinal | | | 0.0568 | |
|  | | Indicators of depression - Crying, tearfulness | | Categorical, Ordinal | | | 0.0568 | |
|  | | Indicators of depression - Recurrent statements that something terrible is about to happen | | Categorical, Ordinal | | | 0.0547 | |
|  |  | |  | | | *(Continued on next page)* | |  |
|  | | Indicators of depression - Withdrawal from activities of interest | | Categorical, Ordinal | | | 0.0558 | |
|  | | Indicators of depression - Reduced social interactions | | Categorical, Ordinal | | | 0.0558 | |
|  | | Indicators of depression - Expressions, including nonverbal, of a lack of pleasure in life | | Categorical, Ordinal | | | 0.0568 | |
| Self-reported mood | | | |  | | |  | |
|  | | Little interest or pleasure in things you normally enjoy | | Categorical, Ordinal | | | 0.0568 | |
|  | | Anxious, restless, or uneasy | | Categorical, Ordinal | | | 0.0568 | |
|  | | Sad, depressed or hopeless | | Categorical, Ordinal | | | 0.0568 | |
| Behavior symptoms | | | |  | | |  | |
|  | | Behaviour symptoms: Wandering | | Categorical, Ordinal | | | 0.0579 | |
|  | | Behaviour symptoms: Verbal abuse | | Categorical, Ordinal | | | 0.0579 | |
|  | | Behaviour symptoms: Physical abuse | | Categorical, Ordinal | | | 0.0568 | |
|  | | Behaviour symptoms: Socially inappropriate or disruptive behaviour | | Categorical, Ordinal | | | 0.0579 | |
|  | | Behaviour symptoms: Resists care | | Categorical, Ordinal | | | 0.0579 | |
|  | | Behaviour symptoms: Inappropriate sexual behavior | | Categorical, Ordinal | | | 0.0568 | |
| Social relationships | | | |  | | |  | |
|  | | Social relationships - Participation in social activities | | Categorical, Ordinal | | | 0.0568 | |
|  | | Social relationships - Visit with a long-standing social relation or family member | | Categorical, Ordinal | | | 0.0568 | |
|  | | Social relationships - Other interaction with long-standing social relation or family member | | Categorical, Ordinal | | | 0.0568 | |
|  | | Social relationships - Says or indicates that he / she feels lonely | | Binrary, Nominal | | | 0.0547 | |
|  | | Major life stressors in last 90 days | | Binrary, Nominal | | | 0.0558 | |
|  | | Unsettled relationships - Family or close friends report feeling overwhelmed by person’s support needs | | Binrary, Nominal | | | 4.4997 | |
|  | | Strengths - Strong and supportive relationship with family | | Binrary, Nominal | | | 0.0084 | |

# Features utilized for interRAI Home Care Frailty Scale (Table A2)

| **interRAI Home Care Frailty Scale Item** | **interRAI-HC Questions** |
| --- | --- |
| **Function** |  |
| IADL – Housework | Housework |
| IADL – Meals | Meal preparation performance |
| IADL – Meals | Meal preparation |
| IADL – Phone Use | Phone use |
| ADL – Personal Hygiene | Personal Hygiene |
| ADL – Locomotion | Locomotion |
| ADL - Transfer | Transfer toilet |
| ADL – Toilet Use | Toilet use |
| **Movement or Movement Related** |  |
| Climb Stairs | Stairs |
| Hrs of Phy Activity | Physical Activities Promotion |
| Fell in Last 90 Days | Falls |
| Dizzy | Dizziness |
| **Cognition and Communication** |  |
| Cog – Decision Making | Making decisions regarding tasks of daily life |
| IADL - Manage Medication | Managing medications |
| IADL – Manage Finances | Finance |
| Dementia Other Than Alzhimers | Dementia other than Alzheimer's disease |
| Understand Others | Ability to understand others |
| **Social** |  |
| Decline in Soc Act | Indicators of depression - Reduced social interactions |
| Reduced Soc Act | Indicators of depression - Reduced social interactions |
| Withdrawal From Activities of Interest | Indicators of depression - Withdrawal from activities of interest |
| **Nutritional Status** |  |
| Weight Loss | Weight loss |
| Loss of Appetite | One or fewer meals a day |
| Decrease in Food Eaten | Decrease in food or fluid |
| **Clinical Symptoms and Diagnoses** |  |
| Bowel Incontinent | Bowel continence |
| Urinary Tract Infect | Urinary collection device |
| Pneumonia | Pneumonia |
| Conges Heart Fail | Congestive heart failure |

# Best hyperparameters for each machine learning classification model on balanced data set (Table A3)

| **Hyperparameter** | **6-month Mortality** | **12-month Mortality** |
| --- | --- | --- |
| **Logistic Regression** |  |  |
| solver | liblinear | liblinear |
| penalty | L1 | L2 |
| c values | 0.1 | 100 |
| **RF** |  |  |
| n estimator | 500 | 500 |
| min sample split | 5 | 5 |
| min sample leaf | 1 | 1 |
| max features | auto | auto |
| max depth | 40 | 40 |
| bootstrap | FALSE | FALSE |
| **XGBoost** |  |  |
| n estimator | 2000 | 2000 |
| eta | 0.01 | 0.01 |
| max depth | 8 | 8 |
| min child weight | 5 | 8 |
| nthread | 4 | 4 |
| **MLP** |  |  |
| activation (Dense1) | relu | relu |
| activation (Output) | sigmoid | sigmoid |
| hidden layers | 1 | 1 |
| dropout | 0.15 | 0.25 |
| learning rate | 0.001 | 0.001 |
| neurons per layer | 512 | 512 |
| batch size | 128 | 128 |
| optimizer | adam | adam |

# 6-month mortality prediction on balanced data set

## Sensitivity Analysis on Imputed and Unimputed data sets (Table A4)

**Table A4:** **Sensitivity Analysis on Imputed and Unimputed data sets for 6-month mortality prediction on balanced data set (Highest values of each condition are highlighted in bold)**

| **Model** | **Sample Size** | **Number of Features** | **Data Imputation** | **ROC_AUC (SD)** | **Sensitivity (SD)** | **Specificity （SD）** |
| --- | --- | --- | --- | --- | --- | --- |
| **XGBoost** | **4141** | **138** | **Imputed** | **0.764 (0.034)** | **0.280 (0.059)** | 0.949 (0.011) |
|  |  |  | **Unimputed** | 0.697 (0.037) | 0.141 (0.043) | **0.962 (0.011)** |
|  | **1000** | **138** | **Imputed** | **0.705 (0.063)** | **0.236 (0.101)** | 0.915 (0.024) |
|  |  |  | **Unimputed** | 0.690 (0.074) | 0.135 (0.092) | **0.956 (0.022)** |
|  | **4141** | **10** | **Imputed** | **0.621 (0.046)** | **0.450 (0.104)** | **0.730 (0.065)** |
|  |  |  | **Unimputed** | 0.577 (0.047) | 0.398 (0.100) | 0.716 (0.065) |
|  | **1000** | **10** | **Imputed** | 0.587 (0.073) | 0.325 (0.112) | **0.780 (0.079)** |
|  |  |  | **Unimputed** | **0.608 (0.101)** | **0.561 (0.190)** | 0.602 (0.111) |
| **RLR** | **4141** | **138** | **Imputed** | **0.777 (0.033)** | **0.659 (0.073)** | **0.748 (0.026)** |
|  |  |  | **Unimputed** | 0.725 (0.037) | 0.615 (0.066) | 0.700 (0.026) |
|  | **1000** | **138** | **Imputed** | **0.722 (0.086)** | **0.621 (0.162)** | **0.730 (0.047)** |
|  |  |  | **Unimputed** | 0.692 (0.074) | 0.531 (0.143) | 0.717 (0.046) |
|  | **4141** | **10** | **Imputed** | **0.678 (0.050)** | **0.627 (0.107)** | **0.648 (0.079)** |
|  |  |  | **Unimputed** | 0.642 (0.055) | 0.594 (0.112) | 0.618 (0.077) |
|  | **1000** | **10** | **Imputed** | **0.651 (0.084)** | **0.597 (0.136)** | **0.627 (0.115)** |
|  |  |  | **Unimputed** | 0.608 (0.101) | 0.561 (0.190) | 0.602 (0.111) |
| **RF** | **4141** | **138** | **Imputed** | **0.771 (0.032)** | **0.108 (0.042)** | 0.989 (0.006) |
|  |  |  | **Unimputed** | 0.713 (0.037) | 0.013 (0.015) | **0.998 (0.003)** |
|  | **1000** | **138** | **Imputed** | **0.717 (0.075)** | **0.064 (0.067)** | 0.980 (0.014) |
|  |  |  | **Unimputed** | 0.692 (0.080) | 0.008 (0.031) | **0.998 (0.004)** |
|  | **4141** | **10** | **Imputed** | **0.586 (0.047)** | **0.364 (0.121)** | **0.767 (0.077)** |
|  |  |  | **Unimputed** | 0.553 (0.048) | 0.330 (0.123) | 0.747 (0.087) |
|  | **1000** | **10** | **Imputed** | **0.577 (0.100)** | **0.256 (0.174)** | **0.826 (0.068)** |
|  |  |  | **Unimputed** | 0.539 (0.107) | 0.253 (0.144) | 0.794 (0.078) |
| **MLP** | **4141** | **138** | **Imputed** | **0.763 (0.035)** | **0.665 (0.083)** | **0.711 (0.051)** |
|  |  |  | **Unimputed** | 0.702 (0.043) | 0.592 (0.103) | 0.696 (0.059) |
|  | **1000** | **138** | **Imputed** | **0.723 (0.073)** | 0.579 (0.169) | **0.718 (0.051)** |
|  |  |  | **Unimputed** | 0.692 (0.076) | **0.581 (0.145)** | 0.717 (0.081) |
|  | **4141** | **10** | **Imputed** | **0.676 (0.050)** | **0.624 (0.109)** | **0.639 (0.077)** |
|  |  |  | **Unimputed** | 0.638 (0.055) | 0.597 (0.114) | 0.605 (0.077) |
|  | **1000** | **10** | **Imputed** | **0.645 (0.091)** | **0.565 (0.142)** | **0.635 (0.092)** |
|  |  |  | **Unimputed** | 0.604 (0.107) | 0.555 (0.185) | 0.598 (0.090) |

RLR=Regularized Logistic Regression. RF=Random Forest. MLP=Multilayer Perceptron. XGBoost=eXtreme Gradient Boosting. SD=Standard Deviation

## Performance evaluation of frailty scale (Table A5 & A6)

**Table A5:**

**Average AUCs, sensitivities, and specificities of frailty scale for 6-month mortality prediction on balanced data set**

| **Sample Size** | **interRAI Home Care Frailty** | | |
| --- | --- | --- | --- |
|  | **Average AUC (SD)** | **Average Sensitivity (SD)** | **Average Specificity (SD)** |
| **1000** | 0.698 (0.028) | 0.660 (0.034) | 0.632 (0.029) |
| **4000** | 0.674 (0.008) | 0.621 (0.007) | 0.632 (0.004) |
| **16000** | 0.679 (0.002) | 0.622 (0.002) | 0.637 (0.002) |
| **95042** | 0.681 (0.000) | 0.628 (0.001) | 0.635 (0.000) |

SD=Standard Deviation

**Table A6:**

**Average accuracies, precisions, and F1-scores of frailty scale for 6-month mortality prediction on balanced data set**

| **Sample Size** | **interRAI Home Care Frailty** | | |
| --- | --- | --- | --- |
|  | **Average Accuracy (SD)** | **Average Precision (SD)** | **Average F1-score (SD)** |
| **1000** | 0.646 (0.031) | 0.642 (0.029) | 0.651 (0.031) |
| **4000** | 0.626 (0.005) | 0.628 (0.004) | 0.624 (0.006) |
| **16000** | 0.629 (0.002) | 0.631 (0.002) | 0.627 (0.002) |
| **95042** | 0.632 (0.000) | 0.633 (0.000) | 0.630 (0.001) |

SD=Standard Deviation

## Average AUCs of classifiers - test size 0.1(Table A7)

**Table A7: Average AUCs of classifiers for 6-month mortality prediction on balanced data set (test size 0.1)**

| **Test Size** | **Sample**  **Size** | **Number of features** | **Average AUC (SD)** | | | |
| --- | --- | --- | --- | --- | --- | --- |
|  |  |  | **RLR** | **RF** | **MLP** | **XGBoost** |
| **0.1** | **95042** | **138** | 0.792 (0.006) | 0.790 (0.007) | 0.787 (0.008) | 0.796 (0.006) |
|  |  | **80** | 0.775 (0.007) | 0.773 (0.010) | 0.776 (0.011) | 0.776 (0.009) |
|  |  | **40** | 0.753 (0.013) | 0.736 (0.017) | 0.758 (0.013) | 0.747 (0.014) |
|  |  | **30** | 0.745 (0.014) | 0.701 (0.020) | 0.746 (0.014) | 0.733 (0.014) |
|  |  | **20** | 0.724 (0.022) | 0.642 (0.035) | 0.731 (0.024) | 0.716 (0.021) |
|  |  | **10** | 0.679 (0.034) | 0.628 (0.031) | 0.680 (0.033) | 0.667 (0.032) |
|  | **16000** | **138** | 0.789 (0.013) | 0.784 (0.014) | 0.776 (0.019) | 0.773 (0.018) |
|  |  | **80** | 0.772 (0.018) | 0.767 (0.019) | 0.766 (0.017) | 0.750 (0.014) |
|  |  | **40** | 0.756 (0.023) | 0.735 (0.031) | 0.755 (0.025) | 0.720 (0.024) |
|  |  | **30** | 0.734 (0.014) | 0.692 (0.023) | 0.731 (0.016) | 0.685 (0.022) |
|  |  | **20** | 0.724 (0.035) | 0.647 (0.043) | 0.726 (0.035) | 0.669 (0.035) |
|  |  | **10** | 0.686 (0.037) | 0.604 (0.039) | 0.683 (0.036) | 0.644 (0.039) |
|  | **4000** | **138** | 0.772 (0.031) | 0.769 (0.028) | 0.762 (0.026) | 0.769 (0.031) |
|  |  | **80** | 0.771 (0.048) | 0.768 (0.035) | 0.764 (0.044) | 0.753 (0.036) |
|  |  | **40** | 0.739 (0.039) | 0.724 (0.033) | 0.735 (0.040) | 0.698 (0.033) |
|  |  | **30** | 0.733 (0.050) | 0.705 (0.047) | 0.732 (0.051) | 0.688 (0.037) |
|  |  | **20** | 0.722 (0.036) | 0.656 (0.036) | 0.718 (0.037) | 0.636 (0.034) |
|  |  | **10** | 0.677 (0.065) | 0.591 (0.067) | 0.675 (0.062) | 0.621 (0.068) |
|  | **1000** | **138** | 0.722 (0.086) | 0.717 (0.075) | 0.723 (0.073) | 0.705 (0.063) |
|  |  | **80** | 0.714 (0.061) | 0.693 (0.072) | 0.708 (0.071) | 0.655 (0.065) |
|  |  | **40** | 0.716 (0.065) | 0.698 (0.070) | 0.692 (0.064) | 0.678 (0.080) |
|  |  | **30** | 0.704 (0.076) | 0.646 (0.075) | 0.688 (0.068) | 0.602 (0.072) |
|  |  | **20** | 0.683 (0.077) | 0.637 (0.068) | 0.667 (0.079) | 0.607 (0.069) |
|  |  | **10** | 0.651 (0.084) | 0.577 (0.100) | 0.645 (0.091) | 0.587 (0.073) |

RLR=Regularized Logistic Regression. RF=Random Forest. MLP=Multilayer Perceptron. XGBoost=eXtreme Gradient Boosting. SD=Standard Deviation

## Average AUCs of classifiers - test size 0.2 (Table A8)

**Table A8: Average AUCs of classifiers of or 6-month mortality prediction on balanced data set (test size 0.2)**

| **Test Size** | **Sample Size** | **Number of features** | **Average AUC (SD)** | | | |
| --- | --- | --- | --- | --- | --- | --- |
|  |  |  | **RLR** | **RF** | **MLP** | **XGBoost** |
| **0.2** | **95042** | **138** | 0.790 (0.003) | 0.788 (0.005) | 0.787 (0.006) | 0.794 (0.004) |
|  |  | **80** | 0.774 (0.011) | 0.770 (0.013) | 0.774 (0.014) | 0.772 (0.012) |
|  |  | **40** | 0.753 (0.013) | 0.733 (0.018) | 0.756 (0.012) | 0.744 (0.013) |
|  |  | **30** | 0.731 (0.017) | 0.689 (0.028) | 0.736 (0.017) | 0.719 (0.018) |
|  |  | **20** | 0.725 (0.020) | 0.648 (0.035) | 0.727 (0.020) | 0.709 (0.020) |
|  |  | **10** | 0.696 (0.030) | 0.628 (0.027) | 0.696 (0.028) | 0.679 (0.029) |
|  | **16000** | **138** | 0.785 (0.011) | 0.777 (0.011) | 0.774 (0.014) | 0.766 (0.011) |
|  |  | **80** | 0.772 (0.013) | 0.761 (0.013) | 0.764 (0.014) | 0.747 (0.015) |
|  |  | **40** | 0.757 (0.019) | 0.733 (0.021) | 0.752 (0.019) | 0.715 (0.024) |
|  |  | **30** | 0.740 (0.018) | 0.702 (0.027) | 0.740 (0.021) | 0.691 (0.028) |
|  |  | **20** | 0.733 (0.013) | 0.650 (0.023) | 0.730 (0.016) | 0.672 (0.021) |
|  |  | **10** | 0.684 (0.039) | 0.595 (0.041) | 0.686 (0.041) | 0.636 (0.051) |
|  | **4000** | **138** | 0.769 (0.021) | 0.768 (0.017) | 0.774 (0.017) | 0.757 (0.015) |
|  |  | **80** | 0.767 (0.024) | 0.765 (0.023) | 0.769 (0.023) | 0.744 (0.028) |
|  |  | **40** | 0.741 (0.030) | 0.733 (0.027) | 0.742 (0.029) | 0.713 (0.029) |
|  |  | **30** | 0.735 (0.024) | 0.711 (0.035) | 0.732 (0.028) | 0.683 (0.029) |
|  |  | **20** | 0.721 (0.029) | 0.642 (0.049) | 0.717 (0.033) | 0.646 (0.037) |
|  |  | **10** | 0.677 (0.045) | 0.572 (0.043) | 0.673 (0.047) | 0.601 (0.036) |
|  | **1000** | **138** | 0.747 (0.042) | 0.760 (0.043) | 0.751 (0.048) | 0.752 (0.051) |
|  |  | **80** | 0.750 (0.047) | 0.752 (0.054) | 0.747 (0.051) | 0.738 (0.057) |
|  |  | **40** | 0.721 (0.055) | 0.715 (0.072) | 0.723 (0.059) | 0.696 (0.063) |
|  |  | **30** | 0.705 (0.067) | 0.679 (0.067) | 0.688 (0.068) | 0.651 (0.062) |
|  |  | **20** | 0.724 (0.051) | 0.674 (0.061) | 0.716 (0.052) | 0.661 (0.054) |
|  |  | **10** | 0.671 (0.072) | 0.573 (0.077) | 0.674 (0.079) | 0.605 (0.070) |

RLR=Regularized Logistic Regression. RF=Random Forest. MLP=Multilayer Perceptron. XGBoost=eXtreme Gradient Boosting. SD=Standard Deviation

## Average AUCs of classifiers - test size 0.3 (Table A9)

**Table A9: Average AUCs of classifiers f or 6-month mortality prediction on balanced data set (test size 0.3)**

| **Test Size** | **Sample**  **Size** | **Number of features** | **Average AUC (SD)** | | | |
| --- | --- | --- | --- | --- | --- | --- |
|  |  |  | **RLR** | **RF** | **MLP** | **XGBoost** |
| **0.3** | **95042** | **138** | 0.788 (0.003) | 0.786 (0.004) | 0.779 (0.005) | 0.791 (0.003) |
|  |  | **80** | 0.773 (0.007) | 0.770 (0.008) | 0.772 (0.009) | 0.772 (0.009) |
|  |  | **40** | 0.755 (0.011) | 0.734 (0.016) | 0.756 (0.013) | 0.743 (0.014) |
|  |  | **30** | 0.741 (0.010) | 0.697 (0.024) | 0.746 (0.012) | 0.727 (0.012) |
|  |  | **20** | 0.715 (0.031) | 0.628 (0.032) | 0.718 (0.030) | 0.697 (0.030) |
|  |  | **10** | 0.681 (0.043) | 0.616 (0.034) | 0.684 (0.041) | 0.663 (0.041) |
|  | **16000** | **138** | 0.789 (0.009) | 0.787 (0.007) | 0.781 (0.011) | 0.782 (0.007) |
|  |  | **80** | 0.774 (0.008) | 0.769 (0.010) | 0.767 (0.014) | 0.755 (0.013) |
|  |  | **40** | 0.748 (0.018) | 0.728 (0.019) | 0.748 (0.017) | 0.712 (0.019) |
|  |  | **30** | 0.743 (0.015) | 0.710 (0.023) | 0.740 (0.018) | 0.696 (0.020) |
|  |  | **20** | 0.729 (0.021) | 0.662 (0.035) | 0.730 (0.024) | 0.681 (0.024) |
|  |  | **10** | 0.685 (0.034) | 0.606 (0.042) | 0.691 (0.039) | 0.644 (0.040) |
|  | **4000** | **138** | 0.761 (0.021) | 0.762 (0.014) | 0.753 (0.018) | 0.756 (0.019) |
|  |  | **80** | 0.744 (0.021) | 0.744 (0.023) | 0.738 (0.020) | 0.727 (0.021) |
|  |  | **40** | 0.725 (0.014) | 0.708 (0.024) | 0.718 (0.018) | 0.682 (0.026) |
|  |  | **30** | 0.725 (0.024) | 0.687 (0.024) | 0.717 (0.023) | 0.667 (0.026) |
|  |  | **20** | 0.699 (0.023) | 0.638 (0.038) | 0.694 (0.024) | 0.635 (0.032) |
|  |  | **10** | 0.675 (0.036) | 0.567 (0.034) | 0.669 (0.039) | 0.605 (0.034) |
|  | **1000** | **138** | 0.752 (0.034) | 0.750 (0.032) | 0.720 (0.038) | 0.743 (0.037) |
|  |  | **80** | 0.740 (0.027) | 0.725 (0.039) | 0.715 (0.039) | 0.709 (0.026) |
|  |  | **40** | 0.724 (0.038) | 0.701 (0.039) | 0.709 (0.035) | 0.685 (0.048) |
|  |  | **30** | 0.729 (0.045) | 0.709 (0.041) | 0.716 (0.054) | 0.684 (0.047) |
|  |  | **20** | 0.710 (0.036) | 0.657 (0.050) | 0.705 (0.036) | 0.645 (0.050) |
|  |  | **10** | 0.639 (0.089) | 0.577 (0.056) | 0.639 (0.080) | 0.581 (0.072) |

RLR=Regularized Logistic Regression. RF=Random Forest. MLP=Multilayer Perceptron. XGBoost=eXtreme Gradient Boosting. SD=Standard Deviatio

## Average sensitivities and specificities of classifiers - test size 0.1 (Table A10)

**Table A10: Average sensitivities and specificities of classifiers for 6-month mortality prediction on balanced data set (test size 0.1)**

| **Test Size** | **Sample**  **Size** | **Number of features** | **Average Sensitivity (SD)** | | | | **Average Specificity (SD)** | | | |
| --- | --- | --- | --- | --- | --- | --- | --- | --- | --- | --- |
|  |  |  | **RLR** | **RF** | **MLP** | **XGBoost** | **RLR** | **RF** | **MLP** | **XGBoost** |
| **0.1** | **95042** | **138** | 0.701 (0.015) | 0.147 (0.008) | 0.755 (0.039) | 0.569 (0.012) | 0.739 (0.004) | 0.980 (0.002) | 0.676 (0.036) | 0.841 (0.004) |
|  |  | **80** | 0.684 (0.015) | 0.138 (0.023) | 0.724 (0.038) | 0.565 (0.019) | 0.728 (0.012) | 0.979 (0.003) | 0.689 (0.033) | 0.823 (0.006) |
|  |  | **40** | 0.678 (0.023) | 0.127 (0.035) | 0.680 (0.033) | 0.571 (0.020) | 0.702 (0.019) | 0.972 (0.006) | 0.702 (0.033) | 0.784 (0.013) |
|  |  | **30** | 0.670 (0.025) | 0.151 (0.045) | 0.646 (0.051) | 0.571 (0.027) | 0.698 (0.022) | 0.950 (0.018) | 0.715 (0.046) | 0.763 (0.013) |
|  |  | **20** | 0.653 (0.029) | 0.219 (0.062) | 0.647 (0.046) | 0.581 (0.027) | 0.680 (0.025) | 0.888 (0.036) | 0.692 (0.047) | 0.735 (0.014) |
|  |  | **10** | 0.620 (0.084) | 0.478 (0.174) | 0.583 (0.075) | 0.595 (0.072) | 0.646 (0.062) | 0.706 (0.105) | 0.677 (0.063) | 0.654 (0.055) |
|  | **16000** | **138** | 0.692 (0.036) | 0.140 (0.021) | 0.717 (0.066) | 0.355 (0.032) | 0.746 (0.010) | 0.981 (0.005) | 0.700 (0.045) | 0.921 (0.008) |
|  |  | **80** | 0.674 (0.030) | 0.123 (0.033) | 0.718 (0.045) | 0.347 (0.042) | 0.732 (0.016) | 0.981 (0.003) | 0.684 (0.040) | 0.911 (0.005) |
|  |  | **40** | 0.679 (0.041) | 0.134 (0.046) | 0.733 (0.052) | 0.400 (0.051) | 0.710 (0.020) | 0.975 (0.010) | 0.647 (0.035) | 0.868 (0.024) |
|  |  | **30** | 0.650 (0.034) | 0.117 (0.039) | 0.682 (0.064) | 0.382 (0.039) | 0.699 (0.028) | 0.960 (0.013) | 0.662 (0.055) | 0.842 (0.021) |
|  |  | **20** | 0.646 (0.066) | 0.187 (0.071) | 0.651 (0.073) | 0.421 (0.070) | 0.687 (0.052) | 0.901 (0.042) | 0.686 (0.051) | 0.804 (0.029) |
|  |  | **10** | 0.610 (0.088) | 0.467 (0.084) | 0.600 (0.079) | 0.528 (0.070) | 0.662 (0.079) | 0.713 (0.050) | 0.665 (0.082) | 0.694 (0.049) |
|  | **4000** | **138** | 0.647 (0.065) | 0.100 (0.032) | 0.640 (0.052) | 0.330 (0.057) | 0.749 (0.026) | 0.982 (0.007) | 0.743 (0.047) | 0.938 (0.016) |
|  |  | **80** | 0.673 (0.077) | 0.119 (0.050) | 0.678 (0.093) | 0.316 (0.058) | 0.739 (0.027) | 0.981 (0.007) | 0.712 (0.042) | 0.930 (0.012) |
|  |  | **40** | 0.664 (0.073) | 0.106 (0.048) | 0.708 (0.062) | 0.281 (0.059) | 0.700 (0.034) | 0.973 (0.009) | 0.642 (0.070) | 0.902 (0.018) |
|  |  | **30** | 0.623 (0.093) | 0.150 (0.064) | 0.662 (0.081) | 0.334 (0.072) | 0.707 (0.038) | 0.962 (0.013) | 0.688 (0.041) | 0.880 (0.024) |
|  |  | **20** | 0.618 (0.064) | 0.165 (0.050) | 0.640 (0.081) | 0.308 (0.083) | 0.705 (0.037) | 0.923 (0.027) | 0.677 (0.051) | 0.836 (0.028) |
|  |  | **10** | 0.602 (0.089) | 0.405 (0.141) | 0.615 (0.086) | 0.469 (0.105) | 0.653 (0.072) | 0.730 (0.093) | 0.640 (0.083) | 0.714 (0.077) |
|  | **1000** | **138** | 0.621 (0.162) | 0.064 (0.067) | 0.579 (0.169) | 0.236 (0.101) | 0.730 (0.047) | 0.980 (0.014) | 0.718 (0.051) | 0.915 (0.024) |
|  |  | **80** | 0.633 (0.113) | 0.062 (0.074) | 0.636 (0.116) | 0.223 (0.123) | 0.692 (0.051) | 0.984 (0.017) | 0.692 (0.064) | 0.907 (0.024) |
|  |  | **40** | 0.661 (0.132) | 0.114 (0.054) | 0.617 (0.123) | 0.298 (0.117) | 0.690 (0.051) | 0.962 (0.025) | 0.667 (0.061) | 0.884 (0.036) |
|  |  | **30** | 0.650 (0.142) | 0.100 (0.085) | 0.625 (0.135) | 0.213 (0.118) | 0.647 (0.076) | 0.956 (0.021) | 0.641 (0.073) | 0.871 (0.039) |
|  |  | **20** | 0.616 (0.115) | 0.137 (0.086) | 0.602 (0.143) | 0.284 (0.079) | 0.662 (0.070) | 0.920 (0.041) | 0.644 (0.081) | 0.840 (0.051) |
|  |  | **10** | 0.597 (0.136) | 0.256 (0.174) | 0.565 (0.142) | 0.325 (0.112) | 0.627 (0.115) | 0.826 (0.068) | 0.635 (0.092) | 0.780 (0.079) |

## Average sensitivities and specificities of classifiers - test size 0.2 (Table A11)

**Table A11: Average sensitivities and specificities of classifiers for 6-month mortality prediction on balanced data set (test size 0.2)**

| **Test Size** | **Sample**  **Size** | **Number of features** | **Average Sensitivity (SD)** | | | | **Average Specificity (SD)** | | | |
| --- | --- | --- | --- | --- | --- | --- | --- | --- | --- | --- |
|  |  |  | **RLR** | **RF** | **MLP** | **XGBoost** | **RLR** | **RF** | **MLP** | **XGBoost** |
| **0.2** | **95042** | **138** | 0.697 (0.005) | 0.143 (0.005) | 0.761 (0.044) | 0.557 (0.011) | 0.740 (0.004) | 0.980 (0.001) | 0.665 (0.045) | 0.847 (0.004) |
|  |  | **80** | 0.686 (0.017) | 0.133 (0.030) | 0.732 (0.044) | 0.550 (0.017) | 0.725 (0.013) | 0.980 (0.004) | 0.677 (0.038) | 0.826 (0.009) |
|  |  | **40** | 0.676 (0.019) | 0.129 (0.033) | 0.683 (0.038) | 0.561 (0.018) | 0.703 (0.018) | 0.972 (0.007) | 0.697 (0.044) | 0.789 (0.012) |
|  |  | **30** | 0.653 (0.026) | 0.137 (0.041) | 0.641 (0.051) | 0.549 (0.031) | 0.692 (0.023) | 0.948 (0.023) | 0.708 (0.039) | 0.767 (0.019) |
|  |  | **20** | 0.654 (0.031) | 0.205 (0.071) | 0.628 (0.060) | 0.569 (0.029) | 0.682 (0.027) | 0.897 (0.046) | 0.703 (0.054) | 0.737 (0.020) |
|  |  | **10** | 0.628 (0.050) | 0.509 (0.056) | 0.594 (0.105) | 0.589 (0.041) | 0.665 (0.045) | 0.707 (0.038) | 0.689 (0.074) | 0.682 (0.026) |
|  | **16000** | **138** | 0.680 (0.021) | 0.115 (0.015) | 0.714 (0.064) | 0.323 (0.019) | 0.740 (0.007) | 0.985 (0.002) | 0.694 (0.046) | 0.928 (0.005) |
|  |  | **80** | 0.673 (0.030) | 0.102 (0.024) | 0.715 (0.042) | 0.329 (0.031) | 0.732 (0.016) | 0.984 (0.004) | 0.674 (0.035) | 0.914 (0.008) |
|  |  | **40** | 0.670 (0.036) | 0.121 (0.043) | 0.711 (0.051) | 0.369 (0.031) | 0.712 (0.027) | 0.974 (0.006) | 0.666 (0.043) | 0.876 (0.019) |
|  |  | **30** | 0.653 (0.024) | 0.122 (0.041) | 0.704 (0.058) | 0.374 (0.058) | 0.703 (0.024) | 0.961 (0.011) | 0.654 (0.040) | 0.852 (0.019) |
|  |  | **20** | 0.670 (0.032) | 0.172 (0.059) | 0.698 (0.058) | 0.422 (0.048) | 0.678 (0.030) | 0.907 (0.038) | 0.646 (0.046) | 0.799 (0.027) |
|  |  | **10** | 0.622 (0.073) | 0.411 (0.125) | 0.593 (0.105) | 0.496 (0.088) | 0.651 (0.071) | 0.742 (0.081) | 0.669 (0.064) | 0.713 (0.059) |
|  | **4000** | **138** | 0.642 (0.040) | 0.116 (0.022) | 0.674 (0.056) | 0.303 (0.040) | 0.766 (0.011) | 0.980 (0.006) | 0.733 (0.043) | 0.939 (0.010) |
|  |  | **80** | 0.653 (0.053) | 0.101 (0.029) | 0.690 (0.076) | 0.281 (0.038) | 0.750 (0.026) | 0.983 (0.006) | 0.716 (0.052) | 0.937 (0.009) |
|  |  | **40** | 0.647 (0.050) | 0.122 (0.041) | 0.676 (0.069) | 0.304 (0.053) | 0.721 (0.027) | 0.971 (0.008) | 0.686 (0.044) | 0.911 (0.012) |
|  |  | **30** | 0.644 (0.037) | 0.125 (0.052) | 0.646 (0.052) | 0.302 (0.049) | 0.708 (0.025) | 0.962 (0.012) | 0.695 (0.034) | 0.886 (0.013) |
|  |  | **20** | 0.642 (0.057) | 0.178 (0.059) | 0.652 (0.084) | 0.357 (0.066) | 0.691 (0.035) | 0.900 (0.042) | 0.671 (0.047) | 0.824 (0.031) |
|  |  | **10** | 0.605 (0.088) | 0.340 (0.112) | 0.587 (0.099) | 0.421 (0.089) | 0.663 (0.086) | 0.773 (0.080) | 0.672 (0.062) | 0.746 (0.068) |
|  | **1000** | **138** | 0.584 (0.083) | 0.177 (0.055) | 0.621 (0.108) | 0.373 (0.075) | 0.768 (0.034) | 0.975 (0.012) | 0.756 (0.051) | 0.926 (0.025) |
|  |  | **80** | 0.607 (0.078) | 0.171 (0.067) | 0.637 (0.079) | 0.332 (0.063) | 0.742 (0.024) | 0.974 (0.013) | 0.717 (0.035) | 0.921 (0.023) |
|  |  | **40** | 0.593 (0.085) | 0.186 (0.091) | 0.600 (0.099) | 0.353 (0.118) | 0.731 (0.030) | 0.964 (0.016) | 0.730 (0.033) | 0.897 (0.020) |
|  |  | **30** | 0.598 (0.079) | 0.170 (0.093) | 0.571 (0.099) | 0.307 (0.095) | 0.705 (0.057) | 0.954 (0.015) | 0.695 (0.046) | 0.871 (0.023) |
|  |  | **20** | 0.627 (0.090) | 0.211 (0.092) | 0.619 (0.087) | 0.337 (0.083) | 0.702 (0.056) | 0.925 (0.036) | 0.683 (0.063) | 0.844 (0.033) |
|  |  | **10** | 0.573 (0.122) | 0.310 (0.099) | 0.592 (0.100) | 0.419 (0.108) | 0.672 (0.122) | 0.782 (0.061) | 0.665 (0.093) | 0.744 (0.059) |

## Average sensitivities and specificities of classifiers - test size 0.3 (Table A12)

**Table A12: Average sensitivities and specificities of classifiers for 6-month mortality prediction on balanced data set (test size 0.3****)**

| **Test Size** | **Sample**  **Size** | **Number of features** | **Average Sensitivity (SD)** | | | | **Average Specificity (SD)** | | | |
| --- | --- | --- | --- | --- | --- | --- | --- | --- | --- | --- |
|  |  |  | **RLR** | **RF** | **MLP** | **XGBoost** | **RLR** | **RF** | **MLP** | **XGBoost** |
| **0.3** | **95042** | **138** | 0.693 (0.006) | 0.144 (0.005) | 0.724 (0.037) | 0.539 (0.006) | 0.741 (0.004) | 0.980 (0.001) | 0.695 (0.035) | 0.854 (0.003) |
|  |  | **80** | 0.685 (0.009) | 0.131 (0.025) | 0.732 (0.036) | 0.537 (0.015) | 0.724 (0.013) | 0.980 (0.002) | 0.675 (0.031) | 0.835 (0.007) |
|  |  | **40** | 0.671 (0.013) | 0.128 (0.032) | 0.674 (0.056) | 0.548 (0.015) | 0.707 (0.017) | 0.972 (0.005) | 0.703 (0.049) | 0.796 (0.015) |
|  |  | **30** | 0.649 (0.030) | 0.163 (0.049) | 0.670 (0.048) | 0.543 (0.022) | 0.708 (0.022) | 0.944 (0.023) | 0.695 (0.043) | 0.779 (0.014) |
|  |  | **20** | 0.643 (0.049) | 0.245 (0.074) | 0.620 (0.064) | 0.555 (0.047) | 0.678 (0.027) | 0.863 (0.049) | 0.698 (0.055) | 0.735 (0.024) |
|  |  | **10** | 0.600 (0.061) | 0.461 (0.108) | 0.596 (0.067) | 0.555 (0.048) | 0.669 (0.056) | 0.721 (0.081) | 0.676 (0.065) | 0.691 (0.055) |
|  | **16000** | **138** | 0.690 (0.019) | 0.119 (0.014) | 0.744 (0.038) | 0.346 (0.016) | 0.745 (0.006) | 0.982 (0.003) | 0.678 (0.032) | 0.925 (0.005) |
|  |  | **80** | 0.679 (0.017) | 0.117 (0.023) | 0.742 (0.041) | 0.351 (0.028) | 0.735 (0.014) | 0.981 (0.003) | 0.654 (0.034) | 0.909 (0.006) |
|  |  | **40** | 0.665 (0.030) | 0.121 (0.031) | 0.720 (0.048) | 0.371 (0.027) | 0.702 (0.021) | 0.972 (0.005) | 0.646 (0.044) | 0.871 (0.013) |
|  |  | **30** | 0.658 (0.027) | 0.127 (0.044) | 0.706 (0.044) | 0.381 (0.034) | 0.706 (0.022) | 0.962 (0.012) | 0.652 (0.037) | 0.856 (0.015) |
|  |  | **20** | 0.650 (0.033) | 0.203 (0.055) | 0.683 (0.051) | 0.435 (0.043) | 0.694 (0.040) | 0.904 (0.042) | 0.661 (0.032) | 0.805 (0.032) |
|  |  | **10** | 0.591 (0.078) | 0.416 (0.078) | 0.622 (0.088) | 0.492 (0.055) | 0.688 (0.072) | 0.758 (0.067) | 0.666 (0.071) | 0.733 (0.053) |
|  | **4000** | **138** | 0.657 (0.048) | 0.067 (0.019) | 0.664 (0.067) | 0.246 (0.040) | 0.729 (0.016) | 0.990 (0.003) | 0.714 (0.049) | 0.947 (0.007) |
|  |  | **80** | 0.660 (0.038) | 0.063 (0.020) | 0.681 (0.049) | 0.241 (0.035) | 0.702 (0.021) | 0.988 (0.003) | 0.674 (0.044) | 0.934 (0.008) |
|  |  | **40** | 0.664 (0.033) | 0.071 (0.030) | 0.658 (0.056) | 0.242 (0.027) | 0.681 (0.024) | 0.979 (0.007) | 0.660 (0.048) | 0.912 (0.017) |
|  |  | **30** | 0.657 (0.044) | 0.092 (0.032) | 0.665 (0.061) | 0.267 (0.038) | 0.677 (0.040) | 0.969 (0.013) | 0.649 (0.052) | 0.891 (0.016) |
|  |  | **20** | 0.614 (0.061) | 0.149 (0.048) | 0.628 (0.086) | 0.318 (0.063) | 0.674 (0.029) | 0.911 (0.032) | 0.655 (0.054) | 0.834 (0.036) |
|  |  | **10** | 0.586 (0.131) | 0.335 (0.108) | 0.578 (0.111) | 0.417 (0.084) | 0.674 (0.087) | 0.769 (0.065) | 0.670 (0.072) | 0.742 (0.051) |
|  | **1000** | **138** | 0.578 (0.097) | 0.077 (0.047) | 0.538 (0.098) | 0.284 (0.073) | 0.779 (0.035) | 0.987 (0.005) | 0.797 (0.050) | 0.942 (0.011) |
|  |  | **80** | 0.605 (0.054) | 0.080 (0.045) | 0.575 (0.085) | 0.249 (0.053) | 0.756 (0.038) | 0.988 (0.007) | 0.764 (0.040) | 0.932 (0.020) |
|  |  | **40** | 0.614 (0.074) | 0.101 (0.068) | 0.591 (0.064) | 0.241 (0.060) | 0.711 (0.054) | 0.974 (0.012) | 0.737 (0.039) | 0.903 (0.018) |
|  |  | **30** | 0.639 (0.091) | 0.107 (0.079) | 0.615 (0.099) | 0.281 (0.079) | 0.693 (0.074) | 0.968 (0.015) | 0.714 (0.058) | 0.896 (0.025) |
|  |  | **20** | 0.615 (0.061) | 0.128 (0.077) | 0.617 (0.077) | 0.298 (0.086) | 0.692 (0.068) | 0.936 (0.030) | 0.702 (0.057) | 0.856 (0.034) |
|  |  | **10** | 0.580 (0.130) | 0.254 (0.135) | 0.581 (0.142) | 0.360 (0.129) | 0.623 (0.074) | 0.807 (0.081) | 0.620 (0.092) | 0.755 (0.073) |

## Average F1-scores of classifiers - test size 0.1 (Table A13)

**Table A13:**

**Average F1-scores of classifiers for 6-month mortality prediction on balanced data set (test size 0.1)**

| **Test Size** | **Sample**  **Size** | **Number of features** | **Average F1-Score (SD)** | | | |
| --- | --- | --- | --- | --- | --- | --- |
|  |  |  | **RLR** | **RF** | **MLP** | **XGBosst** |
| **0.1** | **95042** | **138** | 0.408 (0.008) | 0.229 (0.010) | 0.386 (0.012) | 0.433 (0.009) |
|  |  | **80** | 0.391 (0.010) | 0.215 (0.031) | 0.381 (0.012) | 0.412 (0.013) |
|  |  | **40** | 0.374 (0.011) | 0.192 (0.043) | 0.375 (0.014) | 0.383 (0.016) |
|  |  | **30** | 0.365 (0.016) | 0.200 (0.042) | 0.366 (0.017) | 0.364 (0.015) |
|  |  | **20** | 0.346 (0.016) | 0.220 (0.033) | 0.352 (0.017) | 0.348 (0.017) |
|  |  | **10** | 0.313 (0.025) | 0.269 (0.052) | 0.314 (0.027) | 0.306 (0.024) |
|  | **16000** | **138** | 0.402 (0.024) | 0.220 (0.029) | 0.380 (0.019) | 0.374 (0.030) |
|  |  | **80** | 0.390 (0.018) | 0.196 (0.045) | 0.376 (0.015) | 0.357 (0.035) |
|  |  | **40** | 0.374 (0.023) | 0.203 (0.061) | 0.357 (0.023) | 0.349 (0.031) |
|  |  | **30** | 0.351 (0.017) | 0.167 (0.047) | 0.343 (0.018) | 0.310 (0.026) |
|  |  | **20** | 0.342 (0.028) | 0.195 (0.043) | 0.344 (0.027) | 0.303 (0.031) |
|  |  | **10** | 0.314 (0.027) | 0.273 (0.033) | 0.313 (0.032) | 0.293 (0.031) |
|  | **4000** | **138** | 0.400 (0.049) | 0.163 (0.048) | 0.393 (0.051) | 0.382 (0.055) |
|  |  | **80** | 0.404 (0.051) | 0.190 (0.073) | 0.386 (0.054) | 0.357 (0.060) |
|  |  | **40** | 0.367 (0.053) | 0.164 (0.067) | 0.353 (0.051) | 0.292 (0.056) |
|  |  | **30** | 0.361 (0.058) | 0.210 (0.081) | 0.367 (0.052) | 0.317 (0.059) |
|  |  | **20** | 0.362 (0.034) | 0.199 (0.045) | 0.354 (0.034) | 0.265 (0.058) |
|  |  | **10** | 0.316 (0.051) | 0.255 (0.057) | 0.316 (0.055) | 0.286 (0.061) |
|  | **1000** | **138** | 0.375 (0.103) | 0.098 (0.099) | 0.344 (0.086) | 0.264 (0.105) |
|  |  | **80** | 0.344 (0.075) | 0.098 (0.114) | 0.348 (0.080) | 0.236 (0.122) |
|  |  | **40** | 0.394 (0.066) | 0.173 (0.079) | 0.360 (0.058) | 0.303 (0.100) |
|  |  | **30** | 0.352 (0.112) | 0.138 (0.111) | 0.336 (0.103) | 0.214 (0.113) |
|  |  | **20** | 0.352 (0.080) | 0.169 (0.091) | 0.336 (0.086) | 0.261 (0.084) |
|  |  | **10** | 0.308 (0.080) | 0.204 (0.119) | 0.293 (0.063) | 0.245 (0.079) |

RLR=Regularized Logistic Regression. RF=Random Forest. MLP=Multilayer Perceptron. XGBoost=eXtreme Gradient Boosting. SD=Standard Deviation

## Average F1-scores of classifiers - test size 0.2 (Table A14)

**Table A14:**

**Average F1- scores of classifiers for 6-month mortality prediction on balanced data set (test size 0.2)**

| **Test Size** | **Sample**  **Size** | **Number of features** | **Average F1-Score (SD)** | | | |
| --- | --- | --- | --- | --- | --- | --- |
|  |  |  | **RLR** | **RF** | **MLP** | **XGBosst** |
| **0.2** | **95042** | **138** | 0.409 (0.005) | 0.224 (0.006) | 0.384 (0.012) | 0.434 (0.006) |
|  |  | **80** | 0.389 (0.010) | 0.208 (0.041) | 0.377 (0.012) | 0.406 (0.014) |
|  |  | **40** | 0.372 (0.013) | 0.195 (0.042) | 0.371 (0.017) | 0.379 (0.014) |
|  |  | **30** | 0.354 (0.015) | 0.183 (0.042) | 0.360 (0.016) | 0.356 (0.018) |
|  |  | **20** | 0.348 (0.018) | 0.212 (0.038) | 0.351 (0.021) | 0.344 (0.020) |
|  |  | **10** | 0.326 (0.022) | 0.294 (0.021) | 0.323 (0.025) | 0.317 (0.021) |
|  | **16000** | **138** | 0.392 (0.013) | 0.189 (0.022) | 0.374 (0.013) | 0.355 (0.017) |
|  |  | **80** | 0.384 (0.014) | 0.168 (0.034) | 0.364 (0.014) | 0.343 (0.026) |
|  |  | **40** | 0.366 (0.021) | 0.184 (0.058) | 0.354 (0.020) | 0.331 (0.029) |
|  |  | **30** | 0.353 (0.017) | 0.173 (0.049) | 0.343 (0.017) | 0.310 (0.033) |
|  |  | **20** | 0.346 (0.015) | 0.186 (0.034) | 0.339 (0.015) | 0.301 (0.021) |
|  |  | **10** | 0.312 (0.031) | 0.255 (0.041) | 0.307 (0.033) | 0.287 (0.039) |
|  | **4000** | **138** | 0.407 (0.026) | 0.185 (0.029) | 0.397 (0.031) | 0.356 (0.038) |
|  |  | **80** | 0.404 (0.035) | 0.166 (0.043) | 0.397 (0.045) | 0.335 (0.039) |
|  |  | **40** | 0.384 (0.029) | 0.185 (0.054) | 0.374 (0.032) | 0.326 (0.046) |
|  |  | **30** | 0.368 (0.033) | 0.180 (0.067) | 0.360 (0.029) | 0.296 (0.042) |
|  |  | **20** | 0.352 (0.037) | 0.191 (0.049) | 0.343 (0.033) | 0.284 (0.039) |
|  |  | **10** | 0.326 (0.039) | 0.241 (0.055) | 0.321 (0.042) | 0.277 (0.038) |
|  | **1000** | **138** | 0.386 (0.049) | 0.262 (0.067) | 0.396 (0.052) | 0.404 (0.062) |
|  |  | **80** | 0.394 (0.065) | 0.254 (0.084) | 0.391 (0.069) | 0.371 (0.070) |
|  |  | **40** | 0.386 (0.057) | 0.262 (0.117) | 0.389 (0.057) | 0.362 (0.098) |
|  |  | **30** | 0.362 (0.066) | 0.230 (0.112) | 0.343 (0.078) | 0.296 (0.085) |
|  |  | **20** | 0.377 (0.045) | 0.252 (0.089) | 0.362 (0.048) | 0.301 (0.064) |
|  |  | **10** | 0.336 (0.078) | 0.238 (0.067) | 0.341 (0.076) | 0.285 (0.060) |

RLR=Regularized Logistic Regression. RF=Random Forest. MLP=Multilayer Perceptron. XGBoost=eXtreme Gradient Boosting. SD=Standard Deviation

## Average F1-scores of classifiers - test size 0.3 (Table A15)

**Table A15:**

**Average F1- scores of classifiers for 6-month mortality prediction on balanced data set (test size 0.3)**

| **Test Size** | **Sample**  **Size** | **Number of features** | **Average F1-Score (SD)** | | | |
| --- | --- | --- | --- | --- | --- | --- |
|  |  |  | **RLR** | **RF** | **MLP** | **XGBosst** |
| **0.3** | **95042** | **138** | 0.404 (0.006) | 0.226 (0.006) | 0.386 (0.010) | 0.429 (0.006) |
|  |  | **80** | 0.389 (0.008) | 0.206 (0.034) | 0.376 (0.009) | 0.407 (0.011) |
|  |  | **40** | 0.371 (0.011) | 0.193 (0.042) | 0.370 (0.014) | 0.377 (0.014) |
|  |  | **30** | 0.361 (0.010) | 0.209 (0.038) | 0.363 (0.014) | 0.360 (0.012) |
|  |  | **20** | 0.340 (0.023) | 0.223 (0.036) | 0.343 (0.023) | 0.334 (0.025) |
|  |  | **10** | 0.316 (0.031) | 0.275 (0.038) | 0.319 (0.030) | 0.308 (0.029) |
|  | **16000** | **138** | 0.408 (0.011) | 0.191 (0.021) | 0.384 (0.014) | 0.375 (0.010) |
|  |  | **80** | 0.399 (0.012) | 0.187 (0.030) | 0.372 (0.012) | 0.361 (0.020) |
|  |  | **40** | 0.369 (0.020) | 0.184 (0.041) | 0.358 (0.018) | 0.336 (0.021) |
|  |  | **30** | 0.368 (0.013) | 0.181 (0.051) | 0.356 (0.015) | 0.328 (0.021) |
|  |  | **20** | 0.357 (0.019) | 0.219 (0.035) | 0.350 (0.021) | 0.321 (0.023) |
|  |  | **10** | 0.327 (0.024) | 0.278 (0.028) | 0.329 (0.026) | 0.306 (0.027) |
|  | **4000** | **138** | 0.368 (0.019) | 0.117 (0.032) | 0.362 (0.018) | 0.302 (0.039) |
|  |  | **80** | 0.365 (0.020) | 0.109 (0.032) | 0.357 (0.021) | 0.287 (0.036) |
|  |  | **40** | 0.345 (0.017) | 0.116 (0.044) | 0.330 (0.023) | 0.262 (0.033) |
|  |  | **30** | 0.342 (0.026) | 0.139 (0.040) | 0.329 (0.024) | 0.265 (0.032) |
|  |  | **20** | 0.318 (0.029) | 0.166 (0.037) | 0.313 (0.029) | 0.257 (0.036) |
|  |  | **10** | 0.306 (0.027) | 0.225 (0.042) | 0.302 (0.030) | 0.261 (0.025) |
|  | **1000** | **138** | 0.368 (0.043) | 0.128 (0.074) | 0.363 (0.040) | 0.331 (0.067) |
|  |  | **80** | 0.365 (0.041) | 0.134 (0.069) | 0.355 (0.048) | 0.286 (0.052) |
|  |  | **40** | 0.350 (0.046) | 0.153 (0.093) | 0.355 (0.041) | 0.254 (0.056) |
|  |  | **30** | 0.339 (0.054) | 0.154 (0.103) | 0.342 (0.062) | 0.279 (0.074) |
|  |  | **20** | 0.331 (0.059) | 0.157 (0.085) | 0.335 (0.054) | 0.259 (0.066) |
|  |  | **10** | 0.270 (0.060) | 0.181 (0.051) | 0.270 (0.070) | 0.226 (0.059) |

RLR=Regularized Logistic Regression. RF=Random Forest. MLP=Multilayer Perceptron. XGBoost=eXtreme Gradient Boosting. SD=Standard Deviation

## Average accuracies and precisions of classifiers - test size 0.1 (Table A16)

**Table A16: Average accuracies and precisions of classifiers for 6-month mortality prediction on balanced data set (test size 0.1)**

| **Test Size** | **Sample**  **Size** | **Number of features** | **Average Accuracy (SD)** | | | | **Average Precision (SD)** | | | | |
| --- | --- | --- | --- | --- | --- | --- | --- | --- | --- | --- | --- |
|  |  |  | **RLR** | **RF** | **MLP** | **XGBoost** | **RLR** | **RF** | **MLP** | **XGBoost** |  |
| **0.1** | **95042** | **138** | 0.734 (0.004) | 0.871 (0.002) | 0.686 (0.027) | 0.806 (0.004) | 0.287 (0.006) | 0.525 (0.023) | 0.260 (0.014) | 0.350 (0.008) |  |
|  |  | **80** | 0.722 (0.010) | 0.870 (0.003) | 0.693 (0.024) | 0.790 (0.006) | 0.274 (0.009) | 0.499 (0.038) | 0.259 (0.013) | 0.324 (0.011) |  |
|  |  | **40** | 0.699 (0.015) | 0.860 (0.005) | 0.699 (0.025) | 0.756 (0.012) | 0.258 (0.009) | 0.407 (0.040) | 0.260 (0.016) | 0.288 (0.016) |  |
|  |  | **30** | 0.694 (0.018) | 0.845 (0.012) | 0.706 (0.034) | 0.738 (0.011) | 0.251 (0.014) | 0.318 (0.048) | 0.257 (0.021) | 0.267 (0.014) |  |
|  |  | **20** | 0.677 (0.021) | 0.800 (0.026) | 0.686 (0.037) | 0.715 (0.014) | 0.236 (0.013) | 0.232 (0.033) | 0.242 (0.018) | 0.248 (0.013) |  |
|  |  | **10** | 0.643 (0.046) | 0.676 (0.070) | 0.664 (0.048) | 0.647 (0.041) | 0.210 (0.019) | 0.196 (0.019) | 0.216 (0.023) | 0.207 (0.018) |  |
|  | **16000** | **138** | 0.739 (0.010) | 0.874 (0.007) | 0.703 (0.032) | 0.849 (0.007) | 0.284 (0.021) | 0.522 (0.080) | 0.260 (0.019) | 0.396 (0.039) |  |
|  |  | **80** | 0.725 (0.015) | 0.869 (0.008) | 0.689 (0.031) | 0.837 (0.009) | 0.275 (0.015) | 0.490 (0.063) | 0.256 (0.016) | 0.368 (0.029) |  |
|  |  | **40** | 0.706 (0.017) | 0.866 (0.010) | 0.658 (0.027) | 0.807 (0.018) | 0.259 (0.020) | 0.444 (0.092) | 0.237 (0.018) | 0.312 (0.033) |  |
|  |  | **30** | 0.693 (0.021) | 0.852 (0.012) | 0.665 (0.041) | 0.783 (0.016) | 0.241 (0.017) | 0.305 (0.079) | 0.231 (0.019) | 0.264 (0.031) |  |
|  |  | **20** | 0.681 (0.040) | 0.809 (0.030) | 0.682 (0.039) | 0.754 (0.020) | 0.235 (0.027) | 0.222 (0.040) | 0.235 (0.025) | 0.239 (0.025) |  |
|  |  | **10** | 0.655 (0.060) | 0.682 (0.037) | 0.657 (0.065) | 0.673 (0.040) | 0.214 (0.025) | 0.194 (0.023) | 0.213 (0.028) | 0.204 (0.025) |  |
|  | **4000** | **138** | 0.735 (0.023) | 0.861 (0.016) | 0.729 (0.038) | 0.855 (0.018) | 0.291 (0.045) | 0.476 (0.146) | 0.287 (0.052) | 0.463 (0.081) |  |
|  |  | **80** | 0.730 (0.027) | 0.864 (0.014) | 0.707 (0.035) | 0.846 (0.017) | 0.290 (0.042) | 0.493 (0.162) | 0.272 (0.045) | 0.414 (0.072) |  |
|  |  | **40** | 0.696 (0.029) | 0.858 (0.017) | 0.651 (0.058) | 0.819 (0.020) | 0.255 (0.043) | 0.385 (0.146) | 0.237 (0.045) | 0.306 (0.059) |  |
|  |  | **30** | 0.695 (0.034) | 0.849 (0.018) | 0.684 (0.036) | 0.803 (0.025) | 0.256 (0.048) | 0.382 (0.126) | 0.256 (0.045) | 0.309 (0.062) |  |
|  |  | **20** | 0.692 (0.032) | 0.816 (0.026) | 0.672 (0.039) | 0.762 (0.026) | 0.257 (0.032) | 0.266 (0.061) | 0.247 (0.030) | 0.236 (0.048) |  |
|  |  | **10** | 0.646 (0.059) | 0.686 (0.071) | 0.636 (0.070) | 0.681 (0.063) | 0.216 (0.042) | 0.193 (0.047) | 0.215 (0.047) | 0.209 (0.053) |  |
|  | **1000** | **138** | 0.714 (0.050) | 0.849 (0.037) | 0.698 (0.042) | 0.818 (0.037) | 0.275 (0.089) | 0.258 (0.272) | 0.251 (0.070) | 0.317 (0.148) |  |
|  |  | **80** | 0.685 (0.047) | 0.858 (0.044) | 0.686 (0.055) | 0.814 (0.047) | 0.241 (0.065) | 0.329 (0.405) | 0.244 (0.066) | 0.264 (0.142) |  |
|  |  | **40** | 0.684 (0.045) | 0.828 (0.038) | 0.658 (0.048) | 0.791 (0.043) | 0.286 (0.058) | 0.415 (0.235) | 0.258 (0.051) | 0.317 (0.088) |  |
|  |  | **30** | 0.651 (0.066) | 0.832 (0.039) | 0.641 (0.063) | 0.776 (0.050) | 0.245 (0.090) | 0.247 (0.198) | 0.234 (0.084) | 0.223 (0.121) |  |
|  |  | **20** | 0.653 (0.053) | 0.797 (0.040) | 0.636 (0.065) | 0.753 (0.038) | 0.254 (0.076) | 0.288 (0.213) | 0.241 (0.080) | 0.262 (0.120) |  |
|  |  | **10** | 0.624 (0.099) | 0.745 (0.069) | 0.625 (0.084) | 0.716 (0.080) | 0.212 (0.066) | 0.179 (0.103) | 0.202 (0.051) | 0.207 (0.087) |  |

## Average accuracies and precisions of classifiers - test size 0.2 (Table A17)

**Table A17: Average accuracies and precisions of classifiers for 6-month mortality prediction on balanced data set (test size 0.2)**

| **Test Size** | **Sample Size** | **Number of features** | **Average Accuracy (SD)** | | | | **Average Precision (SD)** | | | | |
| --- | --- | --- | --- | --- | --- | --- | --- | --- | --- | --- | --- |
|  |  |  | **RLR** | **RF** | **MLP** | **XGBoost** | **RLR** | **RF** | **MLP** | **XGBoost** |  |
| **0.2** | **95042** | **138** | 0.734 (0.003) | 0.869 (0.002) | 0.678 (0.034) | 0.809 (0.003) | 0.289 (0.005) | 0.516 (0.014) | 0.258 (0.015) | 0.355 (0.006) |  |
|  |  | **80** | 0.720 (0.011) | 0.869 (0.003) | 0.684 (0.028) | 0.790 (0.009) | 0.272 (0.009) | 0.491 (0.032) | 0.254 (0.013) | 0.322 (0.014) |  |
|  |  | **40** | 0.700 (0.014) | 0.862 (0.005) | 0.695 (0.034) | 0.759 (0.010) | 0.256 (0.012) | 0.412 (0.055) | 0.256 (0.020) | 0.287 (0.013) |  |
|  |  | **30** | 0.687 (0.019) | 0.842 (0.016) | 0.699 (0.029) | 0.738 (0.016) | 0.243 (0.013) | 0.296 (0.057) | 0.251 (0.017) | 0.264 (0.015) |  |
|  |  | **20** | 0.678 (0.022) | 0.806 (0.031) | 0.693 (0.040) | 0.715 (0.017) | 0.238 (0.015) | 0.241 (0.039) | 0.245 (0.023) | 0.247 (0.018) |  |
|  |  | **10** | 0.660 (0.036) | 0.682 (0.029) | 0.677 (0.053) | 0.670 (0.022) | 0.220 (0.019) | 0.207 (0.015) | 0.226 (0.023) | 0.218 (0.016) |  |
|  | **16000** | **138** | 0.732 (0.007) | 0.875 (0.005) | 0.696 (0.033) | 0.851 (0.005) | 0.275 (0.011) | 0.536 (0.051) | 0.255 (0.018) | 0.394 (0.024) |  |
|  |  | **80** | 0.724 (0.012) | 0.871 (0.006) | 0.680 (0.026) | 0.839 (0.008) | 0.269 (0.013) | 0.490 (0.060) | 0.245 (0.015) | 0.360 (0.030) |  |
|  |  | **40** | 0.707 (0.022) | 0.866 (0.007) | 0.672 (0.034) | 0.812 (0.016) | 0.252 (0.018) | 0.395 (0.089) | 0.236 (0.018) | 0.302 (0.036) |  |
|  |  | **30** | 0.697 (0.019) | 0.855 (0.008) | 0.660 (0.029) | 0.792 (0.013) | 0.242 (0.016) | 0.313 (0.073) | 0.227 (0.013) | 0.267 (0.026) |  |
|  |  | **20** | 0.677 (0.024) | 0.813 (0.026) | 0.652 (0.035) | 0.751 (0.020) | 0.234 (0.014) | 0.220 (0.038) | 0.225 (0.014) | 0.236 (0.019) |  |
|  |  | **10** | 0.647 (0.055) | 0.700 (0.057) | 0.660 (0.046) | 0.685 (0.045) | 0.210 (0.026) | 0.190 (0.027) | 0.210 (0.023) | 0.204 (0.032) |  |
|  | **4000** | **138** | 0.749 (0.009) | 0.864 (0.011) | 0.725 (0.032) | 0.853 (0.010) | 0.299 (0.024) | 0.481 (0.079) | 0.283 (0.032) | 0.438 (0.058) |  |
|  |  | **80** | 0.737 (0.021) | 0.862 (0.013) | 0.712 (0.038) | 0.847 (0.011) | 0.295 (0.035) | 0.490 (0.113) | 0.282 (0.044) | 0.418 (0.055) |  |
|  |  | **40** | 0.711 (0.023) | 0.853 (0.009) | 0.685 (0.033) | 0.827 (0.010) | 0.273 (0.024) | 0.399 (0.086) | 0.259 (0.026) | 0.354 (0.045) |  |
|  |  | **30** | 0.699 (0.023) | 0.848 (0.014) | 0.688 (0.028) | 0.807 (0.016) | 0.258 (0.029) | 0.335 (0.091) | 0.250 (0.025) | 0.293 (0.041) |  |
|  |  | **20** | 0.685 (0.031) | 0.804 (0.034) | 0.668 (0.035) | 0.762 (0.024) | 0.243 (0.030) | 0.219 (0.058) | 0.234 (0.025) | 0.238 (0.034) |  |
|  |  | **10** | 0.655 (0.067) | 0.714 (0.058) | 0.661 (0.047) | 0.702 (0.050) | 0.226 (0.036) | 0.193 (0.042) | 0.222 (0.031) | 0.211 (0.033) |  |
|  | **1000** | **138** | 0.742 (0.026) | 0.863 (0.017) | 0.737 (0.037) | 0.848 (0.015) | 0.292 (0.051) | 0.551 (0.134) | 0.295 (0.046) | 0.460 (0.111) |  |
|  |  | **80** | 0.723 (0.026) | 0.854 (0.020) | 0.705 (0.032) | 0.833 (0.022) | 0.293 (0.059) | 0.541 (0.132) | 0.285 (0.061) | 0.430 (0.107) |  |
|  |  | **40** | 0.709 (0.031) | 0.843 (0.026) | 0.710 (0.028) | 0.813 (0.021) | 0.288 (0.048) | 0.478 (0.171) | 0.291 (0.048) | 0.381 (0.090) |  |
|  |  | **30** | 0.689 (0.050) | 0.840 (0.023) | 0.677 (0.044) | 0.789 (0.024) | 0.263 (0.059) | 0.377 (0.150) | 0.247 (0.064) | 0.290 (0.084) |  |
|  |  | **20** | 0.691 (0.044) | 0.818 (0.035) | 0.674 (0.051) | 0.768 (0.035) | 0.272 (0.041) | 0.339 (0.110) | 0.259 (0.044) | 0.277 (0.064) |  |
|  |  | **10** | 0.656 (0.093) | 0.709 (0.043) | 0.654 (0.077) | 0.693 (0.043) | 0.248 (0.078) | 0.204 (0.070) | 0.245 (0.071) | 0.224 (0.055) |  |

RLR=Regularized Logistic Regression. RF=Random Forest. MLP=Multilayer Perceptron. XGBoost=eXtreme Gradient Boosting. SD=Standard Deviation

## Average accuracies and precisions of classifiers - test size 0.3 (Table A18)

**Table A18: Average accuracies and precisions of classifiers for 6-month mortality prediction on balanced data set (test size 0.3)**

| **Test Size** | **Sample Size** | **Number of features** | **Average Accuracy (SD)** | | | | **Average Precision (SD)** | | | | |
| --- | --- | --- | --- | --- | --- | --- | --- | --- | --- | --- | --- |
|  |  |  | **RLR** | **RF** | **MLP** | **XGBoost** | **RLR** | **RF** | **MLP** | **XGBoost** |  |
| **0.3** | **95042** | **138** | 0.734 (0.003) | 0.871 (0.002) | 0.699 (0.026) | 0.813 (0.003) | 0.286 (0.005) | 0.516 (0.012) | 0.263 (0.013) | 0.356 (0.007) |  |
|  |  | **80** | 0.718 (0.011) | 0.869 (0.002) | 0.683 (0.023) | 0.796 (0.006) | 0.271 (0.009) | 0.495 (0.030) | 0.254 (0.011) | 0.328 (0.011) |  |
|  |  | **40** | 0.703 (0.014) | 0.861 (0.005) | 0.699 (0.036) | 0.763 (0.012) | 0.257 (0.011) | 0.402 (0.053) | 0.257 (0.018) | 0.288 (0.015) |  |
|  |  | **30** | 0.700 (0.016) | 0.842 (0.015) | 0.692 (0.032) | 0.748 (0.011) | 0.251 (0.010) | 0.314 (0.046) | 0.250 (0.018) | 0.270 (0.012) |  |
|  |  | **20** | 0.674 (0.022) | 0.782 (0.034) | 0.688 (0.042) | 0.711 (0.020) | 0.231 (0.017) | 0.214 (0.024) | 0.239 (0.024) | 0.239 (0.018) |  |
|  |  | **10** | 0.660 (0.046) | 0.688 (0.060) | 0.666 (0.052) | 0.673 (0.046) | 0.216 (0.028) | 0.201 (0.023) | 0.219 (0.026) | 0.215 (0.026) |  |
|  | **16000** | **138** | 0.738 (0.005) | 0.869 (0.005) | 0.687 (0.024) | 0.849 (0.004) | 0.290 (0.010) | 0.502 (0.059) | 0.260 (0.015) | 0.410 (0.015) |  |
|  |  | **80** | 0.728 (0.011) | 0.866 (0.004) | 0.666 (0.025) | 0.835 (0.005) | 0.283 (0.012) | 0.481 (0.034) | 0.249 (0.012) | 0.373 (0.016) |  |
|  |  | **40** | 0.697 (0.018) | 0.858 (0.004) | 0.656 (0.033) | 0.804 (0.011) | 0.256 (0.016) | 0.394 (0.053) | 0.239 (0.017) | 0.308 (0.024) |  |
|  |  | **30** | 0.699 (0.017) | 0.851 (0.008) | 0.659 (0.028) | 0.793 (0.012) | 0.256 (0.013) | 0.335 (0.044) | 0.239 (0.015) | 0.290 (0.021) |  |
|  |  | **20** | 0.688 (0.032) | 0.811 (0.031) | 0.664 (0.025) | 0.756 (0.025) | 0.246 (0.018) | 0.256 (0.050) | 0.236 (0.017) | 0.256 (0.025) |  |
|  |  | **10** | 0.675 (0.055) | 0.712 (0.050) | 0.660 (0.053) | 0.701 (0.042) | 0.230 (0.030) | 0.214 (0.029) | 0.226 (0.024) | 0.224 (0.027) |  |
|  | **4000** | **138** | 0.720 (0.013) | 0.875 (0.007) | 0.708 (0.037) | 0.859 (0.009) | 0.256 (0.015) | 0.498 (0.093) | 0.250 (0.018) | 0.396 (0.046) |  |
|  |  | **80** | 0.696 (0.017) | 0.865 (0.006) | 0.675 (0.033) | 0.843 (0.008) | 0.253 (0.018) | 0.431 (0.083) | 0.243 (0.021) | 0.359 (0.042) |  |
|  |  | **40** | 0.679 (0.019) | 0.864 (0.009) | 0.660 (0.037) | 0.826 (0.015) | 0.233 (0.016) | 0.341 (0.110) | 0.222 (0.021) | 0.290 (0.055) |  |
|  |  | **30** | 0.675 (0.031) | 0.856 (0.011) | 0.651 (0.039) | 0.811 (0.012) | 0.232 (0.023) | 0.312 (0.080) | 0.219 (0.021) | 0.267 (0.036) |  |
|  |  | **20** | 0.667 (0.021) | 0.815 (0.023) | 0.652 (0.038) | 0.768 (0.026) | 0.215 (0.023) | 0.204 (0.063) | 0.210 (0.022) | 0.220 (0.039) |  |
|  |  | **10** | 0.662 (0.061) | 0.714 (0.045) | 0.658 (0.052) | 0.700 (0.035) | 0.211 (0.019) | 0.174 (0.020) | 0.206 (0.022) | 0.192 (0.014) |  |
|  | **1000** | **138** | 0.753 (0.026) | 0.873 (0.015) | 0.765 (0.035) | 0.859 (0.014) | 0.273 (0.035) | 0.427 (0.207) | 0.280 (0.043) | 0.407 (0.060) |  |
|  |  | **80** | 0.737 (0.030) | 0.875 (0.013) | 0.740 (0.031) | 0.846 (0.019) | 0.263 (0.040) | 0.473 (0.217) | 0.259 (0.044) | 0.351 (0.084) |  |
|  |  | **40** | 0.698 (0.043) | 0.859 (0.021) | 0.718 (0.030) | 0.815 (0.022) | 0.247 (0.041) | 0.361 (0.177) | 0.256 (0.040) | 0.274 (0.070) |  |
|  |  | **30** | 0.686 (0.061) | 0.861 (0.018) | 0.702 (0.051) | 0.819 (0.026) | 0.233 (0.045) | 0.314 (0.191) | 0.239 (0.050) | 0.283 (0.078) |  |
|  |  | **20** | 0.682 (0.056) | 0.834 (0.023) | 0.690 (0.043) | 0.786 (0.025) | 0.230 (0.056) | 0.243 (0.181) | 0.235 (0.053) | 0.235 (0.075) |  |
|  |  | **10** | 0.617 (0.065) | 0.738 (0.055) | 0.615 (0.075) | 0.707 (0.056) | 0.178 (0.045) | 0.161 (0.079) | 0.179 (0.054) | 0.171 (0.047) |  |

# Sample characteristics of 12-month mortality (Table A19)

| **CHARACTERISTICS** | **HC** | **12-month Deceased** | **12-month Survived** |
| --- | --- | --- | --- |
|  | Total n=95,042 n, (%) | Total n=18,870 n, (%) | Total n=76,272 n, (%) |
| **Age** |  |  |  |
| **65-69 years** | 5,906 (6.21) | 963 (5.13) | 4,943 (6.48) |
| **70-74 years** | 9,623 (10.12) | 1,601 (8.53) | 8,022 (10.52) |
| **75-79 years** | 15,284 (16.08) | 2,581 (13.75) | 12,703 (16.65) |
| **80-84 years** | 21,947 (23.09) | 4,027 (21.45) | 17,920 (23.49) |
| **85-89 years** | 23,906 (25.15) | 5,024 (26.77) | 18,882 (24.76) |
| **90-94 years** | 14,370 (15.12) | 3,404 (18.14) | 10,966 (14.38) |
| **95-99 years** | 3,594 (3.78) | 1,033 (5.50) | 2,561 (3.36) |
| **100+ years** | 412 (0.43) | 137 (0.73) | 275 (0.36) |
| **Mean (SD)** | 82.66 (7.61) | 83.80 (7.67) | 83.28 (7.57) |
| **Gender** |  |  |  |
| **Female** | 57,580 (60.58) | 9,754 (51.97) | 47,826 (62.70) |
| **Male** | 37,462 (39.42) | 9,016 (48.03) | 28,446 (37.30) |
| **Ethnicity** |  |  |  |
| **European** | 83,590 (87.95) | 16,881 (89.94) | 66,709 (87.46) |
| **Maori** | 5,321 (5.60) | 1,045 (5.57) | 4,276 (5.61) |
| **Pacific Island** | 2,948 (3.10) | 426 (2.27) | 2,522 (3.31) |
| **Asian** | 2,304 (2.42) | 298 (1.59) | 2,006 (2.63) |
| **Middle Eastern/Latin American/African** | 352 (0.37) | 42 (0.22) | 310 (0.41) |
| **Other Ethnicity** | 527 (0.55) | 78 (0.42) | 449 (0.59) |
| **Marital status** |  |  |  |
| **Married/Civil Union/De facto** | 82,401 (86.70) | 16,632 (88.61) | 65,769 (86.23) |
| **Never married** | 4,486 (4.72) | 799 (4.26) | 3,687 (4.83) |
| **Widowed** | 2,116 (2.23) | 346 (1.84) | 1,770 (2.32) |
| **Separated and divorced** | 5,999 (6.31) | 989 (5.27) | 5,010 (6.57) |
| **Others** | 40 (0.04) | 4 (0.02) | 36 (0.05) |

# 12-month mortality prediction on balanced data set

## Average AUCs of classifiers and frailty scale (Figure A1)

**Figure A1: Average AUCs of classifiers and frailty scale for 12-month mortality prediction on balanced data set**


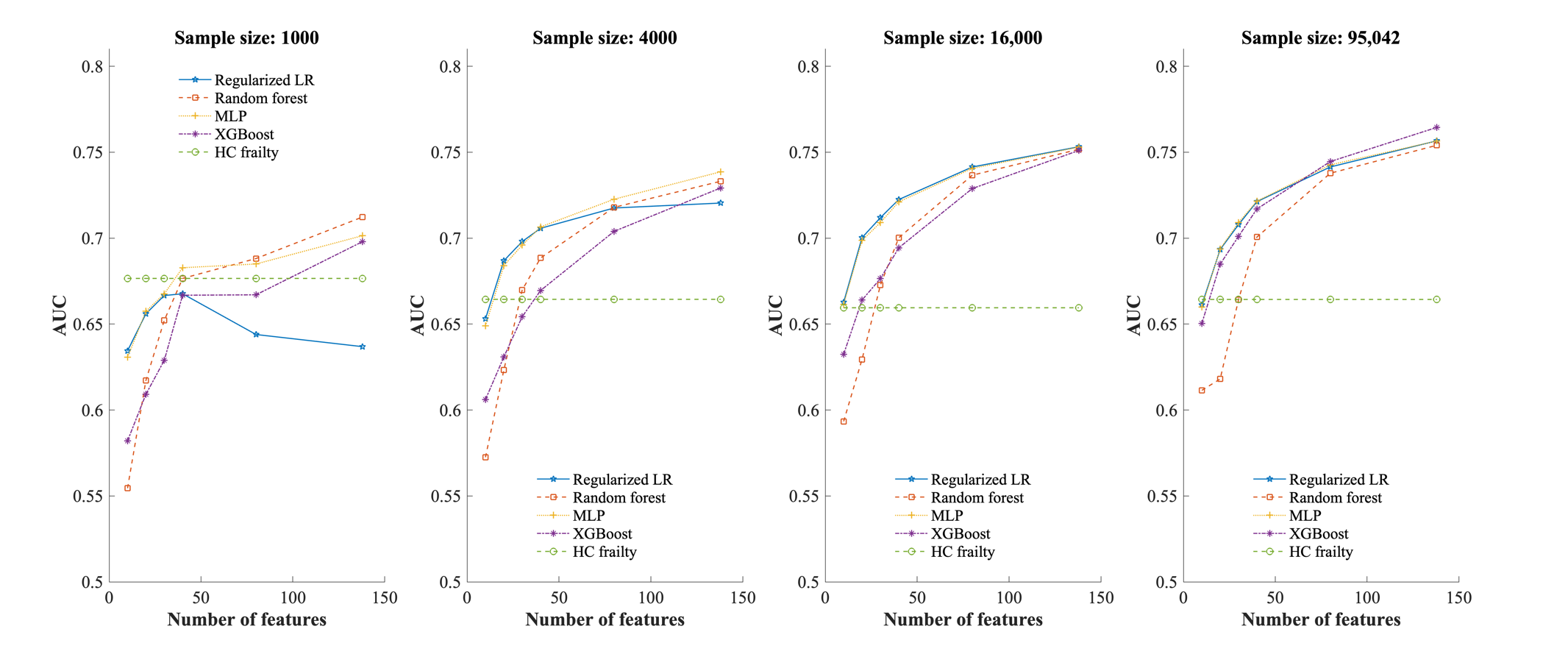
 Regularized LR=Regularized Logistic Regression. MLP=Multilayer Perceptron. XGBoost=eXtreme Gradient Boosting. HC Frailty=interRAI Home Care Frailty Scale.

## Average sensitivities of classifiers and frailty scale (Figure A2)

**Figure A2: Average sensitivities of classifiers and frailty scale for 12-month mortality prediction on balanced data set**


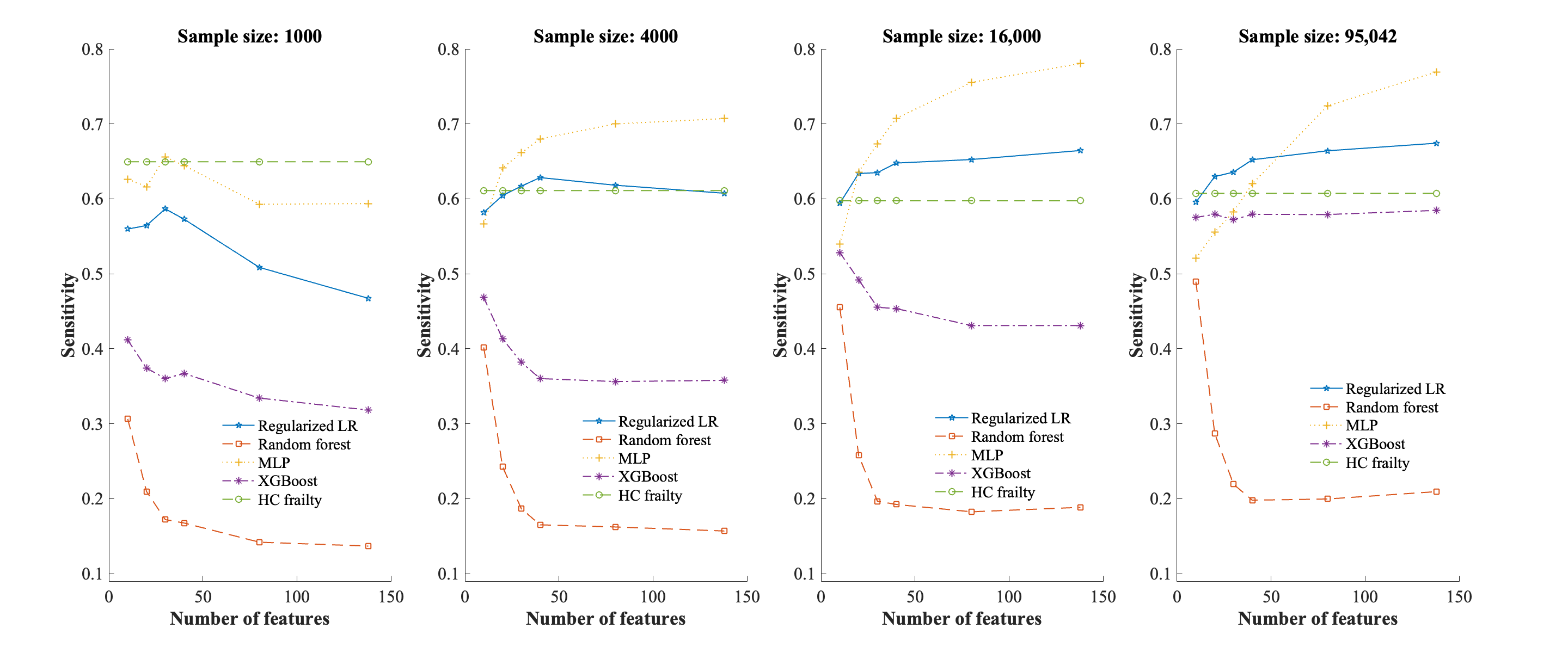
 Regularized LR=Regularized Logistic Regression. MLP=Multilayer Perceptron. XGBoost=eXtreme Gradient Boosting. HC Frailty=interRAI Home Care Frailty Scale.

## Average specificities of classifiers and frailty scale (Figure A3)

**Figure A3: Average specificities classifiers and frailty scale for 12-month mortality prediction on balanced data set**

**
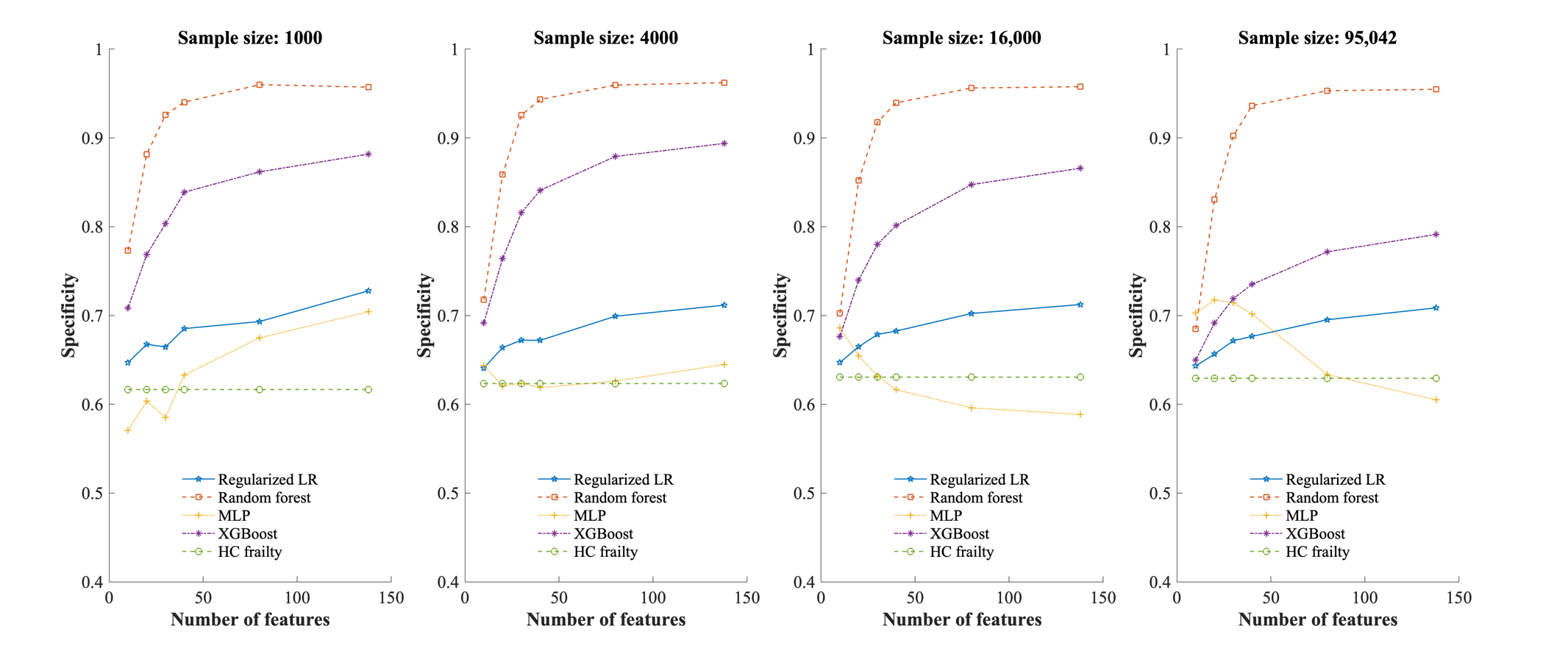
** Regularized LR=Regularized Logistic Regression. MLP=Multilayer Perceptron. XGBoost=eXtreme Gradient Boosting. HC Frailty=interRAI Home Care Frailty Scale.

## Average AUCs, sensitivities, and specificities of classifiers by test sizes (Figure A4)

**Figure A4: Average AUCs, sensitivities, and specificities of classifiers for 12-month mortality prediction by test sizes on balanced data set**


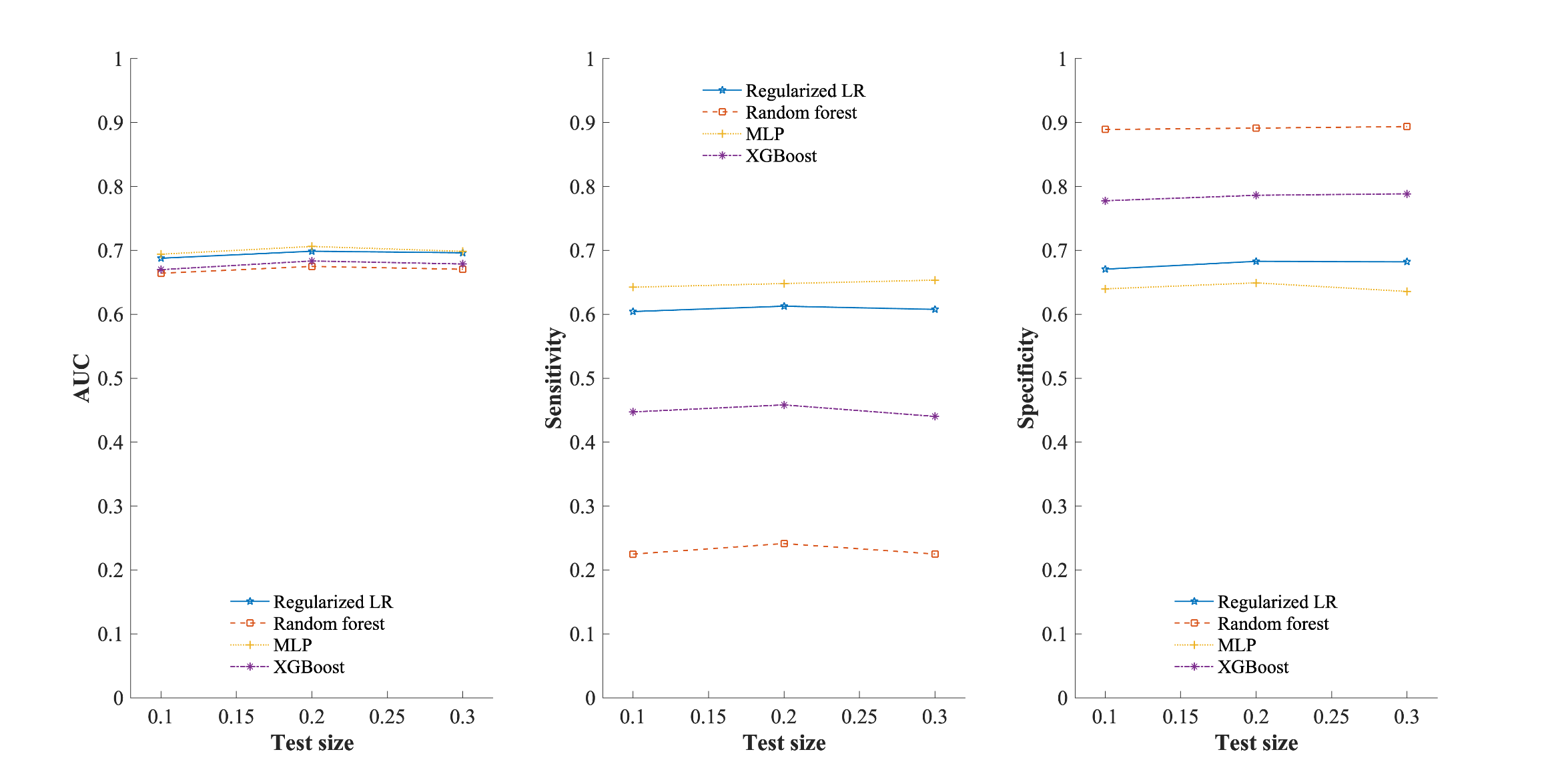
Regularized LR=Regularized Logistic Regression. RF=Random Forest. MLP=Multilayer Perceptron. XGBoost=eXtreme Gradient Boosting.

## Performance evaluation of frailty scale (Table A20 & A21)

**Table A20:**

**Average AUCs, sensitivities, and specificities of frailty scale for 12-month mortality prediction on balanced data set**

| **Sample Size** | **interRAI Home Care Frailty** | | |
| --- | --- | --- | --- |
|  | **Average AUC (SD)** | **Average Sensitivity (SD)** | **Average Specificity (SD)** |
| **1000** | 0.677 (0.019) | 0.650 (0.021) | 0.617 (0.024) |
| **4000** | 0.664 (0.003) | 0.611 (0.003) | 0.623 (0.004) |
| **16000** | 0.660 (0.001) | 0.597 (0.004) | 0.631 (0.002) |
| **95042** | 0.664 (0.000) | 0.607 (0.001) | 0.629 (0.000) |

SD=Standard Deviation

**Table A21:**

**Average accuracies, precisions, and F1-scores of frailty scale for 12-month mortality prediction on balanced data set**

| **Sample Size** | **interRAI Home Care Frailty** | | |
| --- | --- | --- | --- |
|  | **Average Accuracy (SD)** | **Average Precision (SD)** | **Average F1-score (SD)** |
| **1000** | 0.633 (0.021) | 0.629 (0.021) | 0.639 (0.021) |
| **4000** | 0.617 (0.002) | 0.619 (0.003) | 0.615 (0.002) |
| **16000** | 0.614 (0.002) | 0.618 (0.002) | 0.608 (0.003) |
| **95042** | 0.618 (0.000) | 0.621 (0.000) | 0.614 (0.000) |

SD=Standard Deviation

## Average AUCs of classifiers – test size 0.1 (Table A22)

**Table A22:**

**Average AUCs of classifiers for 12-month mortality prediction on balanced data set (test size 0.1)**

| **Test Size** | **Sample Size** | **Number of features** | **Average AUC (SD)** | | | |
| --- | --- | --- | --- | --- | --- | --- |
|  |  |  | **RLR** | **RF** | **MLP** | **XGBoost** |
| **0.1** | **95042** | **138** | 0.758 (0.006) | 0.757 (0.007) | 0.759 (0.006) | 0.767 (0.006) |
|  |  | **80** | 0.743 (0.008) | 0.740 (0.008) | 0.745 (0.007) | 0.746 (0.008) |
|  |  | **40** | 0.721 (0.009) | 0.702 (0.015) | 0.723 (0.010) | 0.718 (0.012) |
|  |  | **30** | 0.713 (0.011) | 0.669 (0.018) | 0.713 (0.011) | 0.706 (0.010) |
|  |  | **20** | 0.697 (0.017) | 0.620 (0.026) | 0.699 (0.018) | 0.692 (0.017) |
|  |  | **10** | 0.656 (0.032) | 0.613 (0.030) | 0.655 (0.031) | 0.648 (0.031) |
|  | **16000** | **138** | 0.750 (0.011) | 0.754 (0.015) | 0.751 (0.012) | 0.753 (0.014) |
|  |  | **80** | 0.741 (0.015) | 0.739 (0.014) | 0.742 (0.015) | 0.732 (0.013) |
|  |  | **40** | 0.722 (0.019) | 0.702 (0.027) | 0.722 (0.021) | 0.696 (0.021) |
|  |  | **30** | 0.707 (0.017) | 0.665 (0.026) | 0.704 (0.019) | 0.672 (0.021) |
|  |  | **20** | 0.693 (0.023) | 0.624 (0.029) | 0.692 (0.024) | 0.661 (0.023) |
|  |  | **10** | 0.665 (0.031) | 0.597 (0.035) | 0.661 (0.031) | 0.633 (0.031) |
|  | **4000** | **138** | 0.732 (0.026) | 0.736 (0.028) | 0.741 (0.027) | 0.736 (0.026) |
|  |  | **80** | 0.724 (0.035) | 0.719 (0.028) | 0.725 (0.032) | 0.711 (0.030) |
|  |  | **40** | 0.715 (0.028) | 0.697 (0.029) | 0.714 (0.030) | 0.676 (0.025) |
|  |  | **30** | 0.699 (0.052) | 0.674 (0.044) | 0.701 (0.052) | 0.661 (0.044) |
|  |  | **20** | 0.691 (0.029) | 0.630 (0.036) | 0.688 (0.027) | 0.634 (0.029) |
|  |  | **10** | 0.650 (0.044) | 0.574 (0.046) | 0.646 (0.044) | 0.606 (0.048) |
|  | **1000** | **138** | 0.610 (0.057) | 0.667 (0.054) | 0.678 (0.053) | 0.639 (0.058) |
|  |  | **80** | 0.588 (0.068) | 0.648 (0.069) | 0.641 (0.069) | 0.600 (0.067) |
|  |  | **40** | 0.644 (0.072) | 0.658 (0.068) | 0.670 (0.073) | 0.660 (0.070) |
|  |  | **30** | 0.639 (0.057) | 0.632 (0.058) | 0.645 (0.061) | 0.602 (0.067) |
|  |  | **20** | 0.617 (0.070) | 0.588 (0.055) | 0.613 (0.076) | 0.566 (0.063) |
|  |  | **10** | 0.631 (0.080) | 0.538 (0.076) | 0.628 (0.085) | 0.558 (0.072) |

RLR=Regularized Logistic Regression. RF=Random Forest. MLP=Multilayer Perceptron. XGBoost=eXtreme Gradient Boosting. SD=Standard Deviation

## Average AUCs of classifiers – test size 0.2 (Table A23)

**Table A23:**

**Average AUCs of classifiers for 12-month mortality prediction on balanced data set (test size 0.2)**

| **Test Size** | **Sample Size** | **Number of features** | **Average AUC (SD)** | | | |
| --- | --- | --- | --- | --- | --- | --- |
|  |  |  | **RLR** | **RF** | **MLP** | **XGBoost** |
| **0.2** | **95042** | **138** | 0.757 (0.003) | 0.754 (0.003) | 0.756 (0.003) | 0.765 (0.003) |
|  |  | **80** | 0.741 (0.009) | 0.737 (0.011) | 0.741 (0.011) | 0.743 (0.011) |
|  |  | **40** | 0.721 (0.013) | 0.700 (0.018) | 0.721 (0.012) | 0.717 (0.013) |
|  |  | **30** | 0.700 (0.015) | 0.658 (0.024) | 0.702 (0.016) | 0.695 (0.016) |
|  |  | **20** | 0.696 (0.017) | 0.625 (0.027) | 0.695 (0.017) | 0.687 (0.017) |
|  |  | **10** | 0.670 (0.025) | 0.617 (0.022) | 0.668 (0.024) | 0.657 (0.024) |
|  | **16000** | **138** | 0.747 (0.010) | 0.742 (0.010) | 0.748 (0.011) | 0.739 (0.010) |
|  |  | **80** | 0.734 (0.013) | 0.727 (0.014) | 0.732 (0.013) | 0.716 (0.014) |
|  |  | **40** | 0.720 (0.016) | 0.696 (0.021) | 0.716 (0.018) | 0.689 (0.016) |
|  |  | **30** | 0.710 (0.011) | 0.669 (0.023) | 0.708 (0.013) | 0.672 (0.020) |
|  |  | **20** | 0.702 (0.011) | 0.623 (0.022) | 0.699 (0.013) | 0.659 (0.018) |
|  |  | **10** | 0.657 (0.039) | 0.582 (0.032) | 0.655 (0.039) | 0.624 (0.044) |
|  | **4000** | **138** | 0.723 (0.018) | 0.736 (0.019) | 0.742 (0.019) | 0.733 (0.019) |
|  |  | **80** | 0.724 (0.022) | 0.727 (0.022) | 0.731 (0.026) | 0.710 (0.025) |
|  |  | **40** | 0.713 (0.027) | 0.701 (0.026) | 0.716 (0.027) | 0.689 (0.028) |
|  |  | **30** | 0.703 (0.024) | 0.679 (0.030) | 0.696 (0.026) | 0.663 (0.032) |
|  |  | **20** | 0.698 (0.031) | 0.628 (0.034) | 0.692 (0.032) | 0.641 (0.026) |
|  |  | **10** | 0.656 (0.040) | 0.578 (0.032) | 0.654 (0.039) | 0.606 (0.037) |
|  | **1000** | **138** | 0.655 (0.048) | 0.745 (0.033) | 0.737 (0.042) | 0.737 (0.041) |
|  |  | **80** | 0.660 (0.059) | 0.714 (0.039) | 0.719 (0.038) | 0.707 (0.045) |
|  |  | **40** | 0.668 (0.056) | 0.696 (0.070) | 0.698 (0.064) | 0.671 (0.061) |
|  |  | **30** | 0.679 (0.063) | 0.663 (0.060) | 0.683 (0.058) | 0.641 (0.047) |
|  |  | **20** | 0.681 (0.053) | 0.631 (0.071) | 0.688 (0.050) | 0.634 (0.055) |
|  |  | **10** | 0.651 (0.065) | 0.571 (0.056) | 0.648 (0.060) | 0.609 (0.053) |

Regularized LR=Regularized Logistic Regression. RF=Random Forest. MLP=Multilayer Perceptron. XGBoost=eXtreme Gradient Boosting. SD=Standard Deviation

## Average AUCs of classifiers – test size 0.3 (Table A24)

**Table A24:**

**Average AUCs of classifiers for 12-month mortality prediction on balanced data set (test size 0.3)**

| **Test Size** | **Sample Size** | **Number of features** | **Average AUC (SD)** | | | |
| --- | --- | --- | --- | --- | --- | --- |
|  |  |  | **RLR** | **RF** | **MLP** | **XGBoost** |
| **0.3** | **95042** | **138** | 0.755 (0.004) | 0.752 (0.004) | 0.754 (0.003) | 0.762 (0.003) |
|  |  | **80** | 0.741 (0.006) | 0.737 (0.008) | 0.741 (0.008) | 0.744 (0.009) |
|  |  | **40** | 0.722 (0.010) | 0.700 (0.015) | 0.721 (0.011) | 0.716 (0.011) |
|  |  | **30** | 0.710 (0.009) | 0.665 (0.023) | 0.712 (0.011) | 0.702 (0.010) |
|  |  | **20** | 0.687 (0.027) | 0.609 (0.025) | 0.688 (0.025) | 0.676 (0.026) |
|  |  | **10** | 0.657 (0.036) | 0.604 (0.030) | 0.657 (0.035) | 0.645 (0.036) |
|  | **16000** | **138** | 0.762 (0.007) | 0.759 (0.008) | 0.760 (0.008) | 0.761 (0.007) |
|  |  | **80** | 0.749 (0.007) | 0.744 (0.010) | 0.748 (0.009) | 0.738 (0.010) |
|  |  | **40** | 0.725 (0.014) | 0.704 (0.018) | 0.725 (0.015) | 0.699 (0.014) |
|  |  | **30** | 0.718 (0.015) | 0.684 (0.021) | 0.715 (0.016) | 0.685 (0.015) |
|  |  | **20** | 0.706 (0.017) | 0.641 (0.029) | 0.705 (0.020) | 0.672 (0.019) |
|  |  | **10** | 0.666 (0.028) | 0.601 (0.030) | 0.669 (0.031) | 0.640 (0.033) |
|  | **4000** | **138** | 0.707 (0.011) | 0.728 (0.011) | 0.733 (0.014) | 0.719 (0.013) |
|  |  | **80** | 0.705 (0.019) | 0.708 (0.018) | 0.712 (0.019) | 0.691 (0.018) |
|  |  | **40** | 0.690 (0.016) | 0.667 (0.020) | 0.689 (0.017) | 0.644 (0.018) |
|  |  | **30** | 0.692 (0.023) | 0.657 (0.019) | 0.690 (0.021) | 0.639 (0.026) |
|  |  | **20** | 0.672 (0.027) | 0.612 (0.032) | 0.671 (0.023) | 0.617 (0.028) |
|  |  | **10** | 0.654 (0.029) | 0.565 (0.032) | 0.648 (0.033) | 0.606 (0.031) |
|  | **1000** | **138** | 0.645 (0.041) | 0.725 (0.031) | 0.690 (0.029) | 0.718 (0.031) |
|  |  | **80** | 0.684 (0.028) | 0.702 (0.036) | 0.695 (0.035) | 0.694 (0.022) |
|  |  | **40** | 0.691 (0.051) | 0.676 (0.048) | 0.680 (0.037) | 0.670 (0.046) |
|  |  | **30** | 0.681 (0.039) | 0.662 (0.041) | 0.675 (0.040) | 0.644 (0.037) |
|  |  | **20** | 0.670 (0.044) | 0.632 (0.055) | 0.671 (0.045) | 0.628 (0.056) |
|  |  | **10** | 0.621 (0.070) | 0.554 (0.056) | 0.616 (0.067) | 0.579 (0.062) |

RLR=Regularized Logistic Regression. RF=Random Forest. MLP=Multilayer Perceptron. XGBoost=eXtreme Gradient Boosting. SD=Standard Deviation

## Average sensitivities and specificities of classifiers – test size 0.1 (Table A25)

**Table A25: Average sensitivities and specificities of classifiers for 12-month mortality prediction on balanced data set (test size 0.1)**

| **Test Size** | **Sample Size** | **Number of features** | **Average Sensitivity (SD)** | | | | **Average Specificity (SD)** | | | |
| --- | --- | --- | --- | --- | --- | --- | --- | --- | --- | --- |
|  |  |  | **RLR** | **RF** | **MLP** | **XGBoost** | **RLR** | **RF** | **MLP** | **XGBoost** |
| **0.1** | **95042** | **138** | 0.678 (0.013) | 0.214 (0.008) | 0.764 (0.031) | 0.596 (0.012) | 0.708 (0.005) | 0.955 (0.003) | 0.614 (0.033) | 0.786 (0.005) |
|  |  | **80** | 0.665 (0.013) | 0.203 (0.016) | 0.717 (0.035) | 0.589 (0.014) | 0.698 (0.009) | 0.953 (0.004) | 0.648 (0.032) | 0.769 (0.006) |
|  |  | **40** | 0.655 (0.018) | 0.198 (0.032) | 0.616 (0.055) | 0.588 (0.015) | 0.674 (0.018) | 0.937 (0.009) | 0.708 (0.049) | 0.731 (0.014) |
|  |  | **30** | 0.648 (0.020) | 0.221 (0.038) | 0.559 (0.084) | 0.584 (0.016) | 0.669 (0.021) | 0.904 (0.022) | 0.740 (0.059) | 0.715 (0.014) |
|  |  | **20** | 0.634 (0.025) | 0.285 (0.051) | 0.580 (0.070) | 0.591 (0.019) | 0.657 (0.023) | 0.836 (0.034) | 0.706 (0.059) | 0.693 (0.016) |
|  |  | **10** | 0.597 (0.082) | 0.483 (0.141) | 0.509 (0.112) | 0.590 (0.063) | 0.634 (0.059) | 0.684 (0.081) | 0.704 (0.091) | 0.633 (0.046) |
|  | **16000** | **138** | 0.652 (0.021) | 0.188 (0.025) | 0.774 (0.033) | 0.442 (0.030) | 0.714 (0.012) | 0.958 (0.007) | 0.594 (0.044) | 0.863 (0.009) |
|  |  | **80** | 0.650 (0.023) | 0.179 (0.028) | 0.754 (0.047) | 0.436 (0.032) | 0.701 (0.014) | 0.958 (0.005) | 0.599 (0.050) | 0.847 (0.009) |
|  |  | **40** | 0.647 (0.033) | 0.194 (0.045) | 0.711 (0.044) | 0.458 (0.033) | 0.683 (0.020) | 0.941 (0.013) | 0.613 (0.039) | 0.800 (0.020) |
|  |  | **30** | 0.631 (0.042) | 0.193 (0.037) | 0.650 (0.073) | 0.458 (0.038) | 0.677 (0.026) | 0.916 (0.019) | 0.651 (0.049) | 0.774 (0.017) |
|  |  | **20** | 0.620 (0.052) | 0.255 (0.058) | 0.641 (0.072) | 0.491 (0.037) | 0.667 (0.046) | 0.848 (0.044) | 0.646 (0.063) | 0.742 (0.028) |
|  |  | **10** | 0.597 (0.094) | 0.496 (0.078) | 0.508 (0.161) | 0.542 (0.068) | 0.645 (0.079) | 0.675 (0.057) | 0.708 (0.130) | 0.665 (0.047) |
|  | **4000** | **138** | 0.612 (0.045) | 0.148 (0.043) | 0.707 (0.061) | 0.390 (0.048) | 0.715 (0.033) | 0.965 (0.012) | 0.648 (0.052) | 0.894 (0.016) |
|  |  | **80** | 0.615 (0.053) | 0.163 (0.052) | 0.696 (0.074) | 0.374 (0.049) | 0.706 (0.027) | 0.958 (0.011) | 0.644 (0.059) | 0.881 (0.016) |
|  |  | **40** | 0.639 (0.044) | 0.170 (0.059) | 0.689 (0.076) | 0.366 (0.061) | 0.670 (0.029) | 0.948 (0.014) | 0.615 (0.064) | 0.842 (0.025) |
|  |  | **30** | 0.611 (0.071) | 0.194 (0.065) | 0.646 (0.098) | 0.401 (0.076) | 0.678 (0.040) | 0.927 (0.019) | 0.658 (0.060) | 0.815 (0.025) |
|  |  | **20** | 0.588 (0.072) | 0.230 (0.071) | 0.632 (0.078) | 0.394 (0.062) | 0.680 (0.035) | 0.875 (0.030) | 0.631 (0.056) | 0.780 (0.031) |
|  |  | **10** | 0.586 (0.087) | 0.420 (0.107) | 0.544 (0.101) | 0.469 (0.082) | 0.629 (0.071) | 0.705 (0.075) | 0.652 (0.104) | 0.697 (0.054) |
|  | **1000** | **138** | 0.447 (0.123) | 0.079 (0.064) | 0.598 (0.072) | 0.245 (0.098) | 0.698 (0.041) | 0.938 (0.028) | 0.660 (0.059) | 0.849 (0.031) |
|  |  | **80** | 0.478 (0.097) | 0.108 (0.079) | 0.560 (0.099) | 0.300 (0.105) | 0.631 (0.051) | 0.951 (0.023) | 0.636 (0.060) | 0.824 (0.033) |
|  |  | **40** | 0.574 (0.093) | 0.144 (0.050) | 0.653 (0.132) | 0.385 (0.091) | 0.657 (0.037) | 0.927 (0.032) | 0.633 (0.045) | 0.823 (0.034) |
|  |  | **30** | 0.584 (0.108) | 0.157 (0.090) | 0.658 (0.105) | 0.342 (0.090) | 0.619 (0.058) | 0.919 (0.030) | 0.551 (0.086) | 0.780 (0.042) |
|  |  | **20** | 0.532 (0.072) | 0.196 (0.094) | 0.603 (0.127) | 0.329 (0.081) | 0.639 (0.069) | 0.877 (0.037) | 0.551 (0.111) | 0.745 (0.054) |
|  |  | **10** | 0.563 (0.124) | 0.273 (0.098) | 0.647 (0.119) | 0.375 (0.107) | 0.644 (0.087) | 0.785 (0.070) | 0.543 (0.147) | 0.714 (0.064) |

## Average sensitivities and specificities of classifiers – test size 0.2 (Table A26)

**Table A26: Average sensitivities and specificities of classifiers for 12-month mortality prediction on balanced data set (test size 0.2)**

| **Test Size** | **Sample Size** | **Number of features** | **Average Sensitivity (SD)** | | | | **Average Specificity (SD)** | | | |
| --- | --- | --- | --- | --- | --- | --- | --- | --- | --- | --- |
|  |  |  | **RLR** | **RF** | **MLP** | **XGBoost** | **RLR** | **RF** | **MLP** | **XGBoost** |
| **0.2** | **95042** | **138** | 0.674 (0.005) | 0.207 (0.008) | 0.775 (0.032) | 0.586 (0.009) | 0.709 (0.004) | 0.955 (0.002) | 0.598 (0.037) | 0.790 (0.004) |
|  |  | **80** | 0.664 (0.015) | 0.198 (0.024) | 0.731 (0.042) | 0.579 (0.013) | 0.694 (0.011) | 0.953 (0.004) | 0.622 (0.040) | 0.769 (0.008) |
|  |  | **40** | 0.653 (0.020) | 0.197 (0.030) | 0.615 (0.074) | 0.578 (0.019) | 0.675 (0.016) | 0.936 (0.011) | 0.704 (0.068) | 0.734 (0.011) |
|  |  | **30** | 0.631 (0.026) | 0.208 (0.038) | 0.568 (0.082) | 0.568 (0.026) | 0.666 (0.024) | 0.903 (0.027) | 0.718 (0.068) | 0.717 (0.017) |
|  |  | **20** | 0.635 (0.032) | 0.271 (0.063) | 0.550 (0.059) | 0.581 (0.022) | 0.656 (0.026) | 0.844 (0.047) | 0.726 (0.050) | 0.691 (0.018) |
|  |  | **10** | 0.607 (0.046) | 0.516 (0.044) | 0.523 (0.140) | 0.585 (0.033) | 0.645 (0.042) | 0.677 (0.032) | 0.708 (0.092) | 0.650 (0.023) |
|  | **16000** | **138** | 0.670 (0.021) | 0.163 (0.011) | 0.784 (0.029) | 0.407 (0.016) | 0.705 (0.010) | 0.964 (0.005) | 0.575 (0.039) | 0.869 (0.005) |
|  |  | **80** | 0.649 (0.025) | 0.159 (0.021) | 0.749 (0.049) | 0.403 (0.024) | 0.695 (0.015) | 0.962 (0.006) | 0.589 (0.042) | 0.851 (0.009) |
|  |  | **40** | 0.647 (0.034) | 0.175 (0.028) | 0.685 (0.059) | 0.443 (0.027) | 0.681 (0.023) | 0.945 (0.010) | 0.634 (0.043) | 0.804 (0.020) |
|  |  | **30** | 0.636 (0.025) | 0.182 (0.036) | 0.678 (0.060) | 0.446 (0.037) | 0.675 (0.020) | 0.922 (0.018) | 0.625 (0.056) | 0.782 (0.017) |
|  |  | **20** | 0.647 (0.031) | 0.237 (0.060) | 0.623 (0.102) | 0.487 (0.044) | 0.654 (0.028) | 0.857 (0.040) | 0.664 (0.082) | 0.737 (0.021) |
|  |  | **10** | 0.605 (0.080) | 0.423 (0.103) | 0.538 (0.138) | 0.516 (0.082) | 0.629 (0.064) | 0.713 (0.069) | 0.678 (0.087) | 0.675 (0.051) |
|  | **4000** | **138** | 0.611 (0.029) | 0.187 (0.024) | 0.689 (0.063) | 0.358 (0.027) | 0.723 (0.017) | 0.957 (0.008) | 0.664 (0.059) | 0.893 (0.010) |
|  |  | **80** | 0.617 (0.051) | 0.180 (0.035) | 0.698 (0.056) | 0.359 (0.039) | 0.714 (0.027) | 0.961 (0.009) | 0.634 (0.062) | 0.887 (0.015) |
|  |  | **40** | 0.630 (0.056) | 0.191 (0.042) | 0.690 (0.074) | 0.386 (0.042) | 0.687 (0.024) | 0.938 (0.017) | 0.630 (0.058) | 0.847 (0.020) |
|  |  | **30** | 0.625 (0.036) | 0.200 (0.039) | 0.667 (0.054) | 0.388 (0.055) | 0.679 (0.019) | 0.926 (0.016) | 0.615 (0.043) | 0.818 (0.021) |
|  |  | **20** | 0.622 (0.052) | 0.263 (0.049) | 0.654 (0.090) | 0.443 (0.057) | 0.667 (0.028) | 0.849 (0.042) | 0.620 (0.069) | 0.755 (0.029) |
|  |  | **10** | 0.583 (0.072) | 0.402 (0.098) | 0.580 (0.071) | 0.477 (0.078) | 0.648 (0.068) | 0.726 (0.065) | 0.645 (0.072) | 0.688 (0.051) |
|  | **1000** | **138** | 0.499 (0.096) | 0.211 (0.068) | 0.604 (0.101) | 0.393 (0.082) | 0.734 (0.033) | 0.959 (0.015) | 0.745 (0.046) | 0.898 (0.024) |
|  |  | **80** | 0.517 (0.106) | 0.200 (0.059) | 0.621 (0.067) | 0.381 (0.071) | 0.717 (0.038) | 0.955 (0.022) | 0.684 (0.036) | 0.880 (0.029) |
|  |  | **40** | 0.556 (0.088) | 0.216 (0.067) | 0.637 (0.091) | 0.385 (0.100) | 0.700 (0.033) | 0.940 (0.019) | 0.650 (0.046) | 0.846 (0.022) |
|  |  | **30** | 0.589 (0.086) | 0.203 (0.070) | 0.653 (0.069) | 0.380 (0.099) | 0.689 (0.038) | 0.919 (0.032) | 0.610 (0.065) | 0.799 (0.036) |
|  |  | **20** | 0.580 (0.097) | 0.250 (0.086) | 0.625 (0.103) | 0.413 (0.085) | 0.684 (0.049) | 0.876 (0.040) | 0.628 (0.056) | 0.776 (0.034) |
|  |  | **10** | 0.560 (0.082) | 0.352 (0.079) | 0.613 (0.068) | 0.456 (0.074) | 0.663 (0.084) | 0.754 (0.076) | 0.613 (0.107) | 0.711 (0.053) |

## Average sensitivities and specificities of classifiers – test size 0.3 (Table A27)

**Table A27: Average sensitivities and specificities of classifiers for 12-month mortality prediction on balanced data set (test size 0.3)**

| **Test Size** | **Sample Size** | **Number of features** | **Average Sensitivity (SD)** | | | | **Average Specificity (SD)** | | | |
| --- | --- | --- | --- | --- | --- | --- | --- | --- | --- | --- |
|  |  |  | **RLR** | **RF** | **MLP** | **XGBoost** | **RLR** | **RF** | **MLP** | **XGBoost** |
| **0.3** | **95042** | **138** | 0.671 (0.005) | 0.207 (0.005) | 0.768 (0.034) | 0.572 (0.006) | 0.709 (0.004) | 0.954 (0.002) | 0.603 (0.039) | 0.798 (0.003) |
|  |  | **80** | 0.664 (0.008) | 0.197 (0.018) | 0.724 (0.043) | 0.570 (0.014) | 0.693 (0.012) | 0.953 (0.003) | 0.631 (0.041) | 0.777 (0.007) |
|  |  | **40** | 0.649 (0.012) | 0.198 (0.024) | 0.629 (0.058) | 0.572 (0.012) | 0.680 (0.017) | 0.936 (0.009) | 0.692 (0.050) | 0.740 (0.014) |
|  |  | **30** | 0.628 (0.027) | 0.229 (0.039) | 0.620 (0.099) | 0.564 (0.015) | 0.680 (0.017) | 0.900 (0.029) | 0.686 (0.078) | 0.726 (0.014) |
|  |  | **20** | 0.620 (0.044) | 0.306 (0.063) | 0.536 (0.099) | 0.566 (0.041) | 0.657 (0.024) | 0.812 (0.046) | 0.721 (0.086) | 0.691 (0.020) |
|  |  | **10** | 0.582 (0.056) | 0.470 (0.085) | 0.530 (0.095) | 0.551 (0.051) | 0.651 (0.055) | 0.693 (0.064) | 0.696 (0.073) | 0.666 (0.044) |
|  | **16000** | **138** | 0.672 (0.012) | 0.215 (0.014) | 0.783 (0.032) | 0.444 (0.010) | 0.718 (0.006) | 0.952 (0.005) | 0.596 (0.041) | 0.866 (0.006) |
|  |  | **80** | 0.658 (0.015) | 0.210 (0.021) | 0.763 (0.028) | 0.454 (0.017) | 0.710 (0.012) | 0.949 (0.004) | 0.600 (0.032) | 0.844 (0.008) |
|  |  | **40** | 0.650 (0.024) | 0.208 (0.032) | 0.726 (0.033) | 0.460 (0.023) | 0.684 (0.019) | 0.933 (0.008) | 0.602 (0.043) | 0.800 (0.017) |
|  |  | **30** | 0.639 (0.025) | 0.215 (0.041) | 0.693 (0.062) | 0.462 (0.023) | 0.684 (0.018) | 0.916 (0.015) | 0.620 (0.059) | 0.785 (0.015) |
|  |  | **20** | 0.634 (0.032) | 0.281 (0.049) | 0.644 (0.082) | 0.499 (0.032) | 0.673 (0.036) | 0.851 (0.040) | 0.654 (0.069) | 0.741 (0.027) |
|  |  | **10** | 0.580 (0.062) | 0.447 (0.082) | 0.575 (0.098) | 0.528 (0.069) | 0.668 (0.052) | 0.720 (0.070) | 0.672 (0.071) | 0.688 (0.062) |
|  | **4000** | **138** | 0.600 (0.025) | 0.136 (0.023) | 0.726 (0.050) | 0.326 (0.031) | 0.697 (0.011) | 0.964 (0.006) | 0.622 (0.054) | 0.894 (0.014) |
|  |  | **80** | 0.623 (0.036) | 0.145 (0.024) | 0.705 (0.050) | 0.335 (0.030) | 0.677 (0.013) | 0.959 (0.006) | 0.600 (0.032) | 0.870 (0.009) |
|  |  | **40** | 0.616 (0.038) | 0.134 (0.024) | 0.660 (0.042) | 0.328 (0.024) | 0.659 (0.026) | 0.944 (0.007) | 0.611 (0.044) | 0.834 (0.024) |
|  |  | **30** | 0.615 (0.049) | 0.165 (0.034) | 0.671 (0.074) | 0.356 (0.043) | 0.660 (0.031) | 0.924 (0.016) | 0.598 (0.072) | 0.814 (0.017) |
|  |  | **20** | 0.603 (0.066) | 0.235 (0.042) | 0.637 (0.062) | 0.403 (0.046) | 0.645 (0.030) | 0.852 (0.038) | 0.611 (0.054) | 0.757 (0.032) |
|  |  | **10** | 0.577 (0.105) | 0.384 (0.106) | 0.575 (0.127) | 0.459 (0.095) | 0.646 (0.065) | 0.722 (0.066) | 0.635 (0.102) | 0.689 (0.052) |
|  | **1000** | **138** | 0.456 (0.072) | 0.121 (0.044) | 0.579 (0.078) | 0.316 (0.071) | 0.751 (0.025) | 0.974 (0.012) | 0.707 (0.046) | 0.898 (0.017) |
|  |  | **80** | 0.531 (0.052) | 0.118 (0.033) | 0.597 (0.072) | 0.322 (0.067) | 0.731 (0.028) | 0.974 (0.012) | 0.704 (0.040) | 0.882 (0.023) |
|  |  | **40** | 0.588 (0.072) | 0.143 (0.052) | 0.643 (0.060) | 0.331 (0.066) | 0.699 (0.033) | 0.953 (0.012) | 0.615 (0.050) | 0.847 (0.030) |
|  |  | **30** | 0.587 (0.084) | 0.157 (0.046) | 0.657 (0.105) | 0.359 (0.067) | 0.685 (0.045) | 0.940 (0.021) | 0.595 (0.095) | 0.831 (0.038) |
|  |  | **20** | 0.582 (0.071) | 0.183 (0.072) | 0.620 (0.080) | 0.381 (0.076) | 0.679 (0.034) | 0.892 (0.040) | 0.631 (0.043) | 0.785 (0.031) |
|  |  | **10** | 0.555 (0.117) | 0.295 (0.097) | 0.620 (0.163) | 0.405 (0.105) | 0.633 (0.059) | 0.780 (0.078) | 0.556 (0.147) | 0.700 (0.058) |

## Average F1-scores of classifiers – test size 0.1 (Table A28)

**Table A28:**

**Average F1-scores of classifiers for 12-month mortality prediction on balanced data set (test size 0.1)**

| **Test Size** | **Sample Size** | **Number of features** | **Average F1-Score (SD)** | | | |
| --- | --- | --- | --- | --- | --- | --- |
|  |  |  | **RLR** | **RF** | **MLP** | **XGBosst** |
| **0.1** | **95042** | **138** | 0.474 (0.008) | 0.306 (0.011) | 0.459 (0.007) | 0.483 (0.009) |
|  |  | **80** | 0.460 (0.008) | 0.291 (0.019) | 0.455 (0.008) | 0.466 (0.009) |
|  |  | **40** | 0.442 (0.008) | 0.272 (0.034) | 0.442 (0.010) | 0.441 (0.011) |
|  |  | **30** | 0.432 (0.011) | 0.273 (0.029) | 0.425 (0.018) | 0.426 (0.010) |
|  |  | **20** | 0.419 (0.013) | 0.290 (0.029) | 0.418 (0.015) | 0.416 (0.013) |
|  |  | **10** | 0.386 (0.027) | 0.341 (0.057) | 0.373 (0.040) | 0.383 (0.024) |
|  | **16000** | **138** | 0.458 (0.018) | 0.274 (0.030) | 0.447 (0.018) | 0.438 (0.023) |
|  |  | **80** | 0.451 (0.017) | 0.264 (0.035) | 0.443 (0.018) | 0.422 (0.029) |
|  |  | **40** | 0.441 (0.022) | 0.269 (0.053) | 0.434 (0.024) | 0.403 (0.019) |
|  |  | **30** | 0.424 (0.020) | 0.249 (0.037) | 0.418 (0.022) | 0.381 (0.026) |
|  |  | **20** | 0.414 (0.021) | 0.269 (0.032) | 0.413 (0.021) | 0.384 (0.018) |
|  |  | **10** | 0.389 (0.030) | 0.349 (0.034) | 0.366 (0.050) | 0.370 (0.029) |
|  | **4000** | **138** | 0.440 (0.044) | 0.227 (0.055) | 0.449 (0.040) | 0.425 (0.040) |
|  |  | **80** | 0.434 (0.041) | 0.241 (0.068) | 0.440 (0.038) | 0.400 (0.051) |
|  |  | **40** | 0.419 (0.037) | 0.241 (0.072) | 0.414 (0.042) | 0.358 (0.051) |
|  |  | **30** | 0.413 (0.050) | 0.255 (0.073) | 0.419 (0.053) | 0.367 (0.060) |
|  |  | **20** | 0.405 (0.035) | 0.261 (0.063) | 0.403 (0.035) | 0.343 (0.039) |
|  |  | **10** | 0.372 (0.033) | 0.314 (0.051) | 0.363 (0.041) | 0.342 (0.047) |
|  | **1000** | **138** | 0.346 (0.103) | 0.114 (0.081) | 0.415 (0.070) | 0.264 (0.099) |
|  |  | **80** | 0.327 (0.087) | 0.157 (0.104) | 0.374 (0.083) | 0.295 (0.088) |
|  |  | **40** | 0.411 (0.075) | 0.204 (0.062) | 0.436 (0.074) | 0.380 (0.074) |
|  |  | **30** | 0.391 (0.079) | 0.210 (0.120) | 0.400 (0.085) | 0.316 (0.078) |
|  |  | **20** | 0.376 (0.065) | 0.233 (0.096) | 0.373 (0.056) | 0.291 (0.070) |
|  |  | **10** | 0.384 (0.082) | 0.256 (0.084) | 0.383 (0.064) | 0.303 (0.097) |

RLR=Regularized Logistic Regression. RF=Random Forest. MLP=Multilayer Perceptron. XGBoost=eXtreme Gradient Boosting. SD=Standard Deviation

## Average F1-scores of classifiers – test size 0.2 (Table A29)

**Table A29:**

**Average F1-scores of classifiers for 12-month mortality prediction on balanced data set (test size 0.2)**

| **Test Size** | **Sample Size** | **Number of features** | **Average F1-Score (SD)** | | | |
| --- | --- | --- | --- | --- | --- | --- |
|  |  |  | **RLR** | **RF** | **MLP** | **XGBosst** |
| **0.2** | **95042** | **138** | 0.473 (0.005) | 0.298 (0.009) | 0.457 (0.007) | 0.482 (0.006) |
|  |  | **80** | 0.457 (0.009) | 0.285 (0.028) | 0.448 (0.009) | 0.460 (0.012) |
|  |  | **40** | 0.440 (0.012) | 0.271 (0.032) | 0.437 (0.014) | 0.435 (0.013) |
|  |  | **30** | 0.424 (0.013) | 0.259 (0.032) | 0.419 (0.018) | 0.419 (0.017) |
|  |  | **20** | 0.419 (0.015) | 0.281 (0.033) | 0.413 (0.017) | 0.410 (0.016) |
|  |  | **10** | 0.397 (0.020) | 0.364 (0.020) | 0.375 (0.073) | 0.388 (0.021) |
|  | **16000** | **138** | 0.459 (0.013) | 0.247 (0.015) | 0.439 (0.016) | 0.415 (0.012) |
|  |  | **80** | 0.440 (0.016) | 0.240 (0.026) | 0.428 (0.013) | 0.395 (0.019) |
|  |  | **40** | 0.430 (0.016) | 0.247 (0.035) | 0.422 (0.017) | 0.389 (0.017) |
|  |  | **30** | 0.419 (0.013) | 0.239 (0.037) | 0.412 (0.016) | 0.374 (0.023) |
|  |  | **20** | 0.414 (0.009) | 0.253 (0.033) | 0.406 (0.020) | 0.372 (0.019) |
|  |  | **10** | 0.380 (0.037) | 0.316 (0.040) | 0.364 (0.053) | 0.354 (0.040) |
|  | **4000** | **138** | 0.450 (0.022) | 0.275 (0.030) | 0.456 (0.026) | 0.401 (0.022) |
|  |  | **80** | 0.451 (0.036) | 0.268 (0.044) | 0.447 (0.034) | 0.398 (0.036) |
|  |  | **40** | 0.441 (0.035) | 0.264 (0.045) | 0.440 (0.026) | 0.389 (0.041) |
|  |  | **30** | 0.430 (0.023) | 0.266 (0.044) | 0.417 (0.026) | 0.367 (0.040) |
|  |  | **20** | 0.419 (0.034) | 0.279 (0.035) | 0.409 (0.034) | 0.363 (0.035) |
|  |  | **10** | 0.395 (0.034) | 0.321 (0.047) | 0.392 (0.034) | 0.353 (0.041) |
|  | **1000** | **138** | 0.374 (0.058) | 0.297 (0.078) | 0.444 (0.058) | 0.424 (0.057) |
|  |  | **80** | 0.388 (0.071) | 0.286 (0.074) | 0.429 (0.047) | 0.407 (0.067) |
|  |  | **40** | 0.410 (0.069) | 0.296 (0.085) | 0.426 (0.059) | 0.386 (0.085) |
|  |  | **30** | 0.416 (0.065) | 0.264 (0.084) | 0.408 (0.054) | 0.346 (0.085) |
|  |  | **20** | 0.406 (0.057) | 0.282 (0.078) | 0.400 (0.049) | 0.355 (0.056) |
|  |  | **10** | 0.386 (0.068) | 0.297 (0.044) | 0.391 (0.061) | 0.346 (0.052) |

RLR=Regularized Logistic Regression. RF=Random Forest. MLP=Multilayer Perceptron. XGBoost=eXtreme Gradient Boosting. SD=Standard Deviation

## Average F1-scores of classifiers – test size 0.3 (Table A30)

**Table A30:**

**Average F1-scores of classifiers for 12-month mortality prediction on balanced data set (test size 0.3)**

| **Test Size** | **Sample Size** | **Number of features** | **Average F1-Score (SD)** | | | |
| --- | --- | --- | --- | --- | --- | --- |
|  |  |  | **RLR** | **RF** | **MLP** | **XGBosst** |
| **0.3** | **95042** | **138** | 0.470 (0.004) | 0.298 (0.005) | 0.455 (0.008) | 0.478 (0.005) |
|  |  | **80** | 0.456 (0.007) | 0.284 (0.020) | 0.449 (0.007) | 0.460 (0.010) |
|  |  | **40** | 0.441 (0.009) | 0.271 (0.028) | 0.437 (0.011) | 0.436 (0.011) |
|  |  | **30** | 0.429 (0.010) | 0.278 (0.023) | 0.427 (0.016) | 0.422 (0.010) |
|  |  | **20** | 0.411 (0.022) | 0.293 (0.034) | 0.401 (0.021) | 0.401 (0.023) |
|  |  | **10** | 0.388 (0.027) | 0.344 (0.036) | 0.382 (0.035) | 0.379 (0.027) |
|  | **16000** | **138** | 0.478 (0.010) | 0.305 (0.014) | 0.459 (0.013) | 0.447 (0.009) |
|  |  | **80** | 0.467 (0.010) | 0.296 (0.024) | 0.453 (0.010) | 0.437 (0.013) |
|  |  | **40** | 0.447 (0.017) | 0.282 (0.036) | 0.439 (0.016) | 0.408 (0.018) |
|  |  | **30** | 0.441 (0.014) | 0.275 (0.039) | 0.433 (0.012) | 0.398 (0.017) |
|  |  | **20** | 0.431 (0.018) | 0.297 (0.027) | 0.425 (0.025) | 0.393 (0.018) |
|  |  | **10** | 0.398 (0.023) | 0.346 (0.023) | 0.396 (0.034) | 0.380 (0.026) |
|  | **4000** | **138** | 0.419 (0.018) | 0.210 (0.028) | 0.440 (0.021) | 0.368 (0.024) |
|  |  | **80** | 0.429 (0.020) | 0.221 (0.031) | 0.427 (0.017) | 0.361 (0.028) |
|  |  | **40** | 0.411 (0.022) | 0.197 (0.031) | 0.408 (0.018) | 0.328 (0.018) |
|  |  | **30** | 0.408 (0.024) | 0.222 (0.037) | 0.404 (0.020) | 0.335 (0.032) |
|  |  | **20** | 0.392 (0.033) | 0.253 (0.027) | 0.392 (0.029) | 0.335 (0.035) |
|  |  | **10** | 0.380 (0.037) | 0.300 (0.047) | 0.373 (0.037) | 0.334 (0.036) |
|  | **1000** | **138** | 0.350 (0.049) | 0.193 (0.065) | 0.396 (0.045) | 0.352 (0.068) |
|  |  | **80** | 0.377 (0.022) | 0.188 (0.046) | 0.396 (0.035) | 0.338 (0.049) |
|  |  | **40** | 0.405 (0.054) | 0.210 (0.070) | 0.387 (0.040) | 0.330 (0.057) |
|  |  | **30** | 0.384 (0.043) | 0.217 (0.059) | 0.372 (0.042) | 0.333 (0.050) |
|  |  | **20** | 0.382 (0.051) | 0.215 (0.078) | 0.376 (0.055) | 0.321 (0.058) |
|  |  | **10** | 0.334 (0.060) | 0.245 (0.043) | 0.329 (0.057) | 0.281 (0.050) |

RLR=Regularized Logistic Regression. RF=Random Forest. MLP=Multilayer Perceptron. XGBoost=eXtreme Gradient Boosting. SD=Standard Deviation

## Average accuracies and precisions of classifiers – test size 0.1 (Table A31)

**Table A31: Average accuracies and precisions of classifiers for 12-month mortality prediction on balanced data set (test size 0.1)**

| **Test Size** | **Sample Size** | **Number of features** | **Average Accuracy (SD)** | | | | **Average Precision (SD)** | | | |
| --- | --- | --- | --- | --- | --- | --- | --- | --- | --- | --- |
|  |  |  | **RLR** | **RF** | **MLP** | **XGBoost** | **RLR** | **RF** | **MLP** | **XGBoost** |
| **0.1** | **95042** | **138** | 0.702 (0.005) | 0.808 (0.004) | 0.644 (0.021) | 0.748 (0.005) | 0.364 (0.007) | 0.540 (0.018) | 0.329 (0.011) | 0.407 (0.009) |
|  |  | **80** | 0.692 (0.007) | 0.805 (0.005) | 0.662 (0.020) | 0.734 (0.006) | 0.351 (0.009) | 0.514 (0.026) | 0.334 (0.011) | 0.386 (0.008) |
|  |  | **40** | 0.670 (0.012) | 0.789 (0.008) | 0.690 (0.029) | 0.702 (0.010) | 0.334 (0.008) | 0.438 (0.034) | 0.347 (0.018) | 0.353 (0.013) |
|  |  | **30** | 0.664 (0.015) | 0.770 (0.013) | 0.704 (0.032) | 0.689 (0.011) | 0.325 (0.013) | 0.365 (0.032) | 0.349 (0.023) | 0.335 (0.011) |
|  |  | **20** | 0.652 (0.017) | 0.727 (0.020) | 0.681 (0.035) | 0.672 (0.013) | 0.314 (0.013) | 0.301 (0.028) | 0.330 (0.021) | 0.322 (0.012) |
|  |  | **10** | 0.627 (0.036) | 0.644 (0.041) | 0.666 (0.055) | 0.624 (0.029) | 0.287 (0.020) | 0.271 (0.024) | 0.303 (0.030) | 0.284 (0.019) |
|  | **16000** | **138** | 0.702 (0.010) | 0.809 (0.010) | 0.629 (0.030) | 0.781 (0.011) | 0.353 (0.019) | 0.515 (0.052) | 0.315 (0.018) | 0.436 (0.024) |
|  |  | **80** | 0.691 (0.012) | 0.806 (0.007) | 0.629 (0.033) | 0.767 (0.011) | 0.346 (0.016) | 0.506 (0.049) | 0.314 (0.019) | 0.409 (0.029) |
|  |  | **40** | 0.676 (0.016) | 0.793 (0.014) | 0.633 (0.028) | 0.732 (0.013) | 0.335 (0.022) | 0.447 (0.066) | 0.313 (0.022) | 0.361 (0.023) |
|  |  | **30** | 0.668 (0.016) | 0.776 (0.014) | 0.651 (0.028) | 0.713 (0.013) | 0.320 (0.018) | 0.360 (0.054) | 0.310 (0.015) | 0.328 (0.026) |
|  |  | **20** | 0.658 (0.030) | 0.733 (0.029) | 0.645 (0.039) | 0.693 (0.019) | 0.313 (0.025) | 0.295 (0.042) | 0.307 (0.023) | 0.316 (0.022) |
|  |  | **10** | 0.636 (0.049) | 0.640 (0.038) | 0.669 (0.076) | 0.641 (0.030) | 0.293 (0.030) | 0.271 (0.025) | 0.311 (0.044) | 0.282 (0.023) |
|  | **4000** | **138** | 0.694 (0.025) | 0.804 (0.022) | 0.659 (0.036) | 0.794 (0.018) | 0.346 (0.047) | 0.513 (0.094) | 0.332 (0.043) | 0.474 (0.056) |
|  |  | **80** | 0.688 (0.025) | 0.803 (0.015) | 0.655 (0.040) | 0.782 (0.017) | 0.337 (0.040) | 0.482 (0.115) | 0.324 (0.036) | 0.433 (0.064) |
|  |  | **40** | 0.664 (0.022) | 0.801 (0.021) | 0.630 (0.043) | 0.752 (0.021) | 0.313 (0.036) | 0.433 (0.087) | 0.298 (0.039) | 0.353 (0.054) |
|  |  | **30** | 0.665 (0.037) | 0.785 (0.022) | 0.656 (0.041) | 0.735 (0.025) | 0.313 (0.043) | 0.386 (0.089) | 0.313 (0.046) | 0.341 (0.057) |
|  |  | **20** | 0.661 (0.024) | 0.747 (0.030) | 0.631 (0.037) | 0.703 (0.026) | 0.311 (0.032) | 0.312 (0.064) | 0.298 (0.034) | 0.306 (0.037) |
|  |  | **10** | 0.620 (0.046) | 0.650 (0.052) | 0.630 (0.070) | 0.653 (0.040) | 0.276 (0.032) | 0.256 (0.042) | 0.280 (0.049) | 0.272 (0.041) |
|  | **1000** | **138** | 0.648 (0.043) | 0.754 (0.040) | 0.647 (0.047) | 0.720 (0.039) | 0.286 (0.091) | 0.261 (0.175) | 0.324 (0.075) | 0.301 (0.109) |
|  |  | **80** | 0.601 (0.051) | 0.777 (0.046) | 0.622 (0.052) | 0.717 (0.049) | 0.253 (0.081) | 0.329 (0.206) | 0.285 (0.076) | 0.298 (0.087) |
|  |  | **40** | 0.639 (0.044) | 0.753 (0.042) | 0.635 (0.036) | 0.726 (0.038) | 0.323 (0.070) | 0.384 (0.144) | 0.334 (0.066) | 0.382 (0.076) |
|  |  | **30** | 0.612 (0.042) | 0.754 (0.031) | 0.574 (0.066) | 0.685 (0.039) | 0.300 (0.078) | 0.343 (0.204) | 0.293 (0.081) | 0.302 (0.087) |
|  |  | **20** | 0.615 (0.056) | 0.728 (0.030) | 0.561 (0.069) | 0.653 (0.044) | 0.296 (0.074) | 0.307 (0.126) | 0.277 (0.058) | 0.269 (0.085) |
|  |  | **10** | 0.630 (0.071) | 0.679 (0.057) | 0.566 (0.109) | 0.645 (0.057) | 0.296 (0.071) | 0.257 (0.113) | 0.279 (0.061) | 0.260 (0.098) |

## Average accuracies and precisions of classifiers – test size 0.2 (Table A32)

**Table A32: Average accuracies and precisions of classifiers for 12-month mortality prediction on balanced data set (test size 0.2)**

| **Test Size** | **Sample Size** | **Number of features** | **Average Accuracy (SD)** | | | | **Average Precision (SD)** | | | | |
| --- | --- | --- | --- | --- | --- | --- | --- | --- | --- | --- | --- |
|  |  |  | **RLR** | **RF** | **MLP** | **XGBoost** | **RLR** | **RF** | **MLP** | **XGBoost** |  |
| **0.2** | **95042** | **138** | 0.702 (0.004) | 0.806 (0.003) | 0.633 (0.024) | 0.750 (0.003) | 0.365 (0.005) | 0.533 (0.011) | 0.325 (0.012) | 0.410 (0.006) |  |
|  |  | **80** | 0.688 (0.008) | 0.804 (0.003) | 0.644 (0.025) | 0.731 (0.008) | 0.348 (0.009) | 0.510 (0.018) | 0.324 (0.014) | 0.382 (0.012) |  |
|  |  | **40** | 0.671 (0.012) | 0.790 (0.007) | 0.687 (0.041) | 0.703 (0.009) | 0.332 (0.012) | 0.435 (0.039) | 0.344 (0.029) | 0.349 (0.012) |  |
|  |  | **30** | 0.659 (0.016) | 0.765 (0.017) | 0.688 (0.040) | 0.687 (0.013) | 0.319 (0.012) | 0.352 (0.039) | 0.337 (0.025) | 0.332 (0.015) |  |
|  |  | **20** | 0.652 (0.017) | 0.731 (0.026) | 0.691 (0.030) | 0.669 (0.014) | 0.313 (0.013) | 0.305 (0.030) | 0.334 (0.024) | 0.317 (0.016) |  |
|  |  | **10** | 0.637 (0.028) | 0.646 (0.021) | 0.671 (0.049) | 0.637 (0.018) | 0.296 (0.019) | 0.282 (0.017) | 0.313 (0.034) | 0.291 (0.017) |  |
|  | **16000** | **138** | 0.698 (0.008) | 0.810 (0.006) | 0.615 (0.027) | 0.780 (0.006) | 0.350 (0.012) | 0.516 (0.039) | 0.305 (0.018) | 0.424 (0.014) |  |
|  |  | **80** | 0.686 (0.011) | 0.809 (0.008) | 0.620 (0.025) | 0.765 (0.008) | 0.334 (0.014) | 0.496 (0.042) | 0.301 (0.014) | 0.388 (0.021) |  |
|  |  | **40** | 0.674 (0.016) | 0.798 (0.010) | 0.643 (0.026) | 0.735 (0.015) | 0.322 (0.014) | 0.426 (0.049) | 0.306 (0.018) | 0.348 (0.021) |  |
|  |  | **30** | 0.668 (0.013) | 0.782 (0.011) | 0.635 (0.036) | 0.718 (0.011) | 0.313 (0.013) | 0.354 (0.055) | 0.298 (0.017) | 0.322 (0.021) |  |
|  |  | **20** | 0.653 (0.018) | 0.739 (0.023) | 0.656 (0.047) | 0.690 (0.012) | 0.305 (0.011) | 0.283 (0.027) | 0.307 (0.030) | 0.302 (0.015) |  |
|  |  | **10** | 0.624 (0.043) | 0.658 (0.038) | 0.652 (0.048) | 0.645 (0.033) | 0.279 (0.033) | 0.257 (0.027) | 0.284 (0.029) | 0.272 (0.033) |  |
|  | **4000** | **138** | 0.700 (0.016) | 0.802 (0.013) | 0.669 (0.037) | 0.785 (0.013) | 0.357 (0.025) | 0.524 (0.060) | 0.343 (0.031) | 0.457 (0.032) |  |
|  |  | **80** | 0.695 (0.019) | 0.801 (0.017) | 0.647 (0.042) | 0.779 (0.012) | 0.356 (0.036) | 0.540 (0.074) | 0.331 (0.040) | 0.449 (0.054) |  |
|  |  | **40** | 0.676 (0.018) | 0.786 (0.014) | 0.643 (0.035) | 0.753 (0.017) | 0.340 (0.029) | 0.443 (0.058) | 0.325 (0.023) | 0.393 (0.049) |  |
|  |  | **30** | 0.668 (0.017) | 0.779 (0.019) | 0.625 (0.028) | 0.732 (0.019) | 0.329 (0.023) | 0.405 (0.061) | 0.305 (0.029) | 0.350 (0.039) |  |
|  |  | **20** | 0.658 (0.022) | 0.733 (0.029) | 0.627 (0.043) | 0.694 (0.021) | 0.316 (0.030) | 0.306 (0.042) | 0.300 (0.029) | 0.309 (0.027) |  |
|  |  | **10** | 0.635 (0.046) | 0.660 (0.036) | 0.632 (0.049) | 0.645 (0.033) | 0.301 (0.035) | 0.274 (0.030) | 0.299 (0.035) | 0.282 (0.032) |  |
|  | **1000** | **138** | 0.689 (0.029) | 0.817 (0.026) | 0.719 (0.025) | 0.802 (0.021) | 0.302 (0.052) | 0.544 (0.098) | 0.357 (0.062) | 0.474 (0.076) |  |
|  |  | **80** | 0.676 (0.037) | 0.803 (0.024) | 0.672 (0.028) | 0.779 (0.038) | 0.314 (0.066) | 0.539 (0.147) | 0.331 (0.048) | 0.445 (0.082) |  |
|  |  | **40** | 0.671 (0.031) | 0.790 (0.027) | 0.648 (0.036) | 0.751 (0.025) | 0.326 (0.061) | 0.484 (0.140) | 0.323 (0.053) | 0.392 (0.085) |  |
|  |  | **30** | 0.669 (0.037) | 0.775 (0.028) | 0.619 (0.052) | 0.715 (0.031) | 0.323 (0.056) | 0.397 (0.131) | 0.299 (0.048) | 0.322 (0.081) |  |
|  |  | **20** | 0.664 (0.040) | 0.750 (0.043) | 0.628 (0.039) | 0.703 (0.038) | 0.315 (0.048) | 0.336 (0.084) | 0.296 (0.039) | 0.314 (0.046) |  |
|  |  | **10** | 0.643 (0.066) | 0.672 (0.051) | 0.613 (0.083) | 0.660 (0.036) | 0.299 (0.067) | 0.269 (0.062) | 0.291 (0.062) | 0.284 (0.058) |  |

## Average accuracies and precisions of classifiers – test size 0.3 (Table A33)

**Table A33: Average accuracies and precisions of classifiers for 12-month mortality prediction on balanced data set (test size 0.3)**

| **Test Size** | **Sample Size** | **Number of features** | **Average Accuracy (SD)** | | | | **Average Precision (SD)** | | | |
| --- | --- | --- | --- | --- | --- | --- | --- | --- | --- | --- |
|  |  |  | **RLR** | **RF** | **MLP** | **XGBoost** | **RLR** | **RF** | **MLP** | **XGBoost** |
| **0.3** | **95042** | **138** | 0.702 (0.003) | 0.807 (0.002) | 0.636 (0.025) | 0.753 (0.003) | 0.362 (0.004) | 0.528 (0.010) | 0.324 (0.015) | 0.411 (0.005) |
|  |  | **80** | 0.687 (0.009) | 0.804 (0.003) | 0.649 (0.025) | 0.736 (0.007) | 0.347 (0.008) | 0.509 (0.019) | 0.326 (0.013) | 0.385 (0.010) |
|  |  | **40** | 0.674 (0.012) | 0.790 (0.007) | 0.680 (0.030) | 0.707 (0.011) | 0.334 (0.011) | 0.434 (0.038) | 0.338 (0.021) | 0.352 (0.013) |
|  |  | **30** | 0.670 (0.010) | 0.767 (0.016) | 0.673 (0.043) | 0.694 (0.010) | 0.326 (0.008) | 0.366 (0.038) | 0.333 (0.031) | 0.337 (0.011) |
|  |  | **20** | 0.649 (0.018) | 0.712 (0.027) | 0.685 (0.051) | 0.667 (0.015) | 0.307 (0.017) | 0.287 (0.022) | 0.329 (0.036) | 0.311 (0.017) |
|  |  | **10** | 0.637 (0.039) | 0.649 (0.040) | 0.663 (0.044) | 0.643 (0.033) | 0.293 (0.028) | 0.275 (0.025) | 0.303 (0.028) | 0.290 (0.025) |
|  | **16000** | **138** | 0.709 (0.005) | 0.806 (0.004) | 0.633 (0.027) | 0.782 (0.004) | 0.371 (0.011) | 0.526 (0.025) | 0.325 (0.015) | 0.450 (0.016) |
|  |  | **80** | 0.700 (0.009) | 0.801 (0.004) | 0.632 (0.021) | 0.767 (0.006) | 0.362 (0.011) | 0.505 (0.019) | 0.323 (0.012) | 0.421 (0.014) |
|  |  | **40** | 0.677 (0.014) | 0.788 (0.006) | 0.627 (0.030) | 0.732 (0.013) | 0.341 (0.015) | 0.438 (0.037) | 0.315 (0.018) | 0.367 (0.022) |
|  |  | **30** | 0.675 (0.014) | 0.775 (0.010) | 0.635 (0.036) | 0.720 (0.013) | 0.337 (0.013) | 0.390 (0.034) | 0.316 (0.018) | 0.350 (0.017) |
|  |  | **20** | 0.665 (0.025) | 0.737 (0.024) | 0.652 (0.042) | 0.693 (0.019) | 0.327 (0.019) | 0.325 (0.035) | 0.320 (0.024) | 0.326 (0.020) |
|  |  | **10** | 0.650 (0.034) | 0.665 (0.042) | 0.653 (0.041) | 0.656 (0.040) | 0.305 (0.021) | 0.288 (0.023) | 0.308 (0.025) | 0.300 (0.026) |
|  | **4000** | **138** | 0.678 (0.009) | 0.804 (0.007) | 0.642 (0.036) | 0.784 (0.009) | 0.322 (0.018) | 0.477 (0.044) | 0.317 (0.023) | 0.426 (0.035) |
|  |  | **80** | 0.667 (0.012) | 0.796 (0.010) | 0.621 (0.020) | 0.762 (0.010) | 0.327 (0.017) | 0.471 (0.052) | 0.307 (0.015) | 0.393 (0.031) |
|  |  | **40** | 0.650 (0.019) | 0.783 (0.009) | 0.620 (0.030) | 0.734 (0.018) | 0.309 (0.020) | 0.370 (0.044) | 0.297 (0.020) | 0.331 (0.032) |
|  |  | **30** | 0.651 (0.021) | 0.776 (0.011) | 0.613 (0.045) | 0.725 (0.013) | 0.306 (0.020) | 0.348 (0.049) | 0.291 (0.022) | 0.318 (0.031) |
|  |  | **20** | 0.637 (0.018) | 0.732 (0.024) | 0.616 (0.036) | 0.688 (0.023) | 0.291 (0.024) | 0.282 (0.040) | 0.285 (0.026) | 0.288 (0.036) |
|  |  | **10** | 0.632 (0.035) | 0.655 (0.035) | 0.623 (0.060) | 0.644 (0.025) | 0.286 (0.023) | 0.252 (0.024) | 0.282 (0.025) | 0.266 (0.018) |
|  | **1000** | **138** | 0.698 (0.025) | 0.822 (0.014) | 0.684 (0.033) | 0.793 (0.022) | 0.286 (0.042) | 0.509 (0.144) | 0.303 (0.040) | 0.400 (0.065) |
|  |  | **80** | 0.696 (0.020) | 0.824 (0.018) | 0.685 (0.027) | 0.784 (0.016) | 0.294 (0.027) | 0.496 (0.113) | 0.299 (0.036) | 0.365 (0.051) |
|  |  | **40** | 0.679 (0.030) | 0.802 (0.023) | 0.621 (0.036) | 0.750 (0.030) | 0.310 (0.048) | 0.407 (0.117) | 0.278 (0.037) | 0.334 (0.068) |
|  |  | **30** | 0.667 (0.034) | 0.802 (0.022) | 0.606 (0.067) | 0.747 (0.031) | 0.287 (0.037) | 0.370 (0.110) | 0.261 (0.035) | 0.318 (0.066) |
|  |  | **20** | 0.661 (0.030) | 0.764 (0.031) | 0.629 (0.034) | 0.712 (0.026) | 0.286 (0.047) | 0.283 (0.127) | 0.271 (0.049) | 0.281 (0.057) |
|  |  | **10** | 0.619 (0.044) | 0.695 (0.050) | 0.567 (0.101) | 0.648 (0.038) | 0.242 (0.049) | 0.225 (0.057) | 0.229 (0.044) | 0.220 (0.044) |

# Best hyperparameters for each machine learning classification model on imbalanced data set (Table A34)

| **Hyperparameter** | **6-month Mortality** | **12-month Mortality** |
| --- | --- | --- |
| **Logistic Regression** |  |  |
| solver | liblinear | liblinear |
| penalty | l1 | l2 |
| c values | 0.1 | 0.1 |
| **RF** |  |  |
| n estimator | 2000 | 1000 |
| min sample split | 2 | 10 |
| min sample leaf | 2 | 1 |
| max features | auto | auto |
| max depth | 50 | 80 |
| bootstrap | FALSE | FALSE |
| **XGBoost** |  |  |
| n estimator | 2000 | 2000 |
| eta | 0.01 | 0.01 |
| max depth | 8 | 8 |
| min child weight | 5 | 5 |
| nthread | 4 | 4 |
| **MLP** |  |  |
| activation (Dense1) | relu | relu |
| activation (Output) | sigmoid | sigmoid |
| hidden layers | 1 | 1 |
| dropout | 0.15 | 0.15 |
| learning rate | 0.001 | 0.001 |
| neurons per layer | 512 | 512 |
| batch size | 128 | 128 |
| optimizer | adam | adam |

# 6-month mortality prediction on imbalanced data set

## Average AUCs of classifiers and frailty scale (Figure A5)

**Figure A5: Average AUCs of classifiers and frailty scale for 6-month mortality prediction on imbalanced data set**


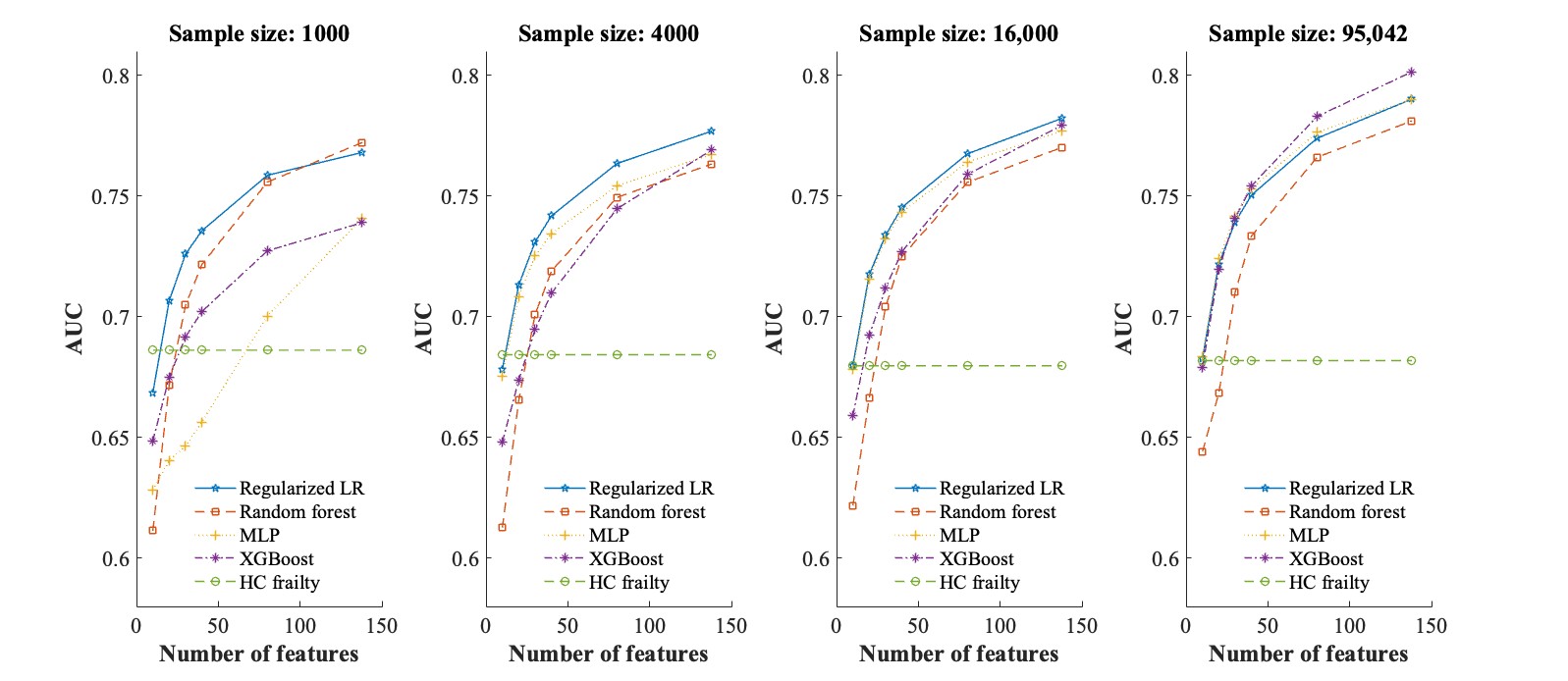
 Regularized LR=Regularized Logistic Regression. MLP=Multilayer Perceptron. XGBoost=eXtreme Gradient Boosting. HC Frailty=interRAI Home Care Frailty Scale.

## Average sensitivities of classifiers and frailty scale (Figure A6)

**Figure A6: Average sensitivities of classifiers and frailty scale for 6-month mortality prediction on imbalanced data set**


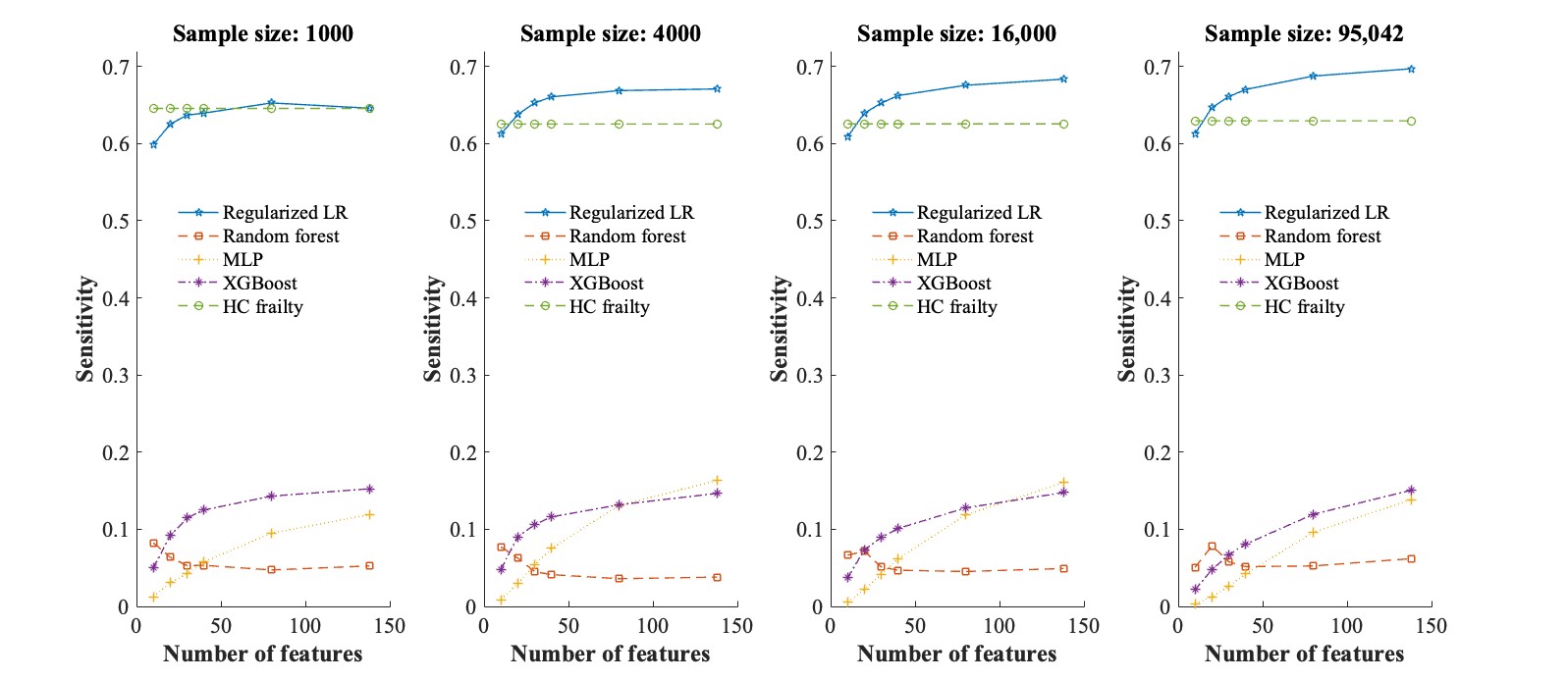
 Regularized LR=Regularized Logistic Regression. MLP=Multilayer Perceptron. XGBoost=eXtreme Gradient Boosting. HC Frailty=interRAI Home Care Frailty Scale.

## Average specificities of classifiers and frailty scale (Figure A7)

**Figure A7: Average specificities classifiers and frailty scale for 6-month mortality prediction on imbalanced data set**


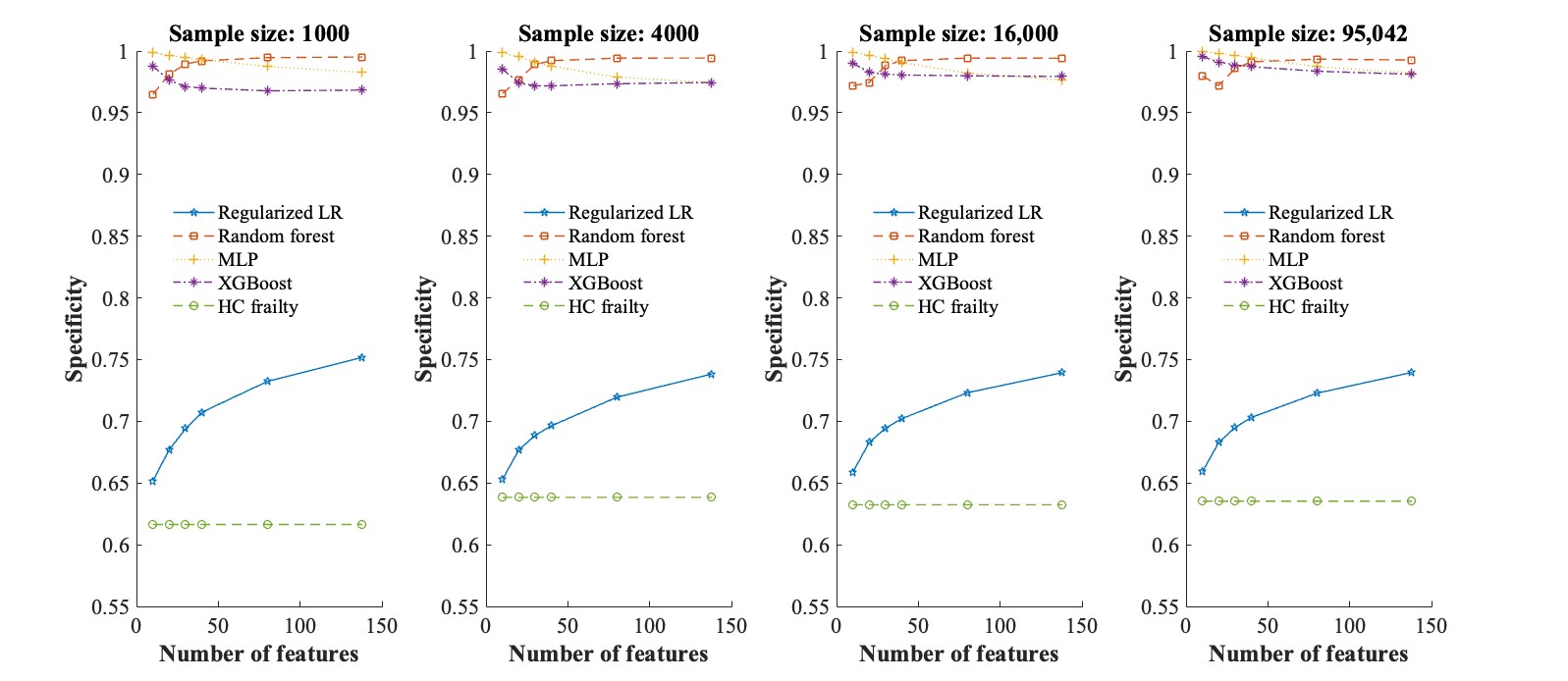
 Regularized LR=Regularized Logistic Regression. MLP=Multilayer Perceptron. XGBoost=eXtreme Gradient Boosting. HC Frailty=interRAI Home Care Frailty Scale.

## Average AUCs, sensitivities, and specificities of classifiers by test sizes (Figure A8)

**Figure A8: Average AUCs, sensitivities, and specificities of classifiers for 6-month mortality prediction by test sizes on imbalanced data set**


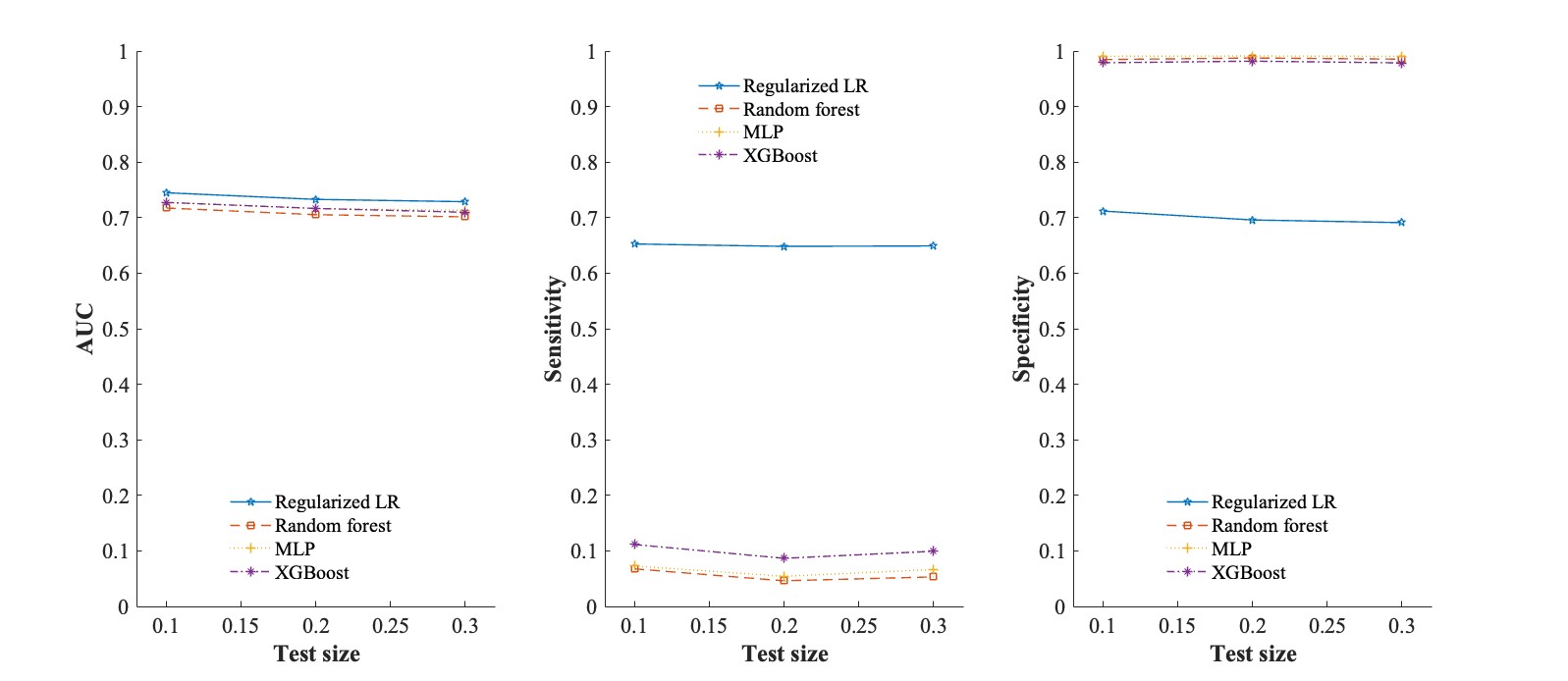
Regularized LR=Regularized Logistic Regression. RF=Random Forest. MLP=Multilayer Perceptron. XGBoost=eXtreme Gradient Boosting.

## Performance evaluation of frailty scale (Table A35 & A36)

**Table A35:**

**Average AUCs, sensitivities, and specificities of frailty scale for 6-month mortality prediction on imbalanced data set**

| **Sample Size** | **interRAI Home Care Frailty** | | |
| --- | --- | --- | --- |
|  | **Average AUC (SD)** | **Average Sensitivity (SD)** | **Average Specificity (SD)** |
| **1000** | 0.686 (0.024) | 0.646 (0.024) | 0.616 (0.027) |
| **4000** | 0.684 (0.005) | 0.625 (0.007) | 0.638 (0.004) |
| **16000** | 0.680 (0.002) | 0.626 (0.006) | 0.632 (0.004) |
| **95042** | 0.682 (0.000) | 0.629 (0.000) | 0.635 (0.000) |

SD=Standard Deviation

**Table A36:**

**Average accuracies, precisions, and F1-scores of frailty scale for 6-month mortality prediction on imbalanced data set**

| **Sample Size** | **interRAI Home Care Frailty** | | |
| --- | --- | --- | --- |
|  | **Average Accuracy (SD)** | **Average Precision (SD)** | **Average F1-score (SD)** |
| **1000** | 0.620 (0.026) | 0.186 (0.010) | 0.289 (0.011) |
| **4000** | 0.637 (0.005) | 0.211 (0.002) | 0.316 (0.002) |
| **16000** | 0.631 (0.003) | 0.207 (0.003) | 0.311 (0.002) |
| **95042** | 0.634 (0.000) | 0.206 (0.000) | 0.310 (0.000) |

SD=Standard Deviation

## Average AUCs of classifiers – test size 0.1 (Table A37)

**Table A37:**

**Average AUCs of classifiers for 6-month mortality prediction on imbalanced data set (test size 0.1)**

| **Test Size** | **Sample Size** | **Number of features** | **Average AUC (**  **SD)** | | | |
| --- | --- | --- | --- | --- | --- | --- |
|  |  |  | **RLR** | **RF** | **MLP** | **XGBoost** |
| **0.1** | **95042** | **138** | 0.790 (0.036) | 0.781 (0.034) | 0.790 (0.035) | 0.802 (0.035) |
|  |  | **80** | 0.774 (0.019) | 0.767 (0.023) | 0.777 (0.021) | 0.784 (0.020) |
|  |  | **40** | 0.750 (0.016) | 0.734 (0.021) | 0.754 (0.017) | 0.755 (0.017) |
|  |  | **30** | 0.738 (0.013) | 0.709 (0.017) | 0.741 (0.015) | 0.741 (0.014) |
|  |  | **20** | 0.722 (0.009) | 0.669 (0.011) | 0.725 (0.010) | 0.721 (0.010) |
|  |  | **10** | 0.683 (0.006) | 0.645 (0.007) | 0.684 (0.007) | 0.680 (0.006) |
|  | **16000** | **138** | 0.781 (0.039) | 0.773 (0.035) | 0.779 (0.040) | 0.785 (0.038) |
|  |  | **80** | 0.768 (0.026) | 0.759 (0.030) | 0.766 (0.027) | 0.764 (0.027) |
|  |  | **40** | 0.746 (0.021) | 0.728 (0.026) | 0.745 (0.022) | 0.732 (0.023) |
|  |  | **30** | 0.735 (0.020) | 0.708 (0.022) | 0.734 (0.020) | 0.716 (0.021) |
|  |  | **20** | 0.719 (0.017) | 0.669 (0.019) | 0.718 (0.017) | 0.697 (0.018) |
|  |  | **10** | 0.680 (0.015) | 0.623 (0.016) | 0.678 (0.015) | 0.661 (0.015) |
|  | **4000** | **138** | 0.778 (0.053) | 0.762 (0.051) | 0.768 (0.055) | 0.758 (0.053) |
|  |  | **80** | 0.767 (0.042) | 0.749 (0.046) | 0.756 (0.043) | 0.737 (0.044) |
|  |  | **40** | 0.747 (0.038) | 0.720 (0.041) | 0.738 (0.040) | 0.708 (0.042) |
|  |  | **30** | 0.737 (0.035) | 0.702 (0.038) | 0.730 (0.037) | 0.694 (0.039) |
|  |  | **20** | 0.715 (0.033) | 0.665 (0.036) | 0.709 (0.036) | 0.674 (0.035) |
|  |  | **10** | 0.681 (0.032) | 0.614 (0.034) | 0.678 (0.034) | 0.650 (0.033) |
|  | **1000** | **138** | 0.813 (0.102) | 0.813 (0.099) | 0.777 (0.139) | 0.783 (0.100) |
|  |  | **80** | 0.801 (0.085) | 0.796 (0.087) | 0.762 (0.148) | 0.772 (0.087) |
|  |  | **40** | 0.772 (0.078) | 0.758 (0.082) | 0.688 (0.145) | 0.741 (0.083) |
|  |  | **30** | 0.757 (0.075) | 0.736 (0.079) | 0.673 (0.145) | 0.724 (0.081) |
|  |  | **20** | 0.731 (0.068) | 0.699 (0.071) | 0.653 (0.089) | 0.704 (0.074) |
|  |  | **10** | 0.688 (0.063) | 0.636 (0.066) | 0.634 (0.078) | 0.673 (0.067) |

RLR=Regularized Logistic Regression. RF=Random Forest. MLP=Multilayer Perceptron. XGBoost=eXtreme Gradient Boosting. SD=Standard Deviation

## Average AUCs of classifiers – test size 0.2 (Table A38)

**Table A38:**

**Average AUCs of classifiers f or 6-month mortality prediction on imbalanced data set (test size 0.2)**

| **Test Size** | **Sample Size** | **Number of features** | **Average AUC (SD)** | | | |
| --- | --- | --- | --- | --- | --- | --- |
|  |  |  | **RLR** | **RF** | **MLP** | **XGBoost** |
| **0.2** | **95042** | **138** | 0.790 (0.033) | 0.781 (0.032) | 0.790 (0.033) | 0.802 (0.032) |
|  |  | **80** | 0.774 (0.019) | 0.766 (0.024) | 0.776 (0.021) | 0.783 (0.020) |
|  |  | **40** | 0.750 (0.014) | 0.733 (0.020) | 0.752 (0.016) | 0.754 (0.015) |
|  |  | **30** | 0.740 (0.012) | 0.711 (0.016) | 0.742 (0.014) | 0.741 (0.013) |
|  |  | **20** | 0.721 (0.008) | 0.667 (0.010) | 0.723 (0.009) | 0.719 (0.009) |
|  |  | **10** | 0.683 (0.004) | 0.645 (0.004) | 0.684 (0.004) | 0.679 (0.004) |
|  | **16000** | **138** | 0.783 (0.034) | 0.769 (0.031) | 0.775 (0.034) | 0.781 (0.033) |
|  |  | **80** | 0.766 (0.022) | 0.754 (0.028) | 0.762 (0.024) | 0.759 (0.024) |
|  |  | **40** | 0.742 (0.018) | 0.723 (0.023) | 0.741 (0.019) | 0.725 (0.020) |
|  |  | **30** | 0.731 (0.016) | 0.702 (0.019) | 0.730 (0.017) | 0.711 (0.018) |
|  |  | **20** | 0.714 (0.013) | 0.664 (0.015) | 0.712 (0.013) | 0.690 (0.015) |
|  |  | **10** | 0.678 (0.010) | 0.620 (0.011) | 0.676 (0.011) | 0.657 (0.010) |
|  | **4000** | **138** | 0.766 (0.042) | 0.757 (0.039) | 0.765 (0.043) | 0.768 (0.041) |
|  |  | **80** | 0.753 (0.032) | 0.742 (0.036) | 0.749 (0.033) | 0.740 (0.035) |
|  |  | **40** | 0.731 (0.028) | 0.711 (0.033) | 0.727 (0.030) | 0.701 (0.032) |
|  |  | **30** | 0.721 (0.027) | 0.694 (0.029) | 0.717 (0.028) | 0.689 (0.029) |
|  |  | **20** | 0.706 (0.022) | 0.659 (0.025) | 0.702 (0.025) | 0.667 (0.024) |
|  |  | **10** | 0.670 (0.021) | 0.606 (0.022) | 0.668 (0.022) | 0.640 (0.020) |
|  | **1000** | **138** | 0.755 (0.069) | 0.762 (0.075) | 0.737 (0.119) | 0.733 (0.071) |
|  |  | **80** | 0.755 (0.062) | 0.750 (0.065) | 0.685 (0.132) | 0.723 (0.065) |
|  |  | **40** | 0.737 (0.061) | 0.719 (0.061) | 0.659 (0.152) | 0.701 (0.063) |
|  |  | **30** | 0.730 (0.059) | 0.706 (0.062) | 0.640 (0.140) | 0.696 (0.063) |
|  |  | **20** | 0.713 (0.054) | 0.674 (0.051) | 0.643 (0.139) | 0.680 (0.054) |
|  |  | **10** | 0.678 (0.052) | 0.613 (0.051) | 0.636 (0.064) | 0.658 (0.052) |

RLR=Regularized Logistic Regression. RF=Random Forest. MLP=Multilayer Perceptron. XGBoost=eXtreme Gradient Boosting. SD=Standard Deviation

## Average AUCs of classifiers – test size 0.3 (Table A39)

**Table A39:**

**Average AUCs of classifiers f or 6-month mortality prediction on imbalanced data set (test size 0.3)**

| **Test Size** | **Sample Size** | **Number of features** | **Average AUC (SD)** | | | |
| --- | --- | --- | --- | --- | --- | --- |
|  |  |  | **RLR** | **RF** | **MLP** | **XGBoost** |
| **0.3** | **95042** | **138** | 0.790 (0.035) | 0.781 (0.033) | 0.789 (0.035) | 0.801 (0.034) |
|  |  | **80** | 0.774 (0.018) | 0.766 (0.023) | 0.776 (0.020) | 0.782 (0.019) |
|  |  | **40** | 0.751 (0.014) | 0.733 (0.020) | 0.753 (0.016) | 0.753 (0.016) |
|  |  | **30** | 0.739 (0.012) | 0.710 (0.016) | 0.742 (0.014) | 0.740 (0.013) |
|  |  | **20** | 0.722 (0.007) | 0.669 (0.009) | 0.724 (0.008) | 0.719 (0.008) |
|  |  | **10** | 0.682 (0.003) | 0.642 (0.003) | 0.682 (0.003) | 0.677 (0.003) |
|  | **16000** | **138** | 0.782 (0.035) | 0.768 (0.030) | 0.777 (0.035) | 0.772 (0.034) |
|  |  | **80** | 0.768 (0.021) | 0.754 (0.026) | 0.765 (0.022) | 0.754 (0.023) |
|  |  | **40** | 0.747 (0.017) | 0.724 (0.020) | 0.744 (0.017) | 0.724 (0.018) |
|  |  | **30** | 0.735 (0.014) | 0.703 (0.017) | 0.732 (0.015) | 0.709 (0.016) |
|  |  | **20** | 0.719 (0.011) | 0.666 (0.013) | 0.717 (0.011) | 0.690 (0.011) |
|  |  | **10** | 0.682 (0.008) | 0.622 (0.009) | 0.680 (0.008) | 0.659 (0.008) |
|  | **4000** | **138** | 0.786 (0.042) | 0.771 (0.039) | 0.769 (0.041) | 0.781 (0.040) |
|  |  | **80** | 0.770 (0.030) | 0.757 (0.035) | 0.758 (0.030) | 0.757 (0.033) |
|  |  | **40** | 0.747 (0.026) | 0.726 (0.028) | 0.739 (0.026) | 0.719 (0.029) |
|  |  | **30** | 0.735 (0.024) | 0.706 (0.025) | 0.728 (0.023) | 0.702 (0.025) |
|  |  | **20** | 0.718 (0.020) | 0.671 (0.020) | 0.714 (0.020) | 0.680 (0.020) |
|  |  | **10** | 0.683 (0.016) | 0.617 (0.017) | 0.680 (0.017) | 0.654 (0.016) |
|  | **1000** | **138** | 0.735 (0.063) | 0.742 (0.057) | 0.708 (0.090) | 0.701 (0.060) |
|  |  | **80** | 0.720 (0.051) | 0.721 (0.052) | 0.654 (0.101) | 0.686 (0.052) |
|  |  | **40** | 0.698 (0.046) | 0.688 (0.044) | 0.623 (0.112) | 0.664 (0.046) |
|  |  | **30** | 0.691 (0.044) | 0.672 (0.042) | 0.625 (0.119) | 0.655 (0.045) |
|  |  | **20** | 0.675 (0.040) | 0.642 (0.039) | 0.625 (0.111) | 0.641 (0.039) |
|  |  | **10** | 0.639 (0.036) | 0.584 (0.034) | 0.614 (0.041) | 0.614 (0.035) |

RLR=Regularized Logistic Regression. RF=Random Forest. MLP=Multilayer Perceptron. XGBoost=eXtreme Gradient Boosting. SD=Standard Deviation

## Average sensitivities of classifiers – test size 0.1 (Table A40)

**Table A40: Average sensitivities and specificities of classifiers for 6-month mortality prediction on imbalanced data set (test size 0.1)**

| **Test Size** | | **Sample Size** | | **Number of features** | **Average Sensitivity (SD)** | | | | **Average Specificity (SD)** | | | |  |
| --- | --- | --- | --- | --- | --- | --- | --- | --- | --- | --- | --- | --- | --- |
|  |  |  |  |  | **RLR** | **RF** | **MLP** | **XGBoost** | **RLR** | **RF** | **MLP** | **XGBoost** |  |
| **0.1** | | **95042** | | **138** | 0.697 (0.074) | 0.063 (0.039) | 0.137 (0.013) | 0.152 (0.024) | 0.739 (0.061) | 0.993 (0.022) | 0.983 (0.002) | 0.981 (0.004) |  |
|  |  |  |  | **80** | 0.688 (0.037) | 0.053 (0.026) | 0.095 (0.025) | 0.120 (0.030) | 0.723 (0.032) | 0.993 (0.006) | 0.988 (0.003) | 0.984 (0.004) |  |
|  |  |  |  | **40** | 0.670 (0.028) | 0.052 (0.029) | 0.041 (0.032) | 0.080 (0.032) | 0.703 (0.024) | 0.991 (0.005) | 0.995 (0.004) | 0.988 (0.004) |  |
|  |  |  |  | **30** | 0.660 (0.022) | 0.058 (0.028) | 0.024 (0.039) | 0.065 (0.032) | 0.694 (0.020) | 0.986 (0.004) | 0.997 (0.005) | 0.989 (0.004) |  |
|  |  |  |  | **20** | 0.648 (0.016) | 0.079 (0.022) | 0.012 (0.041) | 0.046 (0.024) | 0.683 (0.013) | 0.972 (0.003) | 0.998 (0.006) | 0.992 (0.003) |  |
|  |  |  |  | **10** | 0.612 (0.013) | 0.050 (0.007) | 0.003 (0.035) | 0.021 (0.010) | 0.661 (0.005) | 0.980 (0.001) | 1.000 (0.006) | 0.996 (0.002) |  |
|  |  | **16000** | | **138** | 0.679 (0.083) | 0.049 (0.038) | 0.152 (0.019) | 0.147 (0.030) | 0.740 (0.068) | 0.994 (0.017) | 0.978 (0.003) | 0.979 (0.006) |  |
|  |  |  |  | **80** | 0.674 (0.050) | 0.046 (0.032) | 0.111 (0.034) | 0.127 (0.032) | 0.726 (0.036) | 0.994 (0.008) | 0.983 (0.005) | 0.980 (0.005) |  |
|  |  |  |  | **40** | 0.660 (0.038) | 0.047 (0.030) | 0.058 (0.044) | 0.100 (0.033) | 0.706 (0.028) | 0.992 (0.006) | 0.991 (0.006) | 0.981 (0.005) |  |
|  |  |  |  | **30** | 0.652 (0.036) | 0.053 (0.029) | 0.041 (0.048) | 0.090 (0.032) | 0.698 (0.023) | 0.988 (0.004) | 0.994 (0.007) | 0.982 (0.005) |  |
|  |  |  |  | **20** | 0.639 (0.032) | 0.074 (0.023) | 0.021 (0.052) | 0.073 (0.029) | 0.685 (0.017) | 0.974 (0.003) | 0.997 (0.008) | 0.983 (0.004) |  |
|  |  |  |  | **10** | 0.606 (0.030) | 0.065 (0.014) | 0.006 (0.048) | 0.035 (0.023) | 0.662 (0.012) | 0.972 (0.002) | 0.999 (0.009) | 0.990 (0.004) |  |
|  |  | **4000** | | **138** | 0.669 (0.113) | 0.042 (0.046) | 0.151 (0.025) | 0.137 (0.042) | 0.737 (0.082) | 0.994 (0.015) | 0.977 (0.004) | 0.976 (0.009) |  |
|  |  |  |  | **80** | 0.670 (0.075) | 0.037 (0.042) | 0.121 (0.045) | 0.119 (0.049) | 0.724 (0.044) | 0.994 (0.011) | 0.981 (0.007) | 0.975 (0.010) |  |
|  |  |  |  | **40** | 0.664 (0.070) | 0.043 (0.035) | 0.072 (0.050) | 0.110 (0.048) | 0.702 (0.036) | 0.992 (0.007) | 0.988 (0.008) | 0.974 (0.009) |  |
|  |  |  |  | **30** | 0.653 (0.066) | 0.045 (0.033) | 0.051 (0.056) | 0.100 (0.047) | 0.698 (0.031) | 0.989 (0.005) | 0.991 (0.009) | 0.974 (0.009) |  |
|  |  |  |  | **20** | 0.633 (0.063) | 0.063 (0.028) | 0.032 (0.061) | 0.088 (0.046) | 0.686 (0.026) | 0.977 (0.005) | 0.995 (0.011) | 0.976 (0.009) |  |
|  |  |  |  | **10** | 0.608 (0.063) | 0.076 (0.027) | 0.009 (0.068) | 0.048 (0.045) | 0.662 (0.023) | 0.967 (0.004) | 0.999 (0.012) | 0.986 (0.009) |  |
|  |  | **1000** | | **138** | 0.686 (0.185) | 0.112 (0.103) | 0.194 (0.066) | 0.217 (0.103) | 0.789 (0.128) | 0.992 (0.024) | 0.979 (0.007) | 0.965 (0.018) |  |
|  |  |  |  | **80** | 0.684 (0.152) | 0.097 (0.094) | 0.168 (0.094) | 0.210 (0.112) | 0.771 (0.080) | 0.991 (0.019) | 0.984 (0.010) | 0.962 (0.021) |  |
|  |  |  |  | **40** | 0.654 (0.149) | 0.102 (0.090) | 0.101 (0.103) | 0.190 (0.115) | 0.749 (0.065) | 0.987 (0.014) | 0.991 (0.011) | 0.963 (0.020) |  |
|  |  |  |  | **30** | 0.643 (0.139) | 0.097 (0.092) | 0.073 (0.124) | 0.172 (0.120) | 0.735 (0.057) | 0.984 (0.013) | 0.993 (0.014) | 0.965 (0.021) |  |
|  |  |  |  | **20** | 0.620 (0.132) | 0.106 (0.089) | 0.053 (0.138) | 0.142 (0.126) | 0.720 (0.047) | 0.975 (0.010) | 0.995 (0.018) | 0.969 (0.022) |  |
|  |  |  | | **10** | | 0.593 (0.133) | 0.114 (0.086) | 0.023 (0.135) | 0.087 (0.112) | 0.691 (0.044) | 0.961 (0.010) | 0.998 (0.019) | 0.982 (0.020) |

## Average sensitivities of classifiers – test size 0.2 (Table A41)

**Table A41: Average sensitivities and specificities of classifiers for 6-month mortality prediction on imbalanced data set (test size 0.2)**

| **Test Size** | **Sample Size** | **Number of features** | **Average Sensitivity (SD)** | | | | **Average Specificity (SD)** | | | |
| --- | --- | --- | --- | --- | --- | --- | --- | --- | --- | --- |
|  |  |  | **RLR** | **RF** | **MLP** | **XGBoost** | **RLR** | **RF** | **MLP** | **XGBoost** |
| **0.2** | **95042** | **138** | 0.697 (0.073) | 0.062 (0.036) | 0.137 (0.016) | 0.151 (0.026) | 0.740 (0.064) | 0.993 (0.019) | 0.982 (0.002) | 0.981 (0.004) |
|  |  | **80** | 0.688 (0.036) | 0.052 (0.026) | 0.096 (0.024) | 0.119 (0.029) | 0.723 (0.033) | 0.993 (0.006) | 0.987 (0.003) | 0.984 (0.004) |
|  |  | **40** | 0.670 (0.024) | 0.051 (0.028) | 0.041 (0.034) | 0.080 (0.032) | 0.703 (0.024) | 0.991 (0.005) | 0.995 (0.004) | 0.987 (0.004) |
|  |  | **30** | 0.661 (0.020) | 0.059 (0.028) | 0.027 (0.040) | 0.067 (0.031) | 0.695 (0.020) | 0.986 (0.004) | 0.996 (0.005) | 0.989 (0.004) |
|  |  | **20** | 0.646 (0.013) | 0.078 (0.021) | 0.012 (0.043) | 0.047 (0.023) | 0.682 (0.013) | 0.972 (0.002) | 0.998 (0.006) | 0.991 (0.003) |
|  |  | **10** | 0.612 (0.009) | 0.050 (0.005) | 0.004 (0.038) | 0.022 (0.006) | 0.661 (0.004) | 0.980 (0.001) | 0.999 (0.006) | 0.995 (0.001) |
|  | **16000** | **138** | 0.686 (0.078) | 0.053 (0.035) | 0.164 (0.017) | 0.154 (0.028) | 0.740 (0.068) | 0.994 (0.017) | 0.976 (0.002) | 0.979 (0.005) |
|  |  | **80** | 0.673 (0.043) | 0.049 (0.029) | 0.124 (0.032) | 0.133 (0.031) | 0.721 (0.034) | 0.994 (0.008) | 0.981 (0.005) | 0.979 (0.005) |
|  |  | **40** | 0.662 (0.032) | 0.049 (0.027) | 0.063 (0.038) | 0.104 (0.031) | 0.698 (0.024) | 0.992 (0.005) | 0.990 (0.006) | 0.980 (0.004) |
|  |  | **30** | 0.651 (0.029) | 0.053 (0.025) | 0.043 (0.043) | 0.092 (0.030) | 0.691 (0.020) | 0.988 (0.003) | 0.994 (0.006) | 0.981 (0.004) |
|  |  | **20** | 0.637 (0.024) | 0.072 (0.018) | 0.023 (0.045) | 0.075 (0.024) | 0.680 (0.015) | 0.974 (0.002) | 0.997 (0.008) | 0.982 (0.004) |
|  |  | **10** | 0.609 (0.021) | 0.067 (0.010) | 0.006 (0.043) | 0.037 (0.016) | 0.655 (0.008) | 0.971 (0.002) | 0.999 (0.009) | 0.990 (0.003) |
|  | **4000** | **138** | 0.659 (0.096) | 0.027 (0.037) | 0.143 (0.021) | 0.127 (0.034) | 0.735 (0.082) | 0.995 (0.015) | 0.972 (0.003) | 0.973 (0.009) |
|  |  | **80** | 0.656 (0.061) | 0.027 (0.033) | 0.118 (0.036) | 0.117 (0.035) | 0.713 (0.044) | 0.995 (0.010) | 0.977 (0.006) | 0.973 (0.008) |
|  |  | **40** | 0.649 (0.053) | 0.033 (0.026) | 0.066 (0.044) | 0.104 (0.035) | 0.691 (0.035) | 0.993 (0.006) | 0.988 (0.007) | 0.971 (0.007) |
|  |  | **30** | 0.641 (0.050) | 0.040 (0.023) | 0.048 (0.047) | 0.098 (0.035) | 0.684 (0.029) | 0.989 (0.005) | 0.991 (0.008) | 0.972 (0.008) |
|  |  | **20** | 0.630 (0.046) | 0.059 (0.017) | 0.025 (0.051) | 0.084 (0.033) | 0.672 (0.023) | 0.977 (0.004) | 0.996 (0.010) | 0.974 (0.007) |
|  |  | **10** | 0.603 (0.044) | 0.074 (0.015) | 0.007 (0.054) | 0.044 (0.028) | 0.648 (0.018) | 0.965 (0.003) | 0.999 (0.013) | 0.986 (0.007) |
|  | **1000** | **138** | 0.610 (0.159) | 0.011 (0.058) | 0.055 (0.025) | 0.112 (0.046) | 0.741 (0.117) | 0.999 (0.014) | 0.987 (0.004) | 0.976 (0.008) |
|  |  | **80** | 0.647 (0.121) | 0.013 (0.044) | 0.042 (0.035) | 0.097 (0.058) | 0.727 (0.074) | 0.999 (0.010) | 0.991 (0.005) | 0.977 (0.010) |
|  |  | **40** | 0.649 (0.114) | 0.020 (0.038) | 0.025 (0.039) | 0.075 (0.062) | 0.703 (0.058) | 0.997 (0.006) | 0.996 (0.006) | 0.980 (0.011) |
|  |  | **30** | 0.651 (0.113) | 0.021 (0.036) | 0.016 (0.049) | 0.067 (0.066) | 0.687 (0.050) | 0.996 (0.005) | 0.997 (0.006) | 0.981 (0.012) |
|  |  | **20** | 0.648 (0.110) | 0.031 (0.027) | 0.012 (0.056) | 0.050 (0.070) | 0.668 (0.042) | 0.989 (0.003) | 0.998 (0.008) | 0.987 (0.012) |
|  |  | **10** | 0.622 (0.111) | 0.057 (0.024) | 0.006 (0.059) | 0.022 (0.067) | 0.642 (0.037) | 0.975 (0.002) | 0.999 (0.010) | 0.994 (0.012) |

## Average sensitivities of classifiers – test size 0.3 (Table A42)

**Table A42: Average sensitivities and specificities of classifiers for 6-month mortality prediction on imbalanced data set (test size 0.3)**

| **Test Size** | **Sample Size** | **Number of features** | **Average Sensitivity (SD)** | | | | **Average Specificity (SD)** | | | |
| --- | --- | --- | --- | --- | --- | --- | --- | --- | --- | --- |
|  |  |  | **LR** | **RF** | **MLP** | **XGBoost** | **LR** | **RF** | **MLP** | **XGBoost** |
| **0.3** | **95042** | **138** | 0.697 (0.075) | 0.061 (0.037) | 0.141 (0.013) | 0.150 (0.024) | 0.740 (0.065) | 0.993 (0.022) | 0.982 (0.002) | 0.981 (0.004) |
|  |  | **80** | 0.687 (0.037) | 0.052 (0.027) | 0.098 (0.027) | 0.120 (0.031) | 0.723 (0.033) | 0.994 (0.006) | 0.987 (0.004) | 0.983 (0.004) |
|  |  | **40** | 0.670 (0.024) | 0.051 (0.028) | 0.044 (0.035) | 0.082 (0.031) | 0.703 (0.023) | 0.991 (0.005) | 0.994 (0.005) | 0.987 (0.004) |
|  |  | **30** | 0.661 (0.019) | 0.057 (0.027) | 0.027 (0.040) | 0.069 (0.030) | 0.695 (0.019) | 0.986 (0.004) | 0.996 (0.005) | 0.988 (0.004) |
|  |  | **20** | 0.646 (0.012) | 0.079 (0.021) | 0.014 (0.042) | 0.050 (0.022) | 0.684 (0.012) | 0.972 (0.002) | 0.998 (0.006) | 0.991 (0.002) |
|  |  | **10** | 0.615 (0.007) | 0.051 (0.004) | 0.003 (0.035) | 0.022 (0.005) | 0.656 (0.003) | 0.979 (0.001) | 1.000 (0.006) | 0.995 (0.001) |
|  | **16000** | **138** | 0.685 (0.075) | 0.045 (0.034) | 0.166 (0.019) | 0.142 (0.028) | 0.738 (0.065) | 0.994 (0.016) | 0.976 (0.003) | 0.980 (0.005) |
|  |  | **80** | 0.680 (0.042) | 0.041 (0.025) | 0.121 (0.030) | 0.123 (0.027) | 0.722 (0.035) | 0.995 (0.007) | 0.982 (0.004) | 0.980 (0.004) |
|  |  | **40** | 0.665 (0.031) | 0.045 (0.024) | 0.064 (0.038) | 0.100 (0.026) | 0.703 (0.027) | 0.993 (0.004) | 0.990 (0.005) | 0.980 (0.004) |
|  |  | **30** | 0.655 (0.027) | 0.047 (0.023) | 0.041 (0.042) | 0.087 (0.026) | 0.695 (0.022) | 0.989 (0.003) | 0.994 (0.006) | 0.981 (0.004) |
|  |  | **20** | 0.642 (0.021) | 0.068 (0.017) | 0.021 (0.047) | 0.072 (0.021) | 0.684 (0.015) | 0.976 (0.002) | 0.997 (0.008) | 0.983 (0.003) |
|  |  | **10** | 0.611 (0.017) | 0.068 (0.009) | 0.006 (0.047) | 0.039 (0.014) | 0.660 (0.007) | 0.972 (0.002) | 0.999 (0.009) | 0.990 (0.003) |
|  | **4000** | **138** | 0.685 (0.094) | 0.045 (0.038) | 0.196 (0.034) | 0.176 (0.039) | 0.743 (0.075) | 0.994 (0.014) | 0.974 (0.004) | 0.975 (0.009) |
|  |  | **80** | 0.680 (0.061) | 0.044 (0.039) | 0.155 (0.050) | 0.159 (0.042) | 0.721 (0.043) | 0.994 (0.009) | 0.978 (0.006) | 0.973 (0.007) |
|  |  | **40** | 0.669 (0.049) | 0.047 (0.038) | 0.088 (0.059) | 0.134 (0.042) | 0.695 (0.034) | 0.992 (0.006) | 0.987 (0.007) | 0.970 (0.007) |
|  |  | **30** | 0.663 (0.045) | 0.050 (0.037) | 0.062 (0.061) | 0.120 (0.041) | 0.684 (0.031) | 0.989 (0.005) | 0.991 (0.008) | 0.970 (0.007) |
|  |  | **20** | 0.650 (0.039) | 0.066 (0.027) | 0.033 (0.060) | 0.098 (0.036) | 0.672 (0.024) | 0.976 (0.004) | 0.995 (0.010) | 0.972 (0.006) |
|  |  | **10** | 0.625 (0.036) | 0.082 (0.017) | 0.011 (0.059) | 0.052 (0.028) | 0.648 (0.014) | 0.964 (0.003) | 0.999 (0.012) | 0.985 (0.006) |
|  | **1000** | **138** | 0.641 (0.176) | 0.035 (0.051) | 0.108 (0.026) | 0.129 (0.045) | 0.725 (0.148) | 0.995 (0.018) | 0.982 (0.005) | 0.964 (0.012) |
|  |  | **80** | 0.627 (0.117) | 0.032 (0.042) | 0.075 (0.041) | 0.122 (0.053) | 0.699 (0.093) | 0.994 (0.013) | 0.988 (0.006) | 0.965 (0.013) |
|  |  | **40** | 0.616 (0.098) | 0.038 (0.037) | 0.047 (0.045) | 0.110 (0.052) | 0.670 (0.067) | 0.992 (0.008) | 0.992 (0.008) | 0.967 (0.014) |
|  |  | **30** | 0.616 (0.089) | 0.041 (0.035) | 0.039 (0.049) | 0.104 (0.052) | 0.660 (0.056) | 0.989 (0.007) | 0.994 (0.008) | 0.967 (0.014) |
|  |  | **20** | 0.608 (0.078) | 0.054 (0.028) | 0.027 (0.056) | 0.083 (0.054) | 0.642 (0.042) | 0.979 (0.005) | 0.996 (0.010) | 0.973 (0.013) |
|  |  | **10** | 0.581 (0.074) | 0.078 (0.027) | 0.009 (0.054) | 0.040 (0.049) | 0.622 (0.033) | 0.958 (0.005) | 0.998 (0.011) | 0.987 (0.013) |

## Average F1-scores of classifiers – test size 0.1 (Table A43)

**Table A43:**

**Average F1-scores of classifiers for 6-month mortality prediction on imbalanced data set (test size 0.1)**

| **Test Size** | **Sample Size** | **Number of features** | **Average F1-score (SD)** | | | |
| --- | --- | --- | --- | --- | --- | --- |
|  |  |  | **RLR** | **RF** | **MLP** | **XGBosst** |
| **0.1** | **95042** | **138** | 0.406 (0.009) | 0.114 (0.011) | 0.216 (0.044) | 0.237 (0.013) |
|  |  | **80** | 0.389 (0.011) | 0.096 (0.037) | 0.157 (0.060) | 0.194 (0.033) |
|  |  | **40** | 0.368 (0.014) | 0.092 (0.046) | 0.072 (0.063) | 0.136 (0.048) |
|  |  | **30** | 0.358 (0.015) | 0.098 (0.044) | 0.042 (0.053) | 0.111 (0.049) |
|  |  | **20** | 0.345 (0.017) | 0.123 (0.036) | 0.021 (0.043) | 0.081 (0.047) |
|  |  | **10** | 0.317 (0.026) | 0.079 (0.049) | 0.006 (0.022) | 0.038 (0.040) |
|  | **16000** | **138** | 0.404 (0.021) | 0.089 (0.025) | 0.230 (0.058) | 0.229 (0.032) |
|  |  | **80** | 0.390 (0.023) | 0.084 (0.039) | 0.176 (0.071) | 0.200 (0.041) |
|  |  | **40** | 0.370 (0.023) | 0.083 (0.048) | 0.099 (0.074) | 0.162 (0.046) |
|  |  | **30** | 0.361 (0.024) | 0.092 (0.047) | 0.070 (0.070) | 0.146 (0.048) |
|  |  | **20** | 0.347 (0.026) | 0.117 (0.045) | 0.037 (0.056) | 0.121 (0.048) |
|  |  | **10** | 0.319 (0.033) | 0.101 (0.050) | 0.010 (0.032) | 0.061 (0.048) |
|  | **4000** | **138** | 0.387 (0.043) | 0.076 (0.046) | 0.224 (0.084) | 0.208 (0.062) |
|  |  | **80** | 0.379 (0.043) | 0.068 (0.049) | 0.187 (0.083) | 0.183 (0.063) |
|  |  | **40** | 0.361 (0.042) | 0.077 (0.055) | 0.118 (0.084) | 0.169 (0.065) |
|  |  | **30** | 0.355 (0.044) | 0.078 (0.057) | 0.087 (0.078) | 0.154 (0.067) |
|  |  | **20** | 0.337 (0.045) | 0.101 (0.062) | 0.054 (0.072) | 0.137 (0.069) |
|  |  | **10** | 0.313 (0.049) | 0.114 (0.064) | 0.015 (0.042) | 0.080 (0.065) |
|  | **1000** | **138** | 0.433 (0.093) | 0.181 (0.127) | 0.268 (0.156) | 0.286 (0.129) |
|  |  | **80** | 0.415 (0.096) | 0.156 (0.132) | 0.236 (0.168) | 0.272 (0.145) |
|  |  | **40** | 0.383 (0.095) | 0.159 (0.132) | 0.149 (0.167) | 0.251 (0.142) |
|  |  | **30** | 0.368 (0.095) | 0.149 (0.129) | 0.112 (0.147) | 0.231 (0.138) |
|  |  | **20** | 0.348 (0.097) | 0.155 (0.127) | 0.081 (0.135) | 0.196 (0.139) |
|  |  | **10** | 0.317 (0.104) | 0.153 (0.126) | 0.035 (0.096) | 0.128 (0.140) |

RLR=Regularized Logistic Regression. RF=Random Forest. MLP=Multilayer Perceptron. XGBoost=eXtreme Gradient Boosting. SD=Standard Deviation

## Average F1-scores of classifiers – test size 0.2 (Table A44)

**Table A44:**

**Average F1- scores of classifiers for 6-month mortality prediction on imbalanced data set (test size 0.2)**

| **Test Size** | **Sample Size** | **Number of features** | **Average F1-score (SD)** | | | |
| --- | --- | --- | --- | --- | --- | --- |
|  |  |  | **RLR** | **RF** | **MLP** | **XGBosst** |
| **0.2** | **95042** | **138** | 0.407 (0.006) | 0.111 (0.008) | 0.216 (0.047) | 0.236 (0.008) |
|  |  | **80** | 0.389 (0.009) | 0.094 (0.036) | 0.158 (0.063) | 0.193 (0.033) |
|  |  | **40** | 0.368 (0.012) | 0.091 (0.045) | 0.072 (0.065) | 0.135 (0.047) |
|  |  | **30** | 0.359 (0.013) | 0.100 (0.043) | 0.048 (0.057) | 0.116 (0.048) |
|  |  | **20** | 0.344 (0.016) | 0.122 (0.035) | 0.021 (0.040) | 0.083 (0.047) |
|  |  | **10** | 0.317 (0.025) | 0.080 (0.047) | 0.007 (0.027) | 0.040 (0.042) |
|  | **16000** | **138** | 0.408 (0.014) | 0.098 (0.018) | 0.245 (0.050) | 0.238 (0.021) |
|  |  | **80** | 0.388 (0.017) | 0.089 (0.031) | 0.195 (0.060) | 0.210 (0.033) |
|  |  | **40** | 0.367 (0.018) | 0.087 (0.042) | 0.108 (0.066) | 0.167 (0.043) |
|  |  | **30** | 0.358 (0.018) | 0.092 (0.043) | 0.075 (0.062) | 0.149 (0.045) |
|  |  | **20** | 0.346 (0.020) | 0.115 (0.041) | 0.041 (0.053) | 0.124 (0.045) |
|  |  | **10** | 0.319 (0.027) | 0.103 (0.046) | 0.011 (0.030) | 0.065 (0.046) |
|  | **4000** | **138** | 0.396 (0.029) | 0.050 (0.026) | 0.211 (0.065) | 0.194 (0.038) |
|  |  | **80** | 0.379 (0.029) | 0.050 (0.030) | 0.182 (0.068) | 0.180 (0.045) |
|  |  | **40** | 0.362 (0.030) | 0.059 (0.040) | 0.110 (0.070) | 0.160 (0.048) |
|  |  | **30** | 0.353 (0.030) | 0.071 (0.042) | 0.083 (0.069) | 0.152 (0.049) |
|  |  | **20** | 0.343 (0.032) | 0.096 (0.048) | 0.044 (0.058) | 0.133 (0.050) |
|  |  | **10** | 0.318 (0.037) | 0.112 (0.050) | 0.012 (0.035) | 0.075 (0.053) |
|  | **1000** | **138** | 0.298 (0.061) | 0.021 (0.044) | 0.086 (0.087) | 0.160 (0.086) |
|  |  | **80** | 0.306 (0.061) | 0.024 (0.048) | 0.068 (0.087) | 0.140 (0.095) |
|  |  | **40** | 0.293 (0.059) | 0.035 (0.061) | 0.042 (0.077) | 0.113 (0.093) |
|  |  | **30** | 0.284 (0.060) | 0.036 (0.062) | 0.027 (0.063) | 0.102 (0.088) |
|  |  | **20** | 0.271 (0.060) | 0.051 (0.070) | 0.020 (0.056) | 0.079 (0.087) |
|  |  | **10** | 0.253 (0.060) | 0.083 (0.080) | 0.009 (0.039) | 0.037 (0.072) |

RLR=Regularized Logistic Regression. RF=Random Forest. MLP=Multilayer Perceptron. XGBoost=eXtreme Gradient Boosting. SD=Standard Deviation

## Average F1-scores of classifiers – test size 0.3 (Table A45)

**Table A45:**

**Average F1- scores of classifiers for 6-month mortality prediction on imbalanced data set (test size 0.3)**

| **Test Size** | **Sample Size** | **Number of features** | **Average F1-score (SD)** | | | |
| --- | --- | --- | --- | --- | --- | --- |
|  |  |  | **RLR** | **RF** | **MLP** | **XGBosst** |
| **0.3** | **95042** | **138** | 0.406 (0.005) | 0.110 (0.007) | 0.220 (0.043) | 0.235 (0.007) |
|  |  | **80** | 0.389 (0.008) | 0.094 (0.035) | 0.162 (0.060) | 0.194 (0.031) |
|  |  | **40** | 0.368 (0.012) | 0.090 (0.044) | 0.077 (0.065) | 0.138 (0.045) |
|  |  | **30** | 0.359 (0.013) | 0.098 (0.043) | 0.049 (0.058) | 0.117 (0.047) |
|  |  | **20** | 0.345 (0.015) | 0.124 (0.037) | 0.024 (0.046) | 0.088 (0.049) |
|  |  | **10** | 0.316 (0.026) | 0.081 (0.047) | 0.006 (0.022) | 0.040 (0.040) |
|  | **16000** | **138** | 0.399 (0.011) | 0.083 (0.015) | 0.247 (0.054) | 0.222 (0.018) |
|  |  | **80** | 0.384 (0.014) | 0.075 (0.029) | 0.191 (0.061) | 0.195 (0.030) |
|  |  | **40** | 0.364 (0.015) | 0.081 (0.038) | 0.109 (0.064) | 0.161 (0.037) |
|  |  | **30** | 0.355 (0.017) | 0.082 (0.038) | 0.072 (0.060) | 0.142 (0.038) |
|  |  | **20** | 0.342 (0.019) | 0.108 (0.035) | 0.037 (0.050) | 0.120 (0.040) |
|  |  | **10** | 0.315 (0.026) | 0.104 (0.044) | 0.011 (0.032) | 0.068 (0.045) |
|  | **4000** | **138** | 0.412 (0.022) | 0.083 (0.030) | 0.282 (0.064) | 0.262 (0.034) |
|  |  | **80** | 0.393 (0.025) | 0.079 (0.046) | 0.233 (0.077) | 0.237 (0.045) |
|  |  | **40** | 0.370 (0.027) | 0.083 (0.060) | 0.142 (0.088) | 0.200 (0.053) |
|  |  | **30** | 0.360 (0.027) | 0.085 (0.060) | 0.102 (0.088) | 0.180 (0.055) |
|  |  | **20** | 0.348 (0.030) | 0.105 (0.056) | 0.055 (0.077) | 0.151 (0.057) |
|  |  | **10** | 0.323 (0.034) | 0.121 (0.049) | 0.018 (0.054) | 0.087 (0.060) |
|  | **1000** | **138** | 0.379 (0.045) | 0.063 (0.047) | 0.171 (0.075) | 0.187 (0.062) |
|  |  | **80** | 0.357 (0.046) | 0.057 (0.049) | 0.124 (0.087) | 0.178 (0.068) |
|  |  | **40** | 0.334 (0.047) | 0.067 (0.058) | 0.079 (0.079) | 0.163 (0.069) |
|  |  | **30** | 0.327 (0.048) | 0.071 (0.060) | 0.067 (0.073) | 0.155 (0.070) |
|  |  | **20** | 0.314 (0.049) | 0.088 (0.064) | 0.048 (0.069) | 0.128 (0.074) |
|  |  | **10** | 0.290 (0.060) | 0.112 (0.067) | 0.015 (0.044) | 0.066 (0.070) |

RLR=Regularized Logistic Regression. RF=Random Forest. MLP=Multilayer Perceptron. XGBoost=eXtreme Gradient Boosting. SD=Standard Deviation

## Average accuracies and precisions of classifiers – test size 0.1 (Table A46)

**Table A46: Average accuracies and precisions of classifiers for 6-month mortality prediction on imbalanced data set (test size 0.1)**

| **Test Size** | **Sample Size** | **Number of features** | **Average Accuracy (SD)** | | | | **Average Precision (SD)** | | | |
| --- | --- | --- | --- | --- | --- | --- | --- | --- | --- | --- |
|  |  |  | **LR** | **RF** | **MLP** | **XGBoost** | **LR** | **RF** | **MLP** | **XGBoost** |
| **0.1** | **95042** | **138** | 0.734 (0.004) | 0.871 (0.003) | 0.872 (0.003) | 0.873 (0.003) | 0.286 (0.008) | 0.565 (0.041) | 0.546 (0.033) | 0.547 (0.025) |
|  |  | **80** | 0.718 (0.011) | 0.871 (0.003) | 0.871 (0.003) | 0.871 (0.003) | 0.271 (0.010) | 0.542 (0.052) | 0.537 (0.042) | 0.522 (0.033) |
|  |  | **40** | 0.699 (0.016) | 0.869 (0.003) | 0.870 (0.003) | 0.869 (0.003) | 0.253 (0.013) | 0.456 (0.067) | 0.529 (0.120) | 0.485 (0.044) |
|  |  | **30** | 0.690 (0.019) | 0.865 (0.004) | 0.870 (0.003) | 0.869 (0.003) | 0.245 (0.014) | 0.371 (0.071) | 0.479 (0.226) | 0.464 (0.052) |
|  |  | **20** | 0.678 (0.025) | 0.855 (0.005) | 0.870 (0.003) | 0.868 (0.003) | 0.236 (0.015) | 0.293 (0.052) | 0.355 (0.329) | 0.432 (0.063) |
|  |  | **10** | 0.654 (0.047) | 0.858 (0.015) | 0.869 (0.003) | 0.868 (0.003) | 0.215 (0.022) | 0.285 (0.071) | 0.116 (0.255) | 0.372 (0.106) |
|  | **16000** | **138** | 0.732 (0.011) | 0.867 (0.008) | 0.867 (0.008) | 0.868 (0.008) | 0.288 (0.018) | 0.549 (0.113) | 0.520 (0.070) | 0.523 (0.062) |
|  |  | **80** | 0.719 (0.014) | 0.868 (0.008) | 0.867 (0.008) | 0.866 (0.008) | 0.274 (0.020) | 0.540 (0.131) | 0.501 (0.091) | 0.493 (0.067) |
|  |  | **40** | 0.700 (0.019) | 0.866 (0.008) | 0.867 (0.008) | 0.863 (0.008) | 0.257 (0.020) | 0.461 (0.126) | 0.497 (0.169) | 0.442 (0.078) |
|  |  | **30** | 0.692 (0.023) | 0.863 (0.008) | 0.867 (0.008) | 0.863 (0.008) | 0.250 (0.021) | 0.399 (0.118) | 0.485 (0.235) | 0.421 (0.083) |
|  |  | **20** | 0.679 (0.029) | 0.854 (0.010) | 0.867 (0.008) | 0.861 (0.008) | 0.239 (0.022) | 0.303 (0.082) | 0.382 (0.325) | 0.391 (0.086) |
|  |  | **10** | 0.654 (0.052) | 0.851 (0.014) | 0.867 (0.008) | 0.863 (0.008) | 0.219 (0.028) | 0.266 (0.083) | 0.119 (0.264) | 0.333 (0.146) |
|  | **4000** | **138** | 0.728 (0.021) | 0.871 (0.016) | 0.870 (0.016) | 0.868 (0.016) | 0.274 (0.037) | 0.510 (0.282) | 0.503 (0.138) | 0.461 (0.127) |
|  |  | **80** | 0.717 (0.024) | 0.870 (0.016) | 0.870 (0.016) | 0.865 (0.016) | 0.265 (0.037) | 0.461 (0.298) | 0.486 (0.170) | 0.417 (0.130) |
|  |  | **40** | 0.697 (0.027) | 0.869 (0.015) | 0.869 (0.016) | 0.862 (0.016) | 0.250 (0.035) | 0.442 (0.265) | 0.450 (0.248) | 0.388 (0.132) |
|  |  | **30** | 0.692 (0.031) | 0.866 (0.016) | 0.869 (0.016) | 0.860 (0.016) | 0.245 (0.037) | 0.372 (0.245) | 0.410 (0.295) | 0.363 (0.137) |
|  |  | **20** | 0.679 (0.037) | 0.859 (0.016) | 0.871 (0.015) | 0.861 (0.016) | 0.231 (0.037) | 0.289 (0.164) | 0.297 (0.326) | 0.340 (0.146) |
|  |  | **10** | 0.655 (0.064) | 0.852 (0.018) | 0.871 (0.015) | 0.865 (0.016) | 0.214 (0.041) | 0.257 (0.133) | 0.107 (0.254) | 0.318 (0.229) |
|  | **1000** | **138** | 0.776 (0.040) | 0.879 (0.032) | 0.878 (0.032) | 0.869 (0.031) | 0.322 (0.085) | 0.608 (0.381) | 0.568 (0.297) | 0.483 (0.224) |
|  |  | **80** | 0.760 (0.044) | 0.877 (0.031) | 0.879 (0.033) | 0.866 (0.034) | 0.304 (0.085) | 0.516 (0.400) | 0.548 (0.345) | 0.441 (0.235) |
|  |  | **40** | 0.737 (0.052) | 0.874 (0.032) | 0.877 (0.034) | 0.865 (0.034) | 0.277 (0.084) | 0.468 (0.369) | 0.384 (0.397) | 0.424 (0.238) |
|  |  | **30** | 0.723 (0.056) | 0.871 (0.032) | 0.875 (0.033) | 0.863 (0.033) | 0.265 (0.081) | 0.419 (0.356) | 0.328 (0.401) | 0.408 (0.239) |
|  |  | **20** | 0.708 (0.068) | 0.864 (0.034) | 0.875 (0.033) | 0.863 (0.034) | 0.250 (0.084) | 0.372 (0.311) | 0.245 (0.381) | 0.388 (0.275) |
|  |  | **10** | 0.679 (0.104) | 0.855 (0.033) | 0.876 (0.032) | 0.870 (0.033) | 0.229 (0.099) | 0.288 (0.247) | 0.112 (0.287) | 0.330 (0.352) |

## Average accuracies and precisions of classifiers – test size 0.2 (Table A47)

**Table A47: Average accuracies and precisions of classifiers for 6-month mortality prediction on imbalanced data set (test size 0.2)**

| **Test Size** | **Sample Size** | **Number of features** | **Average Accuracy (SD)** | | | | **Average Precision (SD)** | | | |
| --- | --- | --- | --- | --- | --- | --- | --- | --- | --- | --- |
|  |  |  | **LR** | **RF** | **MLP** | **XGBoost** | **LR** | **RF** | **MLP** | **XGBoost** |
| **0.2** | **95042** | **138** | 0.734 (0.003) | 0.871 (0.002) | 0.872 (0.002) | 0.873 (0.002) | 0.287 (0.005) | 0.563 (0.028) | 0.544 (0.027) | 0.546 (0.018) |
|  |  | **80** | 0.718 (0.010) | 0.871 (0.002) | 0.871 (0.002) | 0.871 (0.002) | 0.271 (0.009) | 0.540 (0.038) | 0.536 (0.036) | 0.521 (0.026) |
|  |  | **40** | 0.699 (0.016) | 0.869 (0.003) | 0.870 (0.002) | 0.869 (0.002) | 0.254 (0.012) | 0.455 (0.059) | 0.523 (0.102) | 0.480 (0.037) |
|  |  | **30** | 0.691 (0.019) | 0.865 (0.004) | 0.870 (0.002) | 0.869 (0.002) | 0.246 (0.012) | 0.377 (0.068) | 0.500 (0.185) | 0.463 (0.042) |
|  |  | **20** | 0.678 (0.026) | 0.855 (0.005) | 0.870 (0.002) | 0.868 (0.002) | 0.235 (0.015) | 0.291 (0.050) | 0.380 (0.297) | 0.426 (0.051) |
|  |  | **10** | 0.654 (0.049) | 0.859 (0.013) | 0.870 (0.002) | 0.868 (0.002) | 0.215 (0.022) | 0.285 (0.066) | 0.144 (0.272) | 0.375 (0.085) |
|  | **16000** | **138** | 0.733 (0.007) | 0.868 (0.005) | 0.867 (0.006) | 0.868 (0.005) | 0.291 (0.012) | 0.593 (0.075) | 0.520 (0.049) | 0.531 (0.044) |
|  |  | **80** | 0.715 (0.012) | 0.867 (0.005) | 0.866 (0.006) | 0.865 (0.006) | 0.273 (0.014) | 0.560 (0.082) | 0.513 (0.058) | 0.500 (0.048) |
|  |  | **40** | 0.693 (0.017) | 0.865 (0.006) | 0.866 (0.006) | 0.863 (0.006) | 0.254 (0.016) | 0.476 (0.095) | 0.503 (0.114) | 0.444 (0.060) |
|  |  | **30** | 0.686 (0.019) | 0.862 (0.006) | 0.866 (0.006) | 0.861 (0.006) | 0.247 (0.016) | 0.399 (0.097) | 0.491 (0.169) | 0.416 (0.064) |
|  |  | **20** | 0.675 (0.026) | 0.853 (0.007) | 0.865 (0.006) | 0.860 (0.006) | 0.238 (0.017) | 0.299 (0.068) | 0.405 (0.271) | 0.388 (0.070) |
|  |  | **10** | 0.649 (0.052) | 0.850 (0.012) | 0.865 (0.005) | 0.862 (0.006) | 0.218 (0.024) | 0.268 (0.071) | 0.159 (0.279) | 0.349 (0.108) |
|  | **4000** | **138** | 0.724 (0.015) | 0.862 (0.011) | 0.858 (0.011) | 0.857 (0.011) | 0.283 (0.025) | 0.490 (0.219) | 0.462 (0.095) | 0.431 (0.081) |
|  |  | **80** | 0.705 (0.019) | 0.862 (0.010) | 0.859 (0.011) | 0.855 (0.011) | 0.267 (0.025) | 0.456 (0.235) | 0.460 (0.115) | 0.404 (0.081) |
|  |  | **40** | 0.685 (0.024) | 0.861 (0.010) | 0.861 (0.011) | 0.852 (0.011) | 0.251 (0.025) | 0.410 (0.206) | 0.462 (0.183) | 0.364 (0.088) |
|  |  | **30** | 0.678 (0.028) | 0.859 (0.011) | 0.862 (0.011) | 0.852 (0.011) | 0.245 (0.026) | 0.378 (0.166) | 0.448 (0.233) | 0.352 (0.089) |
|  |  | **20** | 0.666 (0.035) | 0.850 (0.012) | 0.862 (0.011) | 0.851 (0.011) | 0.237 (0.027) | 0.288 (0.115) | 0.381 (0.341) | 0.340 (0.096) |
|  |  | **10** | 0.642 (0.061) | 0.842 (0.015) | 0.862 (0.011) | 0.856 (0.012) | 0.218 (0.031) | 0.255 (0.092) | 0.130 (0.286) | 0.318 (0.172) |
|  | **1000** | **138** | 0.729 (0.032) | 0.905 (0.019) | 0.898 (0.019) | 0.893 (0.019) | 0.200 (0.048) | 0.182 (0.378) | 0.272 (0.284) | 0.333 (0.182) |
|  |  | **80** | 0.720 (0.036) | 0.904 (0.018) | 0.900 (0.018) | 0.892 (0.019) | 0.202 (0.047) | 0.183 (0.365) | 0.237 (0.307) | 0.305 (0.210) |
|  |  | **40** | 0.698 (0.044) | 0.902 (0.018) | 0.901 (0.018) | 0.892 (0.019) | 0.192 (0.045) | 0.209 (0.363) | 0.163 (0.293) | 0.281 (0.239) |
|  |  | **30** | 0.683 (0.050) | 0.901 (0.018) | 0.902 (0.018) | 0.893 (0.019) | 0.184 (0.046) | 0.195 (0.341) | 0.123 (0.279) | 0.275 (0.252) |
|  |  | **20** | 0.666 (0.063) | 0.897 (0.020) | 0.904 (0.020) | 0.897 (0.020) | 0.175 (0.049) | 0.198 (0.286) | 0.088 (0.241) | 0.250 (0.285) |
|  |  | **10** | 0.640 (0.096) | 0.885 (0.021) | 0.902 (0.019) | 0.899 (0.019) | 0.164 (0.050) | 0.191 (0.197) | 0.034 (0.150) | 0.149 (0.290) |

## Average accuracies and precisions of classifiers – test size 0.3 (Table A48)

**Table A48: Average accuracies and precisions of classifiers for 6-month mortality prediction on imbalanced data set (test size 0.3)**

| **Test Size** | **Sample Size** | **Number of features** | **Average Accuracy (SD)** | | | | **Average Precision (SD)** | | | |
| --- | --- | --- | --- | --- | --- | --- | --- | --- | --- | --- |
|  |  |  | **LR** | **RF** | **MLP** | **XGBoost** | **LR** | **RF** | **MLP** | **XGBoost** |
| **0.3** | **95042** | **138** | 0.734 (0.003) | 0.871 (0.002) | 0.872 (0.002) | 0.873 (0.002) | 0.287 (0.004) | 0.562 (0.023) | 0.540 (0.024) | 0.543 (0.015) |
|  |  | **80** | 0.718 (0.010) | 0.871 (0.002) | 0.871 (0.002) | 0.871 (0.002) | 0.272 (0.008) | 0.542 (0.031) | 0.535 (0.029) | 0.518 (0.022) |
|  |  | **40** | 0.699 (0.016) | 0.869 (0.002) | 0.870 (0.002) | 0.869 (0.002) | 0.254 (0.011) | 0.455 (0.057) | 0.525 (0.071) | 0.478 (0.034) |
|  |  | **30** | 0.691 (0.019) | 0.865 (0.003) | 0.870 (0.002) | 0.868 (0.002) | 0.246 (0.012) | 0.374 (0.067) | 0.508 (0.144) | 0.457 (0.039) |
|  |  | **20** | 0.679 (0.026) | 0.856 (0.004) | 0.870 (0.002) | 0.868 (0.002) | 0.236 (0.014) | 0.296 (0.050) | 0.418 (0.276) | 0.426 (0.048) |
|  |  | **10** | 0.651 (0.050) | 0.858 (0.015) | 0.869 (0.002) | 0.868 (0.002) | 0.214 (0.022) | 0.282 (0.063) | 0.159 (0.275) | 0.370 (0.072) |
|  | **16000** | **138** | 0.731 (0.006) | 0.871 (0.004) | 0.871 (0.004) | 0.871 (0.004) | 0.281 (0.010) | 0.556 (0.069) | 0.519 (0.041) | 0.515 (0.036) |
|  |  | **80** | 0.717 (0.012) | 0.871 (0.004) | 0.870 (0.004) | 0.869 (0.004) | 0.268 (0.013) | 0.538 (0.079) | 0.511 (0.051) | 0.482 (0.043) |
|  |  | **40** | 0.698 (0.018) | 0.869 (0.004) | 0.870 (0.004) | 0.866 (0.004) | 0.251 (0.014) | 0.468 (0.078) | 0.505 (0.083) | 0.429 (0.050) |
|  |  | **30** | 0.690 (0.021) | 0.867 (0.005) | 0.870 (0.004) | 0.865 (0.004) | 0.244 (0.016) | 0.388 (0.088) | 0.495 (0.134) | 0.403 (0.054) |
|  |  | **20** | 0.678 (0.027) | 0.858 (0.006) | 0.870 (0.004) | 0.864 (0.004) | 0.234 (0.016) | 0.293 (0.061) | 0.446 (0.241) | 0.377 (0.058) |
|  |  | **10** | 0.653 (0.050) | 0.854 (0.012) | 0.870 (0.004) | 0.866 (0.004) | 0.214 (0.023) | 0.264 (0.062) | 0.181 (0.288) | 0.348 (0.099) |
|  | **4000** | **138** | 0.735 (0.012) | 0.865 (0.008) | 0.868 (0.008) | 0.866 (0.008) | 0.295 (0.019) | 0.550 (0.128) | 0.553 (0.073) | 0.523 (0.060) |
|  |  | **80** | 0.716 (0.019) | 0.865 (0.008) | 0.867 (0.009) | 0.863 (0.009) | 0.277 (0.022) | 0.521 (0.173) | 0.528 (0.095) | 0.476 (0.070) |
|  |  | **40** | 0.692 (0.025) | 0.864 (0.009) | 0.865 (0.009) | 0.857 (0.009) | 0.256 (0.023) | 0.441 (0.161) | 0.497 (0.141) | 0.409 (0.081) |
|  |  | **30** | 0.681 (0.027) | 0.862 (0.009) | 0.865 (0.009) | 0.855 (0.009) | 0.248 (0.023) | 0.385 (0.142) | 0.487 (0.190) | 0.377 (0.081) |
|  |  | **20** | 0.669 (0.034) | 0.853 (0.010) | 0.865 (0.009) | 0.853 (0.009) | 0.239 (0.025) | 0.297 (0.109) | 0.418 (0.310) | 0.348 (0.089) |
|  |  | **10** | 0.645 (0.056) | 0.844 (0.012) | 0.865 (0.008) | 0.858 (0.009) | 0.220 (0.029) | 0.259 (0.081) | 0.163 (0.303) | 0.331 (0.143) |
|  | **1000** | **138** | 0.713 (0.028) | 0.863 (0.016) | 0.861 (0.016) | 0.849 (0.017) | 0.271 (0.040) | 0.499 (0.343) | 0.501 (0.191) | 0.370 (0.119) |
|  |  | **80** | 0.689 (0.035) | 0.861 (0.017) | 0.861 (0.017) | 0.848 (0.018) | 0.251 (0.039) | 0.440 (0.346) | 0.417 (0.263) | 0.358 (0.126) |
|  |  | **40** | 0.662 (0.044) | 0.861 (0.017) | 0.862 (0.017) | 0.848 (0.018) | 0.231 (0.040) | 0.407 (0.320) | 0.357 (0.318) | 0.351 (0.140) |
|  |  | **30** | 0.654 (0.052) | 0.860 (0.017) | 0.863 (0.016) | 0.849 (0.018) | 0.225 (0.040) | 0.361 (0.283) | 0.356 (0.346) | 0.338 (0.143) |
|  |  | **20** | 0.637 (0.071) | 0.853 (0.018) | 0.863 (0.017) | 0.852 (0.018) | 0.217 (0.046) | 0.289 (0.208) | 0.270 (0.353) | 0.327 (0.174) |
|  |  | **10** | 0.616 (0.109) | 0.837 (0.019) | 0.863 (0.016) | 0.857 (0.017) | 0.204 (0.057) | 0.225 (0.126) | 0.093 (0.246) | 0.264 (0.269) |

## Average AUCs of classifiers and frailty scale (Figure A9)

**Figure A9: Average AUCs of classifiers and frailty scale for 12-month mortality prediction on imbalanced data set**


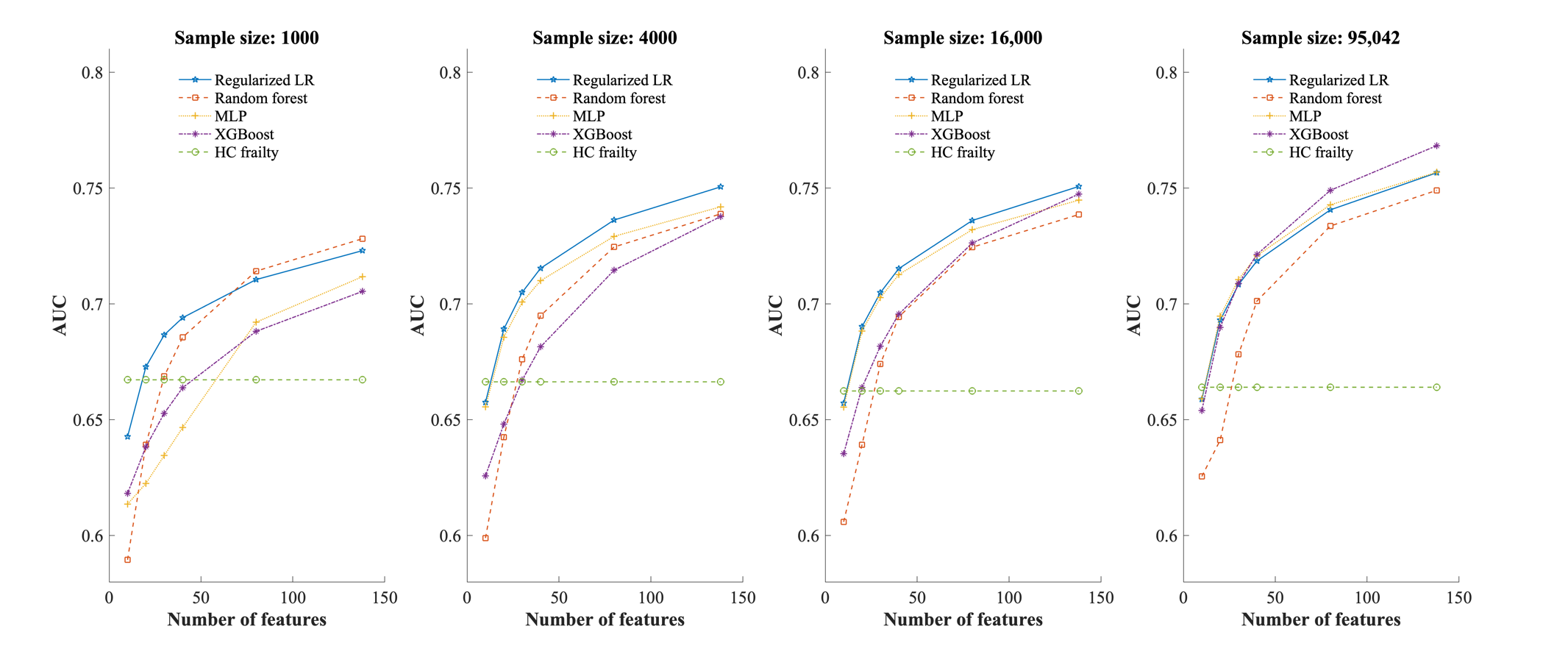
 Regularized LR=Regularized Logistic Regression. MLP=Multilayer Perceptron. XGBoost=eXtreme Gradient Boosting. HC Frailty=interRAI Home Care Frailty Scale.

## Average sensitivities of classifiers and frailty scale (Figure A10)

**Figure A10: Average sensitivities of classifiers and frailty scale for 12-month mortality prediction on imbalanced data set**


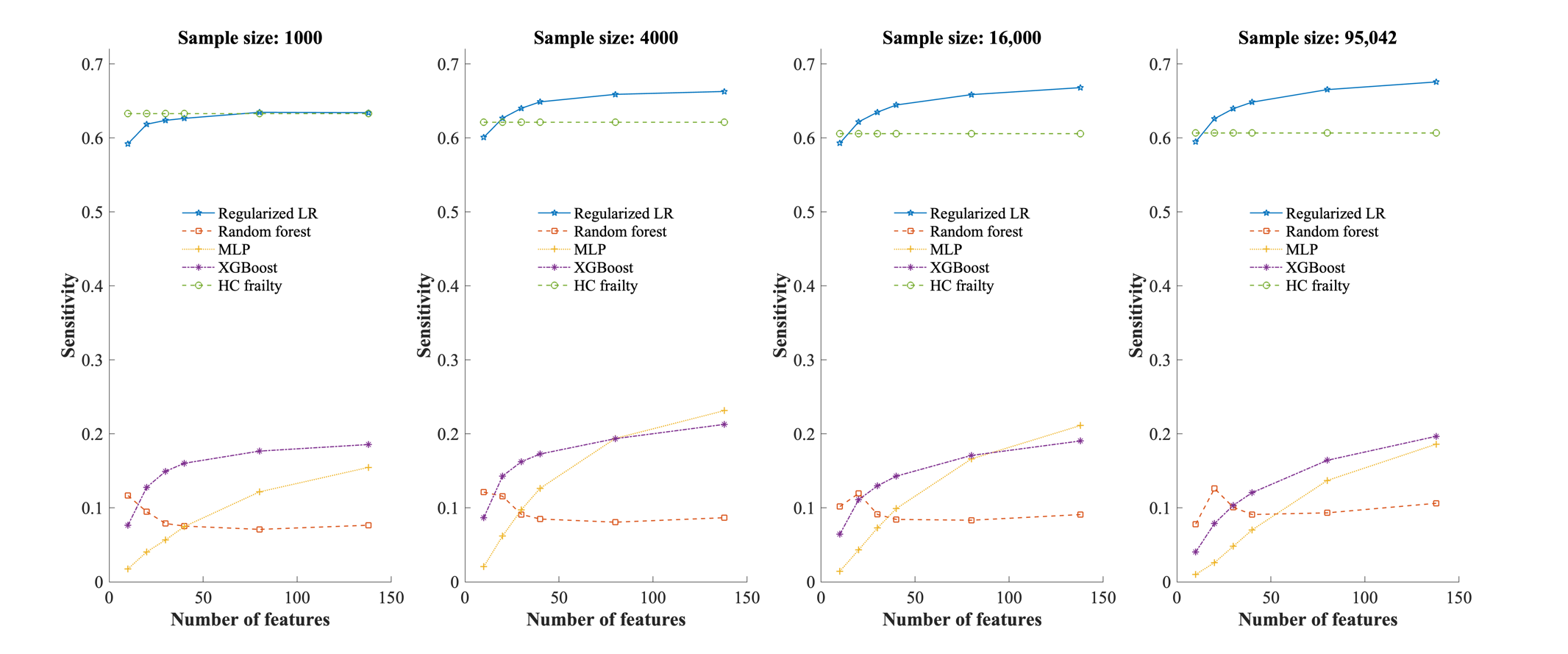
 Regularized LR=Regularized Logistic Regression. MLP=Multilayer Perceptron. XGBoost=eXtreme Gradient Boosting. HC Frailty=interRAI Home Care Frailty Scale.

## Average specificities of classifiers and frailty scale (Figure A11)

**Figure A11: Average specificities classifiers and frailty scale for 12-month mortality prediction on imbalanced data set**


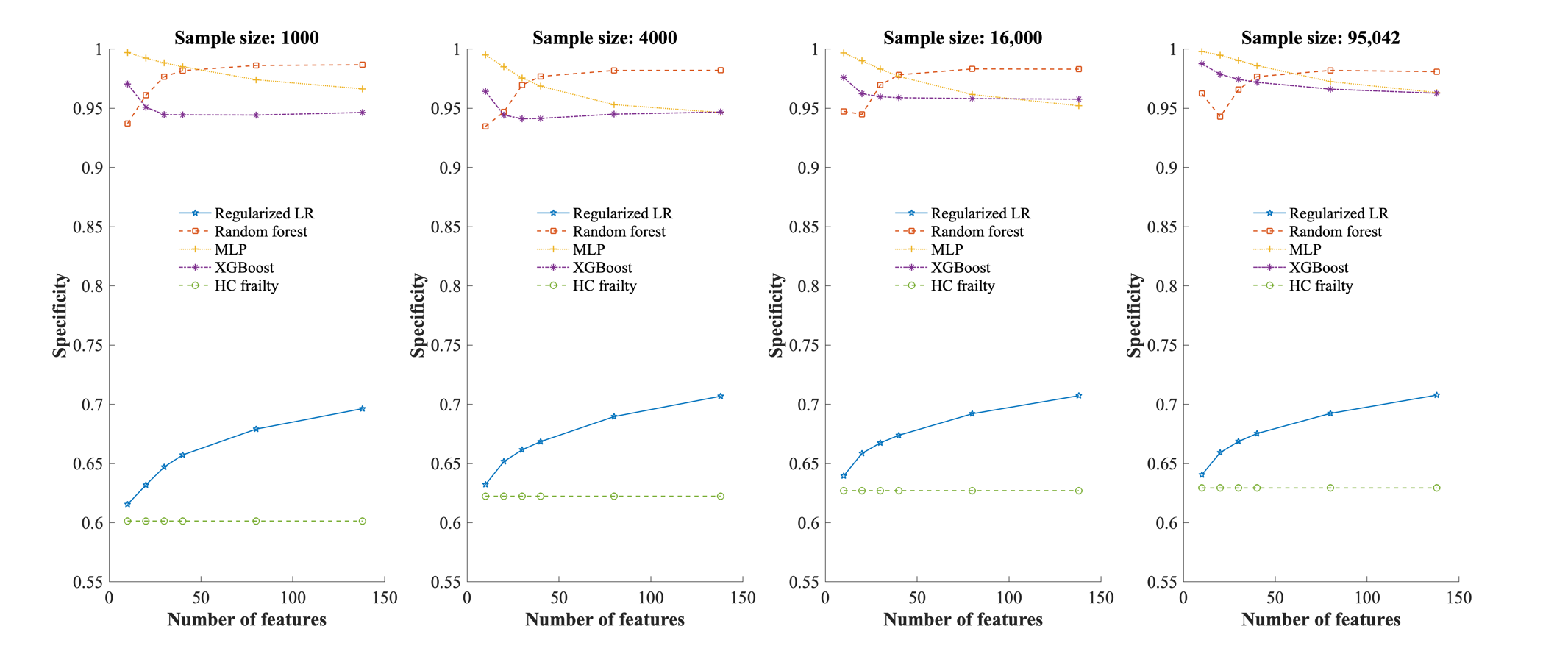
Regularized LR=Regularized Logistic Regression. MLP=Multilayer Perceptron. XGBoost=eXtreme Gradient Boosting. HC Frailty=interRAI Home Care Frailty Scale.

## Average AUCs, sensitivities, and specificities of classifiers by test sizes (Figure A12)

**Figure A12: Average AUCs, sensitivities, and specificities of classifiers for 12-month mortality prediction by test sizes on imbalanced data set**


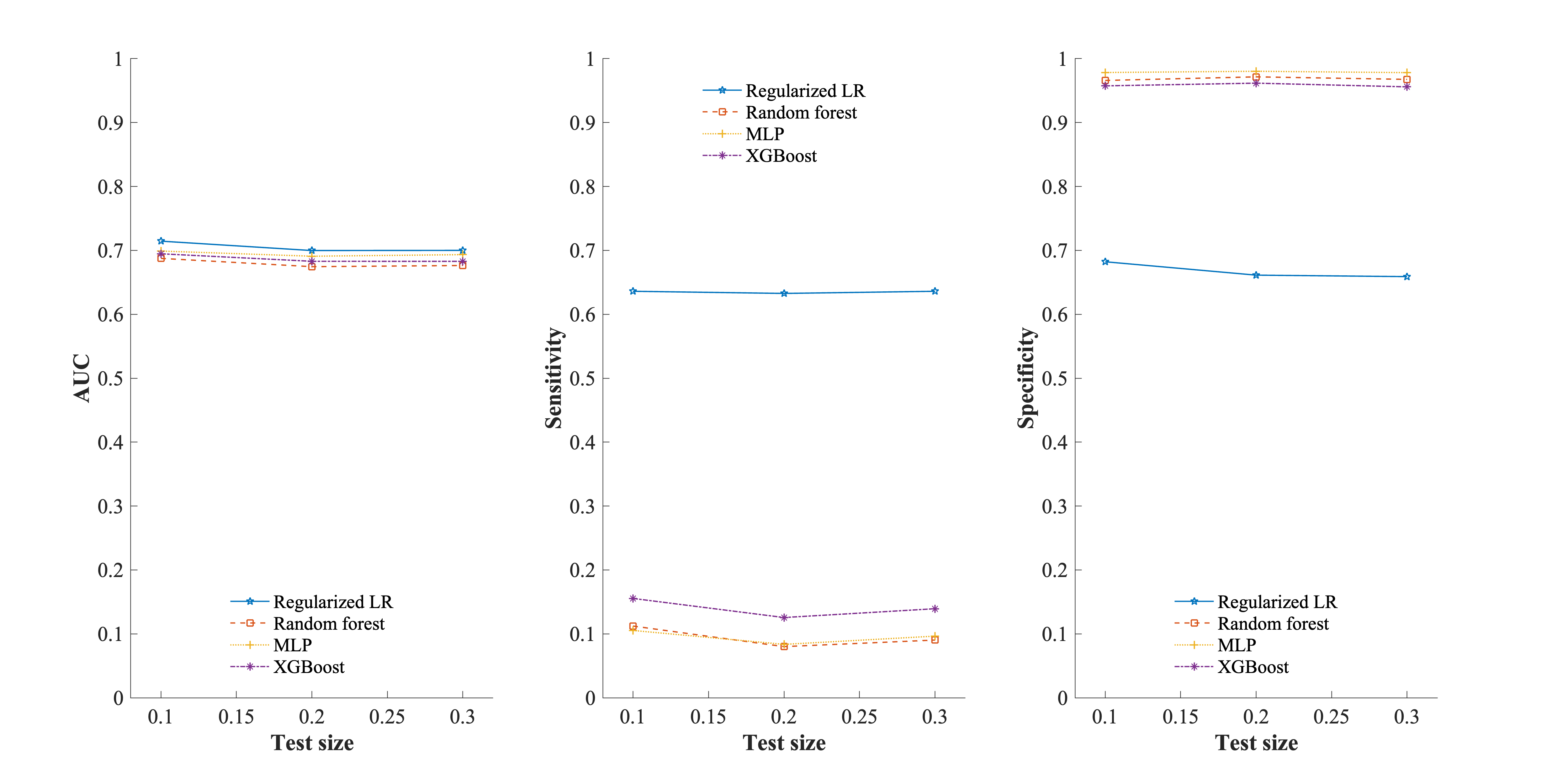
Regularized LR=Regularized Logistic Regression. RF=Random Forest. MLP=Multilayer Perceptron. XGBoost=eXtreme Gradient Boosting.

# 12-month mortality prediction on imbalanced data set

## Performance evaluation of frailty scale (Table A49 & A50)

**Table A49:**

**Average AUCs, sensitivities, and specificities of frailty scale for 12-month mortality prediction on imbalanced data set**

| **Sample Size** | **interRAI Home Care Frailty** | | |
| --- | --- | --- | --- |
|  | **Average AUC (SD)** | **Average Sensitivity (SD)** | **Average Specificity (SD)** |
| **1000** | 0.667 (0.011) | 0.633 (0.019) | 0.601 (0.022) |
| **4000** | 0.666 (0.009) | 0.621 (0.021) | 0.622 (0.001) |
| **16000** | 0.662 (0.003) | 0.606 (0.009) | 0.627 (0.003) |
| **95042** | 0.664 (0.000) | 0.607 (0.000) | 0.629 (0.000) |

SD=Standard Deviation

**Table A50:**

**Average accuracies, precisions, and F1-scores of frailty scale for 12-month mortality prediction on imbalanced data set**

| **Sample Size** | **interRAI Home Care Frailty** | | |
| --- | --- | --- | --- |
|  | **Average Accuracy (SD)** | **Average Precision (SD)** | **Average F1-score (SD)** |
| **1000** | 0.607 (0.021) | 0.255 (0.007) | 0.364 (0.005) |
| **4000** | 0.622 (0.005) | 0.294 (0.005) | 0.399 (0.009) |
| **16000** | 0.623 (0.001) | 0.289 (0.002) | 0.392 (0.003) |
| **95042** | 0.625 (0.000) | 0.287 (0.000) | 0.390 (0.000) |

SD=Standard Deviation

## Average AUCs of classifiers – test size 0.1 (Table A51)

**Table A51:**

**Average AUCs of classifiers for 12-month mortality prediction on imbalanced data set (test size 0.1)**

| **Test Size** | **Sample Size** | **Number of features** | **Average AUC** | | | |
| --- | --- | --- | --- | --- | --- | --- |
|  |  |  | **RLR** | **RF** | **MLP** | **XGBoost** |
| **0.1** | **95042** | **138** | 0.757 (0.006) | 0.749 (0.006) | 0.757 (0.006) | 0.769 (0.006) |
|  |  | **80** | 0.741 (0.008) | 0.734 (0.010) | 0.743 (0.009) | 0.750 (0.009) |
|  |  | **40** | 0.718 (0.012) | 0.701 (0.016) | 0.721 (0.013) | 0.722 (0.013) |
|  |  | **30** | 0.708 (0.013) | 0.677 (0.019) | 0.710 (0.015) | 0.709 (0.015) |
|  |  | **20** | 0.693 (0.017) | 0.642 (0.019) | 0.695 (0.018) | 0.691 (0.018) |
|  |  | **10** | 0.659 (0.030) | 0.627 (0.029) | 0.660 (0.030) | 0.655 (0.030) |
|  | **16000** | **138** | 0.752 (0.013) | 0.742 (0.014) | 0.748 (0.014) | 0.751 (0.013) |
|  |  | **80** | 0.738 (0.016) | 0.729 (0.017) | 0.735 (0.016) | 0.730 (0.016) |
|  |  | **40** | 0.718 (0.018) | 0.699 (0.020) | 0.716 (0.018) | 0.700 (0.019) |
|  |  | **30** | 0.708 (0.019) | 0.678 (0.023) | 0.706 (0.020) | 0.687 (0.021) |
|  |  | **20** | 0.693 (0.022) | 0.643 (0.026) | 0.692 (0.023) | 0.669 (0.024) |
|  |  | **10** | 0.658 (0.033) | 0.608 (0.030) | 0.656 (0.033) | 0.638 (0.032) |
|  | **4000** | **138** | 0.761 (0.027) | 0.745 (0.028) | 0.752 (0.029) | 0.742 (0.027) |
|  |  | **80** | 0.746 (0.029) | 0.730 (0.030) | 0.737 (0.030) | 0.720 (0.030) |
|  |  | **40** | 0.724 (0.031) | 0.701 (0.034) | 0.716 (0.032) | 0.688 (0.034) |
|  |  | **30** | 0.713 (0.033) | 0.682 (0.035) | 0.707 (0.035) | 0.674 (0.035) |
|  |  | **20** | 0.695 (0.036) | 0.646 (0.039) | 0.690 (0.037) | 0.654 (0.039) |
|  |  | **10** | 0.664 (0.046) | 0.605 (0.043) | 0.661 (0.046) | 0.633 (0.045) |
|  | **1000** | **138** | 0.764 (0.061) | 0.755 (0.062) | 0.727 (0.072) | 0.727 (0.064) |
|  |  | **80** | 0.745 (0.066) | 0.740 (0.065) | 0.710 (0.078) | 0.709 (0.068) |
|  |  | **40** | 0.722 (0.067) | 0.710 (0.072) | 0.660 (0.109) | 0.685 (0.073) |
|  |  | **30** | 0.714 (0.074) | 0.691 (0.074) | 0.639 (0.128) | 0.673 (0.076) |
|  |  | **20** | 0.695 (0.076) | 0.661 (0.076) | 0.625 (0.126) | 0.657 (0.077) |
|  |  | **10** | 0.661 (0.085) | 0.607 (0.084) | 0.611 (0.121) | 0.638 (0.083) |

RLR=Regularized Logistic Regression. RF=Random Forest. MLP=Multilayer Perceptron. XGBoost=eXtreme Gradient Boosting. SD=Standard Deviation

## Average AUCs of classifiers – test size 0.2 (Table A52)

**Table A52:**

**Average AUCs of classifiers for 12-month mortality prediction on imbalanced data set (test size 0.2)**

| **Test Size** | **Sample Size** | **Number of features** | **Average AUC** | | | |
| --- | --- | --- | --- | --- | --- | --- |
|  |  |  | **RLR** | **RF** | **MLP** | **XGBoost** |
| **0.2** | **95042** | **138** | 0.757 (0.004) | 0.749 (0.004) | 0.757 (0.004) | 0.769 (0.004) |
|  |  | **80** | 0.741 (0.007) | 0.734 (0.009) | 0.743 (0.008) | 0.749 (0.008) |
|  |  | **40** | 0.718 (0.011) | 0.701 (0.015) | 0.720 (0.013) | 0.721 (0.012) |
|  |  | **30** | 0.709 (0.012) | 0.679 (0.018) | 0.711 (0.014) | 0.710 (0.013) |
|  |  | **20** | 0.692 (0.017) | 0.640 (0.020) | 0.694 (0.018) | 0.689 (0.018) |
|  |  | **10** | 0.659 (0.028) | 0.626 (0.027) | 0.659 (0.028) | 0.654 (0.027) |
|  | **16000** | **138** | 0.747 (0.010) | 0.735 (0.010) | 0.740 (0.010) | 0.747 (0.009) |
|  |  | **80** | 0.732 (0.012) | 0.720 (0.013) | 0.727 (0.012) | 0.724 (0.013) |
|  |  | **40** | 0.710 (0.014) | 0.689 (0.017) | 0.707 (0.015) | 0.691 (0.016) |
|  |  | **30** | 0.700 (0.015) | 0.669 (0.020) | 0.698 (0.016) | 0.678 (0.017) |
|  |  | **20** | 0.685 (0.019) | 0.634 (0.023) | 0.682 (0.020) | 0.659 (0.020) |
|  |  | **10** | 0.653 (0.029) | 0.603 (0.026) | 0.652 (0.029) | 0.631 (0.028) |
|  | **4000** | **138** | 0.739 (0.019) | 0.730 (0.019) | 0.736 (0.020) | 0.728 (0.018) |
|  |  | **80** | 0.726 (0.020) | 0.716 (0.021) | 0.723 (0.022) | 0.706 (0.021) |
|  |  | **40** | 0.707 (0.024) | 0.688 (0.025) | 0.704 (0.024) | 0.674 (0.025) |
|  |  | **30** | 0.697 (0.025) | 0.670 (0.028) | 0.695 (0.025) | 0.661 (0.027) |
|  |  | **20** | 0.683 (0.028) | 0.638 (0.031) | 0.681 (0.028) | 0.643 (0.030) |
|  |  | **10** | 0.651 (0.037) | 0.594 (0.034) | 0.649 (0.037) | 0.619 (0.036) |
|  | **1000** | **138** | 0.708 (0.044) | 0.715 (0.042) | 0.706 (0.048) | 0.700 (0.042) |
|  |  | **80** | 0.702 (0.047) | 0.703 (0.045) | 0.685 (0.063) | 0.681 (0.045) |
|  |  | **40** | 0.689 (0.051) | 0.677 (0.051) | 0.643 (0.107) | 0.659 (0.053) |
|  |  | **30** | 0.680 (0.051) | 0.662 (0.053) | 0.628 (0.111) | 0.648 (0.055) |
|  |  | **20** | 0.669 (0.051) | 0.633 (0.053) | 0.622 (0.107) | 0.634 (0.055) |
|  |  | **10** | 0.641 (0.060) | 0.585 (0.061) | 0.619 (0.095) | 0.615 (0.059) |

Regularized LR=Regularized Logistic Regression. RF=Random Forest. MLP=Multilayer Perceptron. XGBoost=eXtreme Gradient Boosting. SD=Standard Deviation

## Average AUCs of classifiers – test size 0.3 (Table A53)

**Table A53:**

**Average AUCs of classifiers for 12-month mortality prediction on imbalanced data set (test size 0.3)**

| **Test Size** | **Sample Size** | **Number of features** | **Average AUC** | | | |
| --- | --- | --- | --- | --- | --- | --- |
|  |  |  | **LR** | **RF** | **MLP** | **XGBoost** |
| **0.3** | **95042** | **138** | 0.757 (0.003) | 0.749 (0.003) | 0.756 (0.003) | 0.767 (0.003) |
|  |  | **80** | 0.741 (0.007) | 0.733 (0.009) | 0.742 (0.008) | 0.748 (0.008) |
|  |  | **40** | 0.719 (0.011) | 0.702 (0.015) | 0.721 (0.012) | 0.721 (0.012) |
|  |  | **30** | 0.708 (0.012) | 0.678 (0.018) | 0.710 (0.014) | 0.708 (0.014) |
|  |  | **20** | 0.693 (0.016) | 0.641 (0.019) | 0.695 (0.017) | 0.689 (0.016) |
|  |  | **10** | 0.658 (0.030) | 0.624 (0.028) | 0.658 (0.029) | 0.653 (0.029) |
|  | **16000** | **138** | 0.753 (0.007) | 0.739 (0.008) | 0.747 (0.008) | 0.745 (0.007) |
|  |  | **80** | 0.738 (0.010) | 0.725 (0.011) | 0.735 (0.010) | 0.725 (0.011) |
|  |  | **40** | 0.718 (0.013) | 0.695 (0.016) | 0.715 (0.013) | 0.695 (0.014) |
|  |  | **30** | 0.707 (0.015) | 0.675 (0.019) | 0.705 (0.015) | 0.681 (0.016) |
|  |  | **20** | 0.693 (0.018) | 0.641 (0.022) | 0.690 (0.019) | 0.664 (0.019) |
|  |  | **10** | 0.660 (0.030) | 0.607 (0.025) | 0.658 (0.030) | 0.637 (0.029) |
|  | **4000** | **138** | 0.752 (0.014) | 0.742 (0.015) | 0.739 (0.015) | 0.743 (0.014) |
|  |  | **80** | 0.737 (0.018) | 0.728 (0.017) | 0.728 (0.018) | 0.718 (0.018) |
|  |  | **40** | 0.716 (0.021) | 0.696 (0.023) | 0.710 (0.021) | 0.683 (0.022) |
|  |  | **30** | 0.705 (0.022) | 0.677 (0.024) | 0.700 (0.021) | 0.666 (0.024) |
|  |  | **20** | 0.689 (0.026) | 0.643 (0.030) | 0.686 (0.026) | 0.647 (0.027) |
|  |  | **10** | 0.658 (0.035) | 0.598 (0.032) | 0.657 (0.033) | 0.625 (0.033) |
|  | **1000** | **138** | 0.697 (0.032) | 0.715 (0.031) | 0.702 (0.033) | 0.689 (0.029) |
|  |  | **80** | 0.685 (0.035) | 0.699 (0.032) | 0.681 (0.047) | 0.674 (0.035) |
|  |  | **40** | 0.672 (0.037) | 0.670 (0.038) | 0.637 (0.091) | 0.648 (0.039) |
|  |  | **30** | 0.665 (0.038) | 0.654 (0.040) | 0.636 (0.086) | 0.638 (0.041) |
|  |  | **20** | 0.654 (0.042) | 0.624 (0.046) | 0.621 (0.092) | 0.624 (0.045) |
|  |  | **10** | 0.625 (0.052) | 0.577 (0.050) | 0.611 (0.078) | 0.601 (0.052) |

RLR=Regularized Logistic Regression. RF=Random Forest. MLP=Multilayer Perceptron. XGBoost=eXtreme Gradient Boosting. SD=Standard Deviation

## Average sensitivities and specificities of classifiers – test size 0.1 (Table A54)

**Table A54: Average sensitivities and specificities of classifiers for 12-month mortality prediction on imbalanced data set (test size 0.1)**

| **Test Size** | **Sample Size** | **Number of features** | **Average Sensitivity (SD)** | | | | **Average Specificity (SD)** | | | |
| --- | --- | --- | --- | --- | --- | --- | --- | --- | --- | --- |
|  |  |  | **LR** | **RF** | **MLP** | **XGBoost** | **LR** | **RF** | **MLP** | **XGBoost** |
| **0.1** | **95042** | **138** | 0.676 (0.011) | 0.108 (0.007) | 0.187 (0.039) | 0.197 (0.008) | 0.708 (0.005) | 0.981 (0.002) | 0.963 (0.011) | 0.963 (0.002) |
|  |  | **80** | 0.665 (0.013) | 0.094 (0.022) | 0.134 (0.038) | 0.164 (0.020) | 0.692 (0.011) | 0.982 (0.004) | 0.973 (0.009) | 0.966 (0.003) |
|  |  | **40** | 0.648 (0.020) | 0.092 (0.028) | 0.068 (0.043) | 0.120 (0.030) | 0.675 (0.019) | 0.977 (0.005) | 0.987 (0.008) | 0.972 (0.005) |
|  |  | **30** | 0.639 (0.026) | 0.101 (0.029) | 0.045 (0.041) | 0.101 (0.032) | 0.668 (0.023) | 0.965 (0.009) | 0.991 (0.008) | 0.975 (0.006) |
|  |  | **20** | 0.627 (0.036) | 0.126 (0.026) | 0.025 (0.036) | 0.077 (0.033) | 0.659 (0.030) | 0.943 (0.009) | 0.995 (0.007) | 0.980 (0.006) |
|  |  | **10** | 0.593 (0.073) | 0.078 (0.051) | 0.010 (0.026) | 0.039 (0.030) | 0.643 (0.059) | 0.962 (0.034) | 0.998 (0.005) | 0.988 (0.007) |
|  | **16000** | **138** | 0.667 (0.025) | 0.094 (0.015) | 0.206 (0.054) | 0.186 (0.021) | 0.709 (0.012) | 0.982 (0.004) | 0.954 (0.016) | 0.957 (0.006) |
|  |  | **80** | 0.659 (0.027) | 0.086 (0.024) | 0.162 (0.052) | 0.169 (0.026) | 0.695 (0.016) | 0.982 (0.005) | 0.962 (0.014) | 0.958 (0.006) |
|  |  | **40** | 0.645 (0.032) | 0.086 (0.029) | 0.095 (0.049) | 0.142 (0.028) | 0.678 (0.022) | 0.977 (0.006) | 0.978 (0.012) | 0.959 (0.007) |
|  |  | **30** | 0.636 (0.033) | 0.094 (0.029) | 0.071 (0.046) | 0.130 (0.031) | 0.671 (0.026) | 0.969 (0.009) | 0.983 (0.010) | 0.960 (0.007) |
|  |  | **20** | 0.623 (0.045) | 0.123 (0.032) | 0.042 (0.040) | 0.111 (0.033) | 0.662 (0.034) | 0.943 (0.013) | 0.990 (0.009) | 0.963 (0.008) |
|  |  | **10** | 0.590 (0.077) | 0.102 (0.046) | 0.014 (0.029) | 0.063 (0.034) | 0.644 (0.065) | 0.948 (0.028) | 0.997 (0.006) | 0.976 (0.010) |
|  | **4000** | **138** | 0.671 (0.053) | 0.097 (0.030) | 0.236 (0.078) | 0.218 (0.044) | 0.709 (0.024) | 0.980 (0.008) | 0.946 (0.024) | 0.946 (0.013) |
|  |  | **80** | 0.669 (0.051) | 0.087 (0.034) | 0.191 (0.066) | 0.193 (0.044) | 0.694 (0.029) | 0.980 (0.008) | 0.954 (0.020) | 0.945 (0.014) |
|  |  | **40** | 0.656 (0.055) | 0.093 (0.038) | 0.129 (0.059) | 0.173 (0.047) | 0.673 (0.032) | 0.975 (0.009) | 0.968 (0.016) | 0.942 (0.014) |
|  |  | **30** | 0.645 (0.060) | 0.099 (0.040) | 0.100 (0.054) | 0.165 (0.046) | 0.669 (0.037) | 0.968 (0.012) | 0.975 (0.014) | 0.942 (0.015) |
|  |  | **20** | 0.629 (0.066) | 0.123 (0.045) | 0.069 (0.051) | 0.145 (0.047) | 0.658 (0.045) | 0.945 (0.017) | 0.983 (0.012) | 0.945 (0.014) |
|  |  | **10** | 0.602 (0.105) | 0.122 (0.051) | 0.023 (0.035) | 0.088 (0.047) | 0.640 (0.081) | 0.936 (0.026) | 0.994 (0.008) | 0.964 (0.017) |
|  | **1000** | **138** | 0.653 (0.113) | 0.166 (0.083) | 0.224 (0.125) | 0.277 (0.099) | 0.746 (0.050) | 0.979 (0.016) | 0.962 (0.028) | 0.945 (0.025) |
|  |  | **80** | 0.647 (0.113) | 0.149 (0.085) | 0.187 (0.123) | 0.252 (0.105) | 0.728 (0.052) | 0.978 (0.017) | 0.970 (0.025) | 0.942 (0.027) |
|  |  | **40** | 0.622 (0.118) | 0.141 (0.092) | 0.119 (0.112) | 0.222 (0.102) | 0.709 (0.062) | 0.974 (0.019) | 0.983 (0.019) | 0.941 (0.027) |
|  |  | **30** | 0.619 (0.130) | 0.135 (0.089) | 0.088 (0.108) | 0.203 (0.100) | 0.698 (0.068) | 0.969 (0.021) | 0.987 (0.019) | 0.940 (0.027) |
|  |  | **20** | 0.605 (0.133) | 0.144 (0.091) | 0.069 (0.096) | 0.176 (0.099) | 0.685 (0.084) | 0.954 (0.025) | 0.991 (0.015) | 0.945 (0.027) |
|  |  | **10** | 0.577 (0.163) | 0.151 (0.100) | 0.034 (0.074) | 0.117 (0.099) | 0.659 (0.130) | 0.933 (0.033) | 0.996 (0.010) | 0.963 (0.025) |

## Average sensitivities and specificities of classifiers – test size 0.2 (Table A55)

**Table A55: Average sensitivities and specificities of classifiers for 12-month mortality prediction on imbalanced data set (test size 0.2)**

| **Test Size** | **Sample Size** | **Number of features** | **Average Sensitivity (SD)** | | | | **Average Specificity (SD)** | | | |
| --- | --- | --- | --- | --- | --- | --- | --- | --- | --- | --- |
|  |  |  | **RLR** | **RF** | **MLP** | **XGBoost** | **RLR** | **RF** | **MLP** | **XGBoost** |
| **0.2** | **95042** | **138** | 0.676 (0.008) | 0.106 (0.005) | 0.184 (0.042) | 0.197 (0.006) | 0.708 (0.004) | 0.981 (0.001) | 0.964 (0.011) | 0.963 (0.002) |
|  |  | **80** | 0.665 (0.011) | 0.093 (0.021) | 0.137 (0.043) | 0.164 (0.020) | 0.692 (0.011) | 0.982 (0.003) | 0.973 (0.010) | 0.966 (0.003) |
|  |  | **40** | 0.648 (0.019) | 0.090 (0.027) | 0.069 (0.043) | 0.119 (0.029) | 0.675 (0.018) | 0.977 (0.005) | 0.986 (0.008) | 0.972 (0.005) |
|  |  | **30** | 0.640 (0.024) | 0.102 (0.028) | 0.049 (0.043) | 0.104 (0.031) | 0.669 (0.022) | 0.966 (0.009) | 0.990 (0.008) | 0.974 (0.005) |
|  |  | **20** | 0.626 (0.036) | 0.125 (0.026) | 0.024 (0.033) | 0.078 (0.032) | 0.659 (0.032) | 0.943 (0.010) | 0.995 (0.006) | 0.979 (0.006) |
|  |  | **10** | 0.594 (0.070) | 0.077 (0.046) | 0.010 (0.027) | 0.041 (0.030) | 0.641 (0.061) | 0.964 (0.031) | 0.998 (0.005) | 0.988 (0.006) |
|  | **16000** | **138** | 0.672 (0.018) | 0.088 (0.011) | 0.210 (0.052) | 0.193 (0.014) | 0.702 (0.009) | 0.984 (0.003) | 0.952 (0.016) | 0.958 (0.004) |
|  |  | **80** | 0.656 (0.020) | 0.081 (0.018) | 0.164 (0.048) | 0.172 (0.021) | 0.687 (0.014) | 0.984 (0.004) | 0.962 (0.014) | 0.959 (0.005) |
|  |  | **40** | 0.643 (0.025) | 0.081 (0.024) | 0.095 (0.043) | 0.141 (0.026) | 0.667 (0.019) | 0.979 (0.005) | 0.978 (0.011) | 0.959 (0.006) |
|  |  | **30** | 0.633 (0.029) | 0.089 (0.026) | 0.069 (0.040) | 0.128 (0.027) | 0.661 (0.023) | 0.970 (0.008) | 0.984 (0.010) | 0.960 (0.006) |
|  |  | **20** | 0.619 (0.041) | 0.117 (0.028) | 0.041 (0.035) | 0.109 (0.028) | 0.653 (0.032) | 0.945 (0.011) | 0.990 (0.008) | 0.963 (0.007) |
|  |  | **10** | 0.593 (0.074) | 0.099 (0.042) | 0.012 (0.023) | 0.062 (0.031) | 0.633 (0.065) | 0.948 (0.028) | 0.997 (0.005) | 0.977 (0.010) |
|  | **4000** | **138** | 0.646 (0.036) | 0.068 (0.018) | 0.215 (0.070) | 0.193 (0.029) | 0.701 (0.019) | 0.984 (0.006) | 0.946 (0.021) | 0.945 (0.010) |
|  |  | **80** | 0.644 (0.038) | 0.068 (0.023) | 0.182 (0.061) | 0.179 (0.030) | 0.684 (0.023) | 0.983 (0.006) | 0.952 (0.019) | 0.944 (0.010) |
|  |  | **40** | 0.637 (0.043) | 0.075 (0.029) | 0.118 (0.052) | 0.163 (0.034) | 0.667 (0.029) | 0.978 (0.008) | 0.968 (0.014) | 0.941 (0.011) |
|  |  | **30** | 0.628 (0.047) | 0.084 (0.031) | 0.092 (0.049) | 0.154 (0.035) | 0.660 (0.035) | 0.970 (0.010) | 0.976 (0.013) | 0.941 (0.011) |
|  |  | **20** | 0.617 (0.056) | 0.110 (0.036) | 0.057 (0.044) | 0.138 (0.037) | 0.652 (0.045) | 0.947 (0.015) | 0.985 (0.011) | 0.944 (0.012) |
|  |  | **10** | 0.592 (0.095) | 0.118 (0.043) | 0.019 (0.033) | 0.084 (0.042) | 0.630 (0.083) | 0.935 (0.024) | 0.995 (0.007) | 0.965 (0.015) |
|  | **1000** | **138** | 0.617 (0.090) | 0.022 (0.027) | 0.101 (0.068) | 0.126 (0.056) | 0.685 (0.038) | 0.996 (0.005) | 0.975 (0.015) | 0.960 (0.016) |
|  |  | **80** | 0.627 (0.091) | 0.022 (0.029) | 0.070 (0.062) | 0.125 (0.058) | 0.666 (0.048) | 0.996 (0.006) | 0.982 (0.014) | 0.958 (0.017) |
|  |  | **40** | 0.634 (0.096) | 0.031 (0.034) | 0.037 (0.046) | 0.112 (0.061) | 0.640 (0.057) | 0.991 (0.008) | 0.990 (0.010) | 0.959 (0.017) |
|  |  | **30** | 0.634 (0.101) | 0.036 (0.037) | 0.026 (0.040) | 0.103 (0.060) | 0.629 (0.066) | 0.988 (0.010) | 0.993 (0.009) | 0.960 (0.017) |
|  |  | **20** | 0.631 (0.109) | 0.054 (0.045) | 0.016 (0.031) | 0.086 (0.057) | 0.613 (0.077) | 0.974 (0.016) | 0.996 (0.007) | 0.965 (0.017) |
|  |  | **10** | 0.611 (0.159) | 0.084 (0.058) | 0.006 (0.019) | 0.043 (0.045) | 0.597 (0.126) | 0.952 (0.021) | 0.999 (0.005) | 0.981 (0.015) |

## Average sensitivities and specificities of classifiers – test size 0.3 (Table A56)

**Table A56: Average sensitivities and specificities of classifiers for 12-month mortality prediction on imbalanced data set (test size 0.3)**

| **Test Size** | **Sample Size** | **Number of features** | **Average Sensitivity (SD)** | | | | **Average Specificity (SD)** | | | |
| --- | --- | --- | --- | --- | --- | --- | --- | --- | --- | --- |
|  |  |  | **RLR** | **RF** | **MLP** | **XGBoost** | **RLR** | **RF** | **MLP** | **XGBoost** |
| **0.3** | **95042** | **138** | 0.675 (0.006) | 0.105 (0.004) | 0.187 (0.040) | 0.196 (0.005) | 0.708 (0.003) | 0.981 (0.001) | 0.963 (0.011) | 0.962 (0.002) |
|  |  | **80** | 0.665 (0.010) | 0.092 (0.021) | 0.140 (0.042) | 0.165 (0.019) | 0.692 (0.011) | 0.982 (0.003) | 0.972 (0.010) | 0.966 (0.003) |
|  |  | **40** | 0.649 (0.018) | 0.091 (0.027) | 0.074 (0.044) | 0.122 (0.029) | 0.675 (0.018) | 0.977 (0.005) | 0.985 (0.009) | 0.971 (0.005) |
|  |  | **30** | 0.639 (0.024) | 0.100 (0.028) | 0.049 (0.041) | 0.105 (0.030) | 0.669 (0.022) | 0.966 (0.009) | 0.990 (0.008) | 0.974 (0.005) |
|  |  | **20** | 0.625 (0.036) | 0.127 (0.026) | 0.028 (0.037) | 0.082 (0.032) | 0.660 (0.031) | 0.943 (0.010) | 0.994 (0.007) | 0.978 (0.006) |
|  |  | **10** | 0.598 (0.073) | 0.079 (0.050) | 0.010 (0.025) | 0.041 (0.030) | 0.637 (0.063) | 0.961 (0.034) | 0.998 (0.005) | 0.987 (0.007) |
|  | **16000** | **138** | 0.665 (0.015) | 0.090 (0.009) | 0.218 (0.051) | 0.192 (0.013) | 0.711 (0.008) | 0.984 (0.003) | 0.951 (0.016) | 0.958 (0.004) |
|  |  | **80** | 0.660 (0.017) | 0.083 (0.020) | 0.173 (0.047) | 0.172 (0.020) | 0.694 (0.014) | 0.984 (0.004) | 0.961 (0.014) | 0.958 (0.004) |
|  |  | **40** | 0.645 (0.024) | 0.086 (0.025) | 0.107 (0.045) | 0.146 (0.024) | 0.677 (0.020) | 0.979 (0.005) | 0.976 (0.011) | 0.958 (0.005) |
|  |  | **30** | 0.636 (0.028) | 0.090 (0.026) | 0.079 (0.043) | 0.131 (0.027) | 0.670 (0.025) | 0.970 (0.008) | 0.982 (0.010) | 0.959 (0.005) |
|  |  | **20** | 0.623 (0.039) | 0.118 (0.026) | 0.046 (0.038) | 0.113 (0.028) | 0.661 (0.033) | 0.946 (0.011) | 0.990 (0.008) | 0.962 (0.007) |
|  |  | **10** | 0.596 (0.072) | 0.105 (0.043) | 0.017 (0.030) | 0.068 (0.033) | 0.641 (0.063) | 0.946 (0.027) | 0.996 (0.006) | 0.975 (0.010) |
|  | **4000** | **138** | 0.671 (0.030) | 0.095 (0.020) | 0.243 (0.064) | 0.228 (0.026) | 0.710 (0.015) | 0.983 (0.005) | 0.947 (0.020) | 0.949 (0.008) |
|  |  | **80** | 0.663 (0.034) | 0.087 (0.030) | 0.209 (0.059) | 0.209 (0.030) | 0.690 (0.022) | 0.983 (0.006) | 0.953 (0.018) | 0.947 (0.009) |
|  |  | **40** | 0.653 (0.039) | 0.087 (0.037) | 0.132 (0.055) | 0.182 (0.037) | 0.666 (0.029) | 0.978 (0.008) | 0.970 (0.014) | 0.942 (0.010) |
|  |  | **30** | 0.647 (0.043) | 0.091 (0.036) | 0.101 (0.051) | 0.168 (0.037) | 0.656 (0.031) | 0.971 (0.009) | 0.977 (0.012) | 0.941 (0.010) |
|  |  | **20** | 0.634 (0.054) | 0.113 (0.038) | 0.060 (0.048) | 0.146 (0.039) | 0.645 (0.043) | 0.949 (0.014) | 0.987 (0.010) | 0.944 (0.011) |
|  |  | **10** | 0.608 (0.092) | 0.123 (0.041) | 0.020 (0.036) | 0.089 (0.043) | 0.627 (0.072) | 0.933 (0.023) | 0.996 (0.006) | 0.964 (0.015) |
|  | **1000** | **138** | 0.633 (0.062) | 0.042 (0.025) | 0.140 (0.057) | 0.154 (0.047) | 0.658 (0.034) | 0.985 (0.009) | 0.962 (0.018) | 0.934 (0.019) |
|  |  | **80** | 0.629 (0.068) | 0.042 (0.027) | 0.108 (0.054) | 0.154 (0.049) | 0.642 (0.039) | 0.985 (0.010) | 0.971 (0.016) | 0.933 (0.018) |
|  |  | **40** | 0.623 (0.077) | 0.055 (0.035) | 0.068 (0.052) | 0.147 (0.050) | 0.622 (0.052) | 0.980 (0.012) | 0.982 (0.014) | 0.934 (0.019) |
|  |  | **30** | 0.619 (0.084) | 0.065 (0.040) | 0.056 (0.047) | 0.142 (0.053) | 0.615 (0.060) | 0.974 (0.013) | 0.985 (0.012) | 0.934 (0.020) |
|  |  | **20** | 0.620 (0.101) | 0.086 (0.047) | 0.036 (0.041) | 0.122 (0.052) | 0.598 (0.088) | 0.956 (0.019) | 0.991 (0.010) | 0.943 (0.020) |
|  |  | **10** | 0.588 (0.174) | 0.114 (0.053) | 0.013 (0.029) | 0.070 (0.050) | 0.590 (0.149) | 0.927 (0.025) | 0.997 (0.006) | 0.967 (0.020) |

## Average F1-scores of classifiers – test size 0.1 (Table A57)

**Table A57:**

**Average F1-scores of classifiers for 12-month mortality prediction on imbalanced data set (test size 0.1)**

| **Test Size** | **Sample Size** | **Number of features** | **Average F1-score (SD)** | | | |
| --- | --- | --- | --- | --- | --- | --- |
|  |  |  | **RLR** | **RF** | **MLP** | **XGBosst** |
| **0.1** | **95042** | **138** | 0.472 (0.008) | 0.182 (0.010) | 0.277 (0.042) | 0.293 (0.011) |
|  |  | **80** | 0.456 (0.009) | 0.161 (0.033) | 0.213 (0.051) | 0.252 (0.026) |
|  |  | **40** | 0.437 (0.012) | 0.153 (0.041) | 0.116 (0.068) | 0.193 (0.041) |
|  |  | **30** | 0.428 (0.013) | 0.161 (0.040) | 0.079 (0.067) | 0.166 (0.045) |
|  |  | **20** | 0.416 (0.015) | 0.185 (0.032) | 0.045 (0.059) | 0.131 (0.048) |
|  |  | **10** | 0.389 (0.026) | 0.120 (0.059) | 0.018 (0.044) | 0.070 (0.049) |
|  | **16000** | **138** | 0.472 (0.018) | 0.161 (0.023) | 0.292 (0.056) | 0.274 (0.026) |
|  |  | **80** | 0.459 (0.020) | 0.147 (0.037) | 0.243 (0.062) | 0.252 (0.033) |
|  |  | **40** | 0.441 (0.021) | 0.145 (0.043) | 0.156 (0.070) | 0.217 (0.037) |
|  |  | **30** | 0.432 (0.022) | 0.154 (0.041) | 0.120 (0.071) | 0.200 (0.041) |
|  |  | **20** | 0.420 (0.023) | 0.182 (0.040) | 0.073 (0.065) | 0.174 (0.045) |
|  |  | **10** | 0.392 (0.030) | 0.150 (0.055) | 0.024 (0.049) | 0.106 (0.052) |
|  | **4000** | **138** | 0.473 (0.039) | 0.164 (0.047) | 0.318 (0.078) | 0.303 (0.053) |
|  |  | **80** | 0.462 (0.037) | 0.148 (0.052) | 0.272 (0.075) | 0.271 (0.054) |
|  |  | **40** | 0.442 (0.039) | 0.154 (0.057) | 0.199 (0.079) | 0.244 (0.058) |
|  |  | **30** | 0.435 (0.040) | 0.158 (0.057) | 0.160 (0.078) | 0.234 (0.057) |
|  |  | **20** | 0.419 (0.041) | 0.181 (0.058) | 0.115 (0.078) | 0.210 (0.060) |
|  |  | **10** | 0.395 (0.047) | 0.174 (0.062) | 0.040 (0.058) | 0.139 (0.067) |
|  | **1000** | **138** | 0.469 (0.084) | 0.256 (0.112) | 0.303 (0.138) | 0.358 (0.109) |
|  |  | **80** | 0.452 (0.084) | 0.230 (0.117) | 0.263 (0.146) | 0.325 (0.115) |
|  |  | **40** | 0.426 (0.082) | 0.214 (0.126) | 0.179 (0.153) | 0.290 (0.117) |
|  |  | **30** | 0.418 (0.086) | 0.203 (0.120) | 0.134 (0.149) | 0.268 (0.117) |
|  |  | **20** | 0.403 (0.087) | 0.205 (0.116) | 0.107 (0.139) | 0.238 (0.119) |
|  |  | **10** | 0.372 (0.094) | 0.199 (0.119) | 0.054 (0.111) | 0.170 (0.132) |

RLR=Regularized Logistic Regression. RF=Random Forest. MLP=Multilayer Perceptron. XGBoost=eXtreme Gradient Boosting. SD=Standard Deviation

## Average F1-scores of classifiers – test size 0.2 (Table A58)

**Table A58:**

**Average F1-scores of classifiers for 12-month mortality prediction on imbalanced data set (test size 0.2)**

| **Test Size** | **Sample Size** | **Number of features** | **Average F1-score (SD)** | | | |
| --- | --- | --- | --- | --- | --- | --- |
|  |  |  | **RLR** | **RF** | **MLP** | **XGBosst** |
| **0.2** | **95042** | **138** | 0.472 (0.005) | 0.179 (0.007) | 0.274 (0.045) | 0.292 (0.007) |
|  |  | **80** | 0.457 (0.008) | 0.159 (0.033) | 0.216 (0.057) | 0.252 (0.026) |
|  |  | **40** | 0.437 (0.010) | 0.151 (0.040) | 0.118 (0.068) | 0.192 (0.040) |
|  |  | **30** | 0.429 (0.011) | 0.162 (0.038) | 0.086 (0.070) | 0.171 (0.044) |
|  |  | **20** | 0.416 (0.014) | 0.184 (0.031) | 0.044 (0.056) | 0.131 (0.047) |
|  |  | **10** | 0.389 (0.023) | 0.120 (0.055) | 0.018 (0.045) | 0.072 (0.049) |
|  | **16000** | **138** | 0.471 (0.013) | 0.153 (0.017) | 0.296 (0.052) | 0.284 (0.017) |
|  |  | **80** | 0.453 (0.014) | 0.141 (0.028) | 0.245 (0.055) | 0.256 (0.025) |
|  |  | **40** | 0.433 (0.016) | 0.138 (0.037) | 0.156 (0.063) | 0.215 (0.034) |
|  |  | **30** | 0.425 (0.016) | 0.146 (0.037) | 0.117 (0.062) | 0.198 (0.036) |
|  |  | **20** | 0.413 (0.019) | 0.174 (0.035) | 0.073 (0.058) | 0.172 (0.039) |
|  |  | **10** | 0.389 (0.025) | 0.148 (0.049) | 0.023 (0.041) | 0.106 (0.048) |
|  | **4000** | **138** | 0.459 (0.026) | 0.120 (0.030) | 0.295 (0.072) | 0.272 (0.035) |
|  |  | **80** | 0.449 (0.027) | 0.119 (0.037) | 0.260 (0.070) | 0.255 (0.037) |
|  |  | **40** | 0.435 (0.027) | 0.127 (0.045) | 0.185 (0.069) | 0.233 (0.042) |
|  |  | **30** | 0.426 (0.028) | 0.138 (0.046) | 0.149 (0.070) | 0.222 (0.044) |
|  |  | **20** | 0.416 (0.030) | 0.165 (0.047) | 0.098 (0.069) | 0.202 (0.046) |
|  |  | **10** | 0.391 (0.037) | 0.169 (0.051) | 0.034 (0.054) | 0.133 (0.059) |
|  | **1000** | **138** | 0.366 (0.054) | 0.040 (0.047) | 0.155 (0.095) | 0.183 (0.072) |
|  |  | **80** | 0.362 (0.055) | 0.039 (0.051) | 0.112 (0.092) | 0.179 (0.076) |
|  |  | **40** | 0.352 (0.053) | 0.054 (0.059) | 0.062 (0.075) | 0.163 (0.081) |
|  |  | **30** | 0.345 (0.056) | 0.062 (0.062) | 0.044 (0.066) | 0.151 (0.080) |
|  |  | **20** | 0.334 (0.054) | 0.086 (0.067) | 0.029 (0.053) | 0.130 (0.079) |
|  |  | **10** | 0.317 (0.064) | 0.121 (0.076) | 0.010 (0.033) | 0.070 (0.070) |

RLR=Regularized Logistic Regression. RF=Random Forest. MLP=Multilayer Perceptron. XGBoost=eXtreme Gradient Boosting. SD=Standard Deviation

## Average F1-scores of classifiers – test size 0.3 (Table A59)

**Table A59:**

**Average F1-scores of classifiers for 12-month mortality prediction on imbalanced data set (test size 0.3)**

| **Test Size** | **Sample Size** | **Number of features** | **Average F1-score (SD)** | | | |
| --- | --- | --- | --- | --- | --- | --- |
|  |  |  | **RLR** | **RF** | **MLP** | **XGBosst** |
| **0.3** | **95042** | **138** | 0.472 (0.004) | 0.177 (0.006) | 0.277 (0.042) | 0.291 (0.006) |
|  |  | **80** | 0.456 (0.007) | 0.158 (0.031) | 0.220 (0.054) | 0.252 (0.024) |
|  |  | **40** | 0.437 (0.010) | 0.152 (0.040) | 0.126 (0.068) | 0.196 (0.039) |
|  |  | **30** | 0.428 (0.011) | 0.160 (0.039) | 0.086 (0.067) | 0.172 (0.043) |
|  |  | **20** | 0.416 (0.013) | 0.185 (0.032) | 0.050 (0.061) | 0.138 (0.047) |
|  |  | **10** | 0.389 (0.024) | 0.121 (0.058) | 0.017 (0.042) | 0.073 (0.048) |
|  | **16000** | **138** | 0.469 (0.010) | 0.156 (0.014) | 0.304 (0.049) | 0.282 (0.014) |
|  |  | **80** | 0.456 (0.012) | 0.144 (0.031) | 0.255 (0.053) | 0.256 (0.024) |
|  |  | **40** | 0.438 (0.013) | 0.145 (0.038) | 0.173 (0.062) | 0.221 (0.031) |
|  |  | **30** | 0.429 (0.014) | 0.147 (0.037) | 0.132 (0.064) | 0.202 (0.035) |
|  |  | **20** | 0.417 (0.016) | 0.176 (0.033) | 0.082 (0.061) | 0.177 (0.037) |
|  |  | **10** | 0.392 (0.026) | 0.154 (0.050) | 0.031 (0.050) | 0.113 (0.050) |
|  | **4000** | **138** | 0.474 (0.020) | 0.162 (0.029) | 0.329 (0.060) | 0.318 (0.028) |
|  |  | **80** | 0.457 (0.022) | 0.149 (0.047) | 0.294 (0.063) | 0.293 (0.035) |
|  |  | **40** | 0.437 (0.024) | 0.146 (0.055) | 0.205 (0.073) | 0.256 (0.044) |
|  |  | **30** | 0.428 (0.023) | 0.148 (0.052) | 0.164 (0.074) | 0.237 (0.044) |
|  |  | **20** | 0.416 (0.027) | 0.170 (0.049) | 0.103 (0.075) | 0.211 (0.048) |
|  |  | **10** | 0.391 (0.033) | 0.174 (0.048) | 0.035 (0.059) | 0.140 (0.060) |
|  | **1000** | **138** | 0.412 (0.040) | 0.074 (0.043) | 0.209 (0.072) | 0.212 (0.055) |
|  |  | **80** | 0.403 (0.041) | 0.075 (0.046) | 0.170 (0.075) | 0.211 (0.058) |
|  |  | **40** | 0.389 (0.042) | 0.094 (0.055) | 0.113 (0.080) | 0.204 (0.060) |
|  |  | **30** | 0.381 (0.043) | 0.108 (0.061) | 0.095 (0.075) | 0.197 (0.063) |
|  |  | **20** | 0.374 (0.044) | 0.131 (0.065) | 0.063 (0.067) | 0.175 (0.066) |
|  |  | **10** | 0.347 (0.069) | 0.156 (0.064) | 0.023 (0.049) | 0.110 (0.072) |

RLR=Regularized Logistic Regression. RF=Random Forest. MLP=Multilayer Perceptron. XGBoost=eXtreme Gradient Boosting. SD=Standard Deviation

## Average accuracies and precisions of classifiers – test size 0.1 (Table A60)

**Table A60: Average accuracies and precisions of classifiers for 12-month mortality prediction on imbalanced data set (test size 0.1)**

| **Test Size** | **Sample Size** | **Number of features** | **Average Accuracy (SD)** | | | | **Average Precision (SD)** | | | |
| --- | --- | --- | --- | --- | --- | --- | --- | --- | --- | --- |
|  |  |  | **RLR** | **RF** | **MLP** | **XGBoost** | **RLR** | **RF** | **MLP** | **XGBoost** |
| **0.1** | **95042** | **138** | 0.701 (0.005) | 0.808 (0.004) | 0.810 (0.004) | 0.812 (0.004) | 0.363 (0.008) | 0.580 (0.026) | 0.561 (0.027) | 0.567 (0.019) |
|  |  | **80** | 0.687 (0.009) | 0.807 (0.004) | 0.808 (0.004) | 0.808 (0.004) | 0.347 (0.010) | 0.559 (0.031) | 0.557 (0.031) | 0.545 (0.023) |
|  |  | **40** | 0.670 (0.013) | 0.802 (0.005) | 0.805 (0.005) | 0.804 (0.004) | 0.330 (0.012) | 0.486 (0.046) | 0.555 (0.054) | 0.514 (0.032) |
|  |  | **30** | 0.662 (0.016) | 0.795 (0.006) | 0.804 (0.004) | 0.803 (0.004) | 0.322 (0.013) | 0.417 (0.049) | 0.549 (0.096) | 0.496 (0.037) |
|  |  | **20** | 0.653 (0.020) | 0.782 (0.007) | 0.804 (0.004) | 0.801 (0.004) | 0.312 (0.015) | 0.352 (0.038) | 0.510 (0.204) | 0.471 (0.046) |
|  |  | **10** | 0.633 (0.037) | 0.787 (0.019) | 0.803 (0.004) | 0.801 (0.004) | 0.292 (0.022) | 0.352 (0.061) | 0.291 (0.325) | 0.427 (0.064) |
|  | **16000** | **138** | 0.700 (0.011) | 0.803 (0.009) | 0.803 (0.009) | 0.802 (0.009) | 0.366 (0.018) | 0.564 (0.067) | 0.538 (0.050) | 0.524 (0.044) |
|  |  | **80** | 0.688 (0.013) | 0.802 (0.010) | 0.802 (0.010) | 0.799 (0.010) | 0.352 (0.020) | 0.544 (0.073) | 0.523 (0.062) | 0.500 (0.049) |
|  |  | **40** | 0.671 (0.016) | 0.798 (0.010) | 0.800 (0.010) | 0.794 (0.010) | 0.336 (0.020) | 0.486 (0.074) | 0.522 (0.089) | 0.465 (0.051) |
|  |  | **30** | 0.664 (0.019) | 0.793 (0.011) | 0.800 (0.010) | 0.793 (0.010) | 0.328 (0.021) | 0.433 (0.072) | 0.516 (0.116) | 0.449 (0.059) |
|  |  | **20** | 0.654 (0.023) | 0.778 (0.012) | 0.799 (0.010) | 0.791 (0.010) | 0.318 (0.022) | 0.355 (0.057) | 0.494 (0.196) | 0.425 (0.062) |
|  |  | **10** | 0.633 (0.041) | 0.777 (0.018) | 0.799 (0.009) | 0.792 (0.010) | 0.297 (0.027) | 0.333 (0.069) | 0.311 (0.326) | 0.389 (0.087) |
|  | **4000** | **138** | 0.702 (0.021) | 0.803 (0.019) | 0.804 (0.019) | 0.801 (0.019) | 0.366 (0.037) | 0.552 (0.133) | 0.531 (0.092) | 0.506 (0.084) |
|  |  | **80** | 0.689 (0.024) | 0.801 (0.019) | 0.801 (0.019) | 0.795 (0.018) | 0.354 (0.036) | 0.520 (0.139) | 0.515 (0.104) | 0.467 (0.087) |
|  |  | **40** | 0.670 (0.026) | 0.799 (0.019) | 0.800 (0.019) | 0.788 (0.020) | 0.335 (0.037) | 0.484 (0.134) | 0.506 (0.132) | 0.427 (0.087) |
|  |  | **30** | 0.664 (0.029) | 0.793 (0.020) | 0.799 (0.019) | 0.786 (0.020) | 0.330 (0.038) | 0.436 (0.126) | 0.499 (0.162) | 0.418 (0.090) |
|  |  | **20** | 0.652 (0.033) | 0.781 (0.021) | 0.800 (0.018) | 0.785 (0.019) | 0.316 (0.038) | 0.359 (0.100) | 0.480 (0.220) | 0.394 (0.092) |
|  |  | **10** | 0.632 (0.053) | 0.773 (0.022) | 0.799 (0.018) | 0.789 (0.019) | 0.299 (0.041) | 0.327 (0.098) | 0.312 (0.341) | 0.381 (0.145) |
|  | **1000** | **138** | 0.728 (0.044) | 0.827 (0.035) | 0.823 (0.036) | 0.820 (0.036) | 0.372 (0.082) | 0.652 (0.245) | 0.588 (0.227) | 0.542 (0.169) |
|  |  | **80** | 0.714 (0.046) | 0.824 (0.036) | 0.823 (0.038) | 0.814 (0.037) | 0.353 (0.080) | 0.606 (0.269) | 0.571 (0.267) | 0.498 (0.171) |
|  |  | **40** | 0.693 (0.051) | 0.818 (0.039) | 0.821 (0.040) | 0.806 (0.038) | 0.330 (0.079) | 0.543 (0.280) | 0.484 (0.361) | 0.460 (0.182) |
|  |  | **30** | 0.683 (0.055) | 0.813 (0.037) | 0.818 (0.040) | 0.802 (0.038) | 0.322 (0.079) | 0.496 (0.269) | 0.386 (0.381) | 0.436 (0.184) |
|  |  | **20** | 0.670 (0.065) | 0.802 (0.040) | 0.818 (0.040) | 0.801 (0.039) | 0.310 (0.082) | 0.413 (0.222) | 0.337 (0.392) | 0.415 (0.195) |
|  |  | **10** | 0.644 (0.094) | 0.788 (0.042) | 0.817 (0.040) | 0.806 (0.040) | 0.289 (0.097) | 0.338 (0.194) | 0.185 (0.344) | 0.384 (0.280) |

## Average accuracies and precisions of classifiers – test size 0.2 (Table A61)

**Table A61: Average accuracies and precisions of classifiers for 12-month mortality prediction on imbalanced data set (test size 0.2)**

| **Test Size** | **Sample Size** | **Number of features** | **Average Accuracy (SD)** | | | | **Average Precision (SD)** | | | |
| --- | --- | --- | --- | --- | --- | --- | --- | --- | --- | --- |
|  |  |  | **RLR** | **RF** | **MLP** | **XGBoost** | **RLR** | **RF** | **MLP** | **XGBoost** |
| **0.2** | **95042** | **138** | 0.702 (0.003) | 0.808 (0.003) | 0.810 (0.003) | 0.811 (0.003) | 0.363 (0.005) | 0.580 (0.018) | 0.561 (0.024) | 0.565 (0.013) |
|  |  | **80** | 0.687 (0.008) | 0.806 (0.003) | 0.808 (0.003) | 0.808 (0.003) | 0.348 (0.008) | 0.558 (0.025) | 0.555 (0.028) | 0.544 (0.019) |
|  |  | **40** | 0.670 (0.013) | 0.802 (0.004) | 0.805 (0.003) | 0.804 (0.003) | 0.330 (0.011) | 0.484 (0.043) | 0.550 (0.041) | 0.509 (0.028) |
|  |  | **30** | 0.663 (0.015) | 0.795 (0.005) | 0.804 (0.003) | 0.803 (0.003) | 0.323 (0.012) | 0.423 (0.049) | 0.550 (0.065) | 0.495 (0.032) |
|  |  | **20** | 0.652 (0.021) | 0.781 (0.006) | 0.803 (0.003) | 0.801 (0.003) | 0.312 (0.014) | 0.349 (0.036) | 0.518 (0.167) | 0.466 (0.040) |
|  |  | **10** | 0.632 (0.039) | 0.789 (0.017) | 0.803 (0.003) | 0.801 (0.003) | 0.291 (0.021) | 0.353 (0.057) | 0.318 (0.312) | 0.424 (0.055) |
|  | **16000** | **138** | 0.696 (0.007) | 0.804 (0.007) | 0.802 (0.007) | 0.804 (0.007) | 0.363 (0.012) | 0.585 (0.048) | 0.529 (0.037) | 0.538 (0.031) |
|  |  | **80** | 0.681 (0.011) | 0.802 (0.006) | 0.801 (0.007) | 0.800 (0.006) | 0.346 (0.014) | 0.560 (0.053) | 0.526 (0.043) | 0.511 (0.033) |
|  |  | **40** | 0.662 (0.014) | 0.798 (0.007) | 0.800 (0.007) | 0.795 (0.007) | 0.327 (0.015) | 0.489 (0.061) | 0.521 (0.059) | 0.464 (0.041) |
|  |  | **30** | 0.656 (0.016) | 0.793 (0.008) | 0.800 (0.007) | 0.793 (0.007) | 0.320 (0.015) | 0.427 (0.063) | 0.521 (0.081) | 0.444 (0.044) |
|  |  | **20** | 0.646 (0.021) | 0.778 (0.009) | 0.799 (0.007) | 0.791 (0.007) | 0.311 (0.017) | 0.351 (0.047) | 0.507 (0.144) | 0.422 (0.048) |
|  |  | **10** | 0.625 (0.041) | 0.777 (0.016) | 0.799 (0.007) | 0.792 (0.007) | 0.292 (0.023) | 0.331 (0.056) | 0.337 (0.310) | 0.393 (0.068) |
|  | **4000** | **138** | 0.690 (0.016) | 0.796 (0.013) | 0.796 (0.014) | 0.791 (0.013) | 0.357 (0.026) | 0.523 (0.098) | 0.512 (0.067) | 0.473 (0.058) |
|  |  | **80** | 0.676 (0.018) | 0.795 (0.013) | 0.794 (0.013) | 0.786 (0.013) | 0.346 (0.026) | 0.512 (0.109) | 0.499 (0.072) | 0.451 (0.061) |
|  |  | **40** | 0.660 (0.022) | 0.792 (0.013) | 0.794 (0.013) | 0.781 (0.013) | 0.331 (0.026) | 0.462 (0.103) | 0.491 (0.096) | 0.415 (0.063) |
|  |  | **30** | 0.653 (0.025) | 0.789 (0.013) | 0.794 (0.013) | 0.780 (0.013) | 0.323 (0.027) | 0.421 (0.097) | 0.491 (0.123) | 0.400 (0.064) |
|  |  | **20** | 0.645 (0.030) | 0.775 (0.015) | 0.794 (0.013) | 0.779 (0.014) | 0.315 (0.028) | 0.351 (0.077) | 0.489 (0.184) | 0.389 (0.066) |
|  |  | **10** | 0.622 (0.052) | 0.767 (0.018) | 0.795 (0.013) | 0.783 (0.014) | 0.296 (0.033) | 0.322 (0.070) | 0.342 (0.342) | 0.374 (0.105) |
|  | **1000** | **138** | 0.674 (0.031) | 0.846 (0.023) | 0.840 (0.024) | 0.831 (0.024) | 0.263 (0.046) | 0.376 (0.423) | 0.402 (0.215) | 0.368 (0.141) |
|  |  | **80** | 0.660 (0.039) | 0.845 (0.022) | 0.841 (0.023) | 0.829 (0.023) | 0.256 (0.046) | 0.332 (0.403) | 0.365 (0.274) | 0.354 (0.144) |
|  |  | **40** | 0.639 (0.045) | 0.842 (0.022) | 0.842 (0.022) | 0.827 (0.023) | 0.246 (0.044) | 0.347 (0.360) | 0.277 (0.313) | 0.333 (0.159) |
|  |  | **30** | 0.630 (0.052) | 0.840 (0.023) | 0.843 (0.023) | 0.827 (0.024) | 0.240 (0.047) | 0.317 (0.314) | 0.224 (0.322) | 0.318 (0.163) |
|  |  | **20** | 0.615 (0.058) | 0.832 (0.024) | 0.845 (0.024) | 0.830 (0.025) | 0.231 (0.046) | 0.270 (0.218) | 0.178 (0.317) | 0.316 (0.188) |
|  |  | **10** | 0.600 (0.088) | 0.817 (0.025) | 0.845 (0.023) | 0.836 (0.023) | 0.222 (0.055) | 0.243 (0.152) | 0.075 (0.235) | 0.273 (0.275) |

## Average accuracies and precisions of classifiers – test size 0.3 (Table A62)

**Table A62: Average accuracies and precisions of classifiers for 12-month mortality prediction on imbalanced data set (test size 0.3)**

| **Test Size** | **Sample Size** | **Number of features** | **Average Accuracy (SD)** | | | | **Average Precision (SD)** | | | |
| --- | --- | --- | --- | --- | --- | --- | --- | --- | --- | --- |
|  |  |  | **RLR** | **RF** | **MLP** | **XGBoost** | **RLR** | **RF** | **MLP** | **XGBoost** |
| **0.3** | **95042** | **138** | 0.701 (0.003) | 0.808 (0.002) | 0.810 (0.002) | 0.811 (0.002) | 0.363 (0.004) | 0.578 (0.014) | 0.557 (0.022) | 0.563 (0.011) |
|  |  | **80** | 0.687 (0.008) | 0.806 (0.003) | 0.808 (0.003) | 0.808 (0.003) | 0.347 (0.007) | 0.558 (0.022) | 0.553 (0.025) | 0.542 (0.016) |
|  |  | **40** | 0.670 (0.013) | 0.802 (0.003) | 0.805 (0.003) | 0.804 (0.003) | 0.330 (0.010) | 0.487 (0.042) | 0.549 (0.032) | 0.508 (0.026) |
|  |  | **30** | 0.663 (0.015) | 0.795 (0.005) | 0.804 (0.003) | 0.802 (0.003) | 0.322 (0.011) | 0.420 (0.047) | 0.548 (0.056) | 0.491 (0.031) |
|  |  | **20** | 0.653 (0.020) | 0.782 (0.006) | 0.804 (0.003) | 0.801 (0.003) | 0.312 (0.013) | 0.352 (0.036) | 0.529 (0.134) | 0.466 (0.037) |
|  |  | **10** | 0.629 (0.040) | 0.787 (0.019) | 0.803 (0.002) | 0.800 (0.003) | 0.290 (0.022) | 0.349 (0.057) | 0.337 (0.301) | 0.421 (0.053) |
|  | **16000** | **138** | 0.702 (0.006) | 0.806 (0.005) | 0.805 (0.006) | 0.806 (0.005) | 0.363 (0.010) | 0.578 (0.039) | 0.531 (0.036) | 0.531 (0.026) |
|  |  | **80** | 0.687 (0.010) | 0.805 (0.005) | 0.804 (0.006) | 0.802 (0.005) | 0.349 (0.012) | 0.557 (0.047) | 0.527 (0.039) | 0.506 (0.030) |
|  |  | **40** | 0.671 (0.014) | 0.801 (0.005) | 0.803 (0.006) | 0.797 (0.006) | 0.332 (0.013) | 0.494 (0.049) | 0.525 (0.048) | 0.462 (0.036) |
|  |  | **30** | 0.663 (0.017) | 0.796 (0.007) | 0.803 (0.006) | 0.795 (0.006) | 0.324 (0.014) | 0.430 (0.057) | 0.523 (0.063) | 0.441 (0.040) |
|  |  | **20** | 0.654 (0.022) | 0.782 (0.008) | 0.802 (0.005) | 0.793 (0.005) | 0.314 (0.016) | 0.352 (0.044) | 0.522 (0.114) | 0.418 (0.042) |
|  |  | **10** | 0.632 (0.041) | 0.779 (0.016) | 0.802 (0.005) | 0.795 (0.006) | 0.294 (0.023) | 0.330 (0.052) | 0.390 (0.281) | 0.395 (0.064) |
|  | **4000** | **138** | 0.702 (0.012) | 0.805 (0.010) | 0.806 (0.010) | 0.805 (0.009) | 0.367 (0.020) | 0.585 (0.075) | 0.547 (0.060) | 0.532 (0.045) |
|  |  | **80** | 0.685 (0.017) | 0.804 (0.010) | 0.804 (0.011) | 0.799 (0.010) | 0.349 (0.021) | 0.558 (0.085) | 0.536 (0.066) | 0.496 (0.050) |
|  |  | **40** | 0.663 (0.021) | 0.799 (0.011) | 0.802 (0.012) | 0.790 (0.011) | 0.329 (0.023) | 0.485 (0.093) | 0.524 (0.086) | 0.438 (0.057) |
|  |  | **30** | 0.654 (0.022) | 0.795 (0.011) | 0.801 (0.011) | 0.786 (0.011) | 0.320 (0.021) | 0.434 (0.087) | 0.518 (0.103) | 0.412 (0.057) |
|  |  | **20** | 0.643 (0.029) | 0.781 (0.013) | 0.801 (0.011) | 0.784 (0.011) | 0.310 (0.025) | 0.356 (0.075) | 0.515 (0.160) | 0.392 (0.065) |
|  |  | **10** | 0.623 (0.044) | 0.771 (0.016) | 0.800 (0.010) | 0.789 (0.012) | 0.291 (0.027) | 0.318 (0.064) | 0.349 (0.323) | 0.376 (0.093) |
|  | **1000** | **138** | 0.653 (0.027) | 0.803 (0.018) | 0.803 (0.019) | 0.783 (0.019) | 0.307 (0.037) | 0.407 (0.225) | 0.478 (0.133) | 0.361 (0.087) |
|  |  | **80** | 0.640 (0.030) | 0.802 (0.019) | 0.803 (0.019) | 0.782 (0.021) | 0.298 (0.037) | 0.406 (0.224) | 0.471 (0.169) | 0.357 (0.091) |
|  |  | **40** | 0.622 (0.037) | 0.801 (0.019) | 0.805 (0.020) | 0.781 (0.021) | 0.284 (0.037) | 0.400 (0.210) | 0.407 (0.248) | 0.351 (0.097) |
|  |  | **30** | 0.616 (0.042) | 0.799 (0.019) | 0.807 (0.019) | 0.782 (0.020) | 0.277 (0.037) | 0.364 (0.166) | 0.407 (0.270) | 0.340 (0.096) |
|  |  | **20** | 0.602 (0.059) | 0.788 (0.021) | 0.806 (0.020) | 0.784 (0.021) | 0.272 (0.039) | 0.314 (0.135) | 0.348 (0.313) | 0.335 (0.111) |
|  |  | **10** | 0.589 (0.093) | 0.770 (0.022) | 0.808 (0.019) | 0.794 (0.020) | 0.258 (0.059) | 0.270 (0.101) | 0.181 (0.322) | 0.323 (0.186) |

1. ‘Recent Falls’ was removed due to excessive missing values. The change in coding for falls caused the large number of missing data in ‘Recent Falls’. We included ‘Falls’ instead of ‘Recent Falls’ in our study. [↑](#footnote-ref-2)
